# A Generative AI‐Assisted Piezo‐MEMS Ultrasound Device for Plant Dehydration Monitoring

**DOI:** 10.1002/advs.202504954

**Published:** 2025-06-19

**Authors:** Kaustav Roy, Darren Sim, Luwei Wang, Zixuan Zhang, Xinge Guo, Yao Zhu, Sanjay Swarup, Chengkuo Lee

**Affiliations:** ^1^ Department of Electrical and Computer Engineering National University of Singapore Singapore 117576 Singapore; ^2^ Center for Intelligent Sensors and MEMS (CISM) National University of Singapore Singapore 117583 Singapore; ^3^ Department of Biological Sciences National University of Singapore Singapore 117543 Singapore; ^4^ Institute of Microelectronics (IME) Agency for Science Technology and Research (A*STAR) Singapore 117685 Singapore

**Keywords:** generative AI, microelectromechanical Systems, piezoelectricity, plant health, wearable

## Abstract

Plant health, closely tied to hydration, has a direct impact on agricultural productivity, making the monitoring of leaf water content essential. Current devices, however, are often invasive, bulky, slow, power‐inefficient, Complementary Metal‐Oxide‐Semiconductor (CMOS)‐incompatible, and unsuitable for large‐scale, re‐usable outdoor sensor networks. Utilizing micro‐electromechanical systems (MEMS) fabrication enables wafer‐scale miniaturization and precise control of ultrasound transducers, thereby enhancing sensitivity while significantly reducing power and cost. This work introduces the CMOS‐compatible, plant‐leaf attachable piezo‐MEMS ultrasound device (PMUT‐Leaf‐PMUT, PLP) for real‐time dynamic moisture monitoring and rapid one‐shot measurement of relative water content (RWC). Notably, the PLP is reattachable to pre‐calibrated plant leaves, enhancing reusability and reducing electronic waste. Employing piezoelectric micromachined ultrasound transducers (PMUTs) fabricated via piezoelectric over silicon‐on‐nothing (PSON), the device non‐invasively monitors hydration across diverse cultivars with a 70% relative water content (RWC) detection range. Generative deep learning using a conditional variational autoencoder (CVAE) translates electrical signals to precise hydration measurements, achieving an RWC root‐mean‐square error of 1.25%. The deployment of this generative AI‐assisted PLP system directly links plant responses to environmental shifts, representing a significant advancement in precision plant health management and irrigation practices, thereby substantially improving agricultural efficiency and promoting environmental conservation.

## Graphical abstract

This study introduces a complementary metal–oxide–semiconductor (CMOS)‐compatible wearable piezoelectric micromachined ultrasound transducers (PMUTs) that sandwiches the leaf (PMUT–Leaf–PMUT, PLP) and employs generative artificial intelligence for real‐time analysis. Using relative water content (RWC) as a physiological marker, the system continuously links plant responses to environmental shifts, enabling precise phenotyping, crop performance assessment, and sustainable, smart irrigation agriculture.

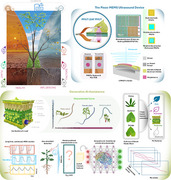

## 1 Introduction

Doubling crop production by 2050 is crucial to meet the anticipated future food demands.^[^ ^1^ ^]^ However, present agricultural practices encounter significant challenges like climate change, water scarcity, and fertile land unavailability resulting in substantial agricultural losses.^[^ ^2^, ^3^ ^]^ Today's food shortages demand an urgent need for sustainable agricultural innovations. Innovations like vertical farming and precision agriculture hold promise. Vertical farming^[^ ^4^ ^]^ addresses the scarcity of fertile land and meets the increasing food demand without exacerbating environmental degradation. Conversely, precision agriculture leverages advanced technologies and strategies to enhance yields, employing novel sensing technologies, such as plant‐wearable electronics/sensors,^[^ ^5^ ^]^ intelligent data analytics,^[^ ^6^ ^]^ and global sensor connectivity in the form of Internet‐of‐Things (IoT),^[^ ^7^ ^]^ to improve crop management.^[^ ^8^ ^]^ These advanced sensor systems generally allow for noninvasive plant health monitoring, facilitating early stress detection,^[^ ^9^, ^10^ ^]^

Flexible/wearable sensors^[^ ^11^ ^]^ have been predominantly used in human health monitoring to track cardiovascular vitals^[^ ^12^, ^13^, ^14^, ^15^ ^]^ body temperature,^[^ ^16^, ^17^ ^]^ sweat‐biomarkers^[^ ^18^, ^19^, ^20^ ^]^ and even sweating rate.^[^ ^21^, ^22^ ^]^ Building on these developments, flexible/wearable sensors are now being adapted for agricultural use, particularly for monitoring plant health,^[^ ^23^, ^24^ ^]^ These sensors are capable of detecting plant growth,^[^ ^25^, ^26^ ^]^ volatile organic compounds (VOCs) in plant signaling,^[^ ^27^, ^28^ ^]^ and predicting ambient conditions affecting plant growth.^[^ ^29^, ^30^ ^]^ However, these sensors typically rely on changes in resistance, capacitance, and inductance as sensing parameters and lack reusability and re‐attachability, limiting their use to single occasions.^[^ ^24^, ^31^ ^]^ Being able to detach and reattach the sensors allows them to be reused on multiple plants or at different growth stages of one plant, making the technology more sustainable, quicker to deploy, and less expensive. Consequently, there is an urgent need to develop such solutions to enhance agricultural practice efficiency, accompanied by the necessity for faster, noninvasive sensing of plant variables for timely damage mitigation. Recent advancements witness machine learning (ML) applied in precision agriculture for plant stress phenotyping to identify^[^ ^32^, ^33^, ^34^, ^35^ ^]^ and classify,^[^ ^36^, ^37^ ^]^ various plant stresses and diseases via imaging modalities.^[^ ^38^ ^]^ Nonetheless, the quantitative prediction of stress phenotyping using ML, especially in relation to relative water content (RWC) as a deep‐tissue indicator, remains a challenge that urgently requires attention.

Tightly regulating water availability during vegetative and seed formation stages is crucial, and plant‐wearables can enhance management capabilities. Monitoring RWC offers a straightforward yet effective approach for stress phenotyping, guiding optimal irrigation practices to reduce water waste.^[^ ^39^, ^40^, ^41^, ^42^ ^]^ RWC provides insights into plants' hydration needs, growth phases, and health, reacting to stressors like insufficient watering, saline soil, or harsh weather. However, traditional RWC measurement methods are destructive, hindering the ability to study plants' real‐time water responses. Consequently, there is a pressing demand for rapid, non‐invasive, and lightweight plant‐wearables that allow researchers and agronomists to analyze water‐related traits efficiently. Although several techniques for gauging RWC exist, most fail to meet these requirements. Early techniques, such as using a micrometer^[^ ^43^ ^]^ to gauge leaf thickness, proved invasive and unreliable due to leaf thickness variations. Subsequent less‐invasive methods focused on spectral analysis, including infrared reflectance,^[^ ^44^ ^]^ infrared thermometry,^[^ ^45^ ^]^ and canopy reflectance^[^ ^46^ ^]^ to assess water status. However, their precision is limited by plant architecture, rendering these techniques primarily suitable for crop science applications.^[^ ^47^ ^]^

The sensor community has recently witnessed a giant boost from AIOT assistance^[^ ^48^, ^49^, ^50^, ^51^, ^52^, ^53^, ^54^, ^55^, ^56^, ^57^, ^58^, ^59^, ^60^, ^61^, ^62^, ^63^, ^64^ ^]^ and there has been a recent surge in the development of flexible and bio‐adhesive bulk ultrasound transducers^[^ ^65^, ^66^, ^67^, ^68^, ^69^ ^]^ for human deep‐tissue sensing/imaging applications, such as the wearable cardiac imager,^[^ ^70^ ^]^ photoacoustic patch,^[^ ^71^ ^]^ on‐skin bladder imager,^[^ ^72^ ^]^ and shear wave elastography imagers,^[^ ^73^, ^74^ ^]^ Because these transducers are optimized for human deep‐tissue imaging, they operate at higher power levels than necessary, rendering them energy‐inefficient for monitoring the much thinner tissues found in plants. Despite this disadvantage, high‐power, bulk ultrasound‐based rigid systems have been used to monitor plant RWC^[^ ^75^, ^76^, ^77^, ^78^, ^79^ ^]^ indicating the demand for ultrasound as a non‐invasive plant tissue probe. Despite advances in ultrasound‐based RWC monitoring, deploying current transducers outdoors remains challenging because they are large, bulky, and consume substantial power. Moreover, the deterministic approach of deep learning algorithms in the non‐generative model used for predicting RWC can lead to significant errors, exacerbated by leaves' sensitive reactions to minor RWC variations. Additionally, the extensive use of these sensors alongside learning algorithms to investigate RWC dynamics in controlled settings, such as growth chambers, remains underexplored.

Therefore, the unmet needs outlined above necessitate the development of a fast, low‐powered, CMOS‐compatible, quantitative AI‐assisted, noninvasive, plant‐wearable, and re‐attachable multimodal sensor capable of tracking minute changes in leaf RWC. To address this, we introduce a quasi‐flexible thin‐film piezoelectric micromachined ultrasound transducer (PMUT)^[^ ^80^, ^81^, ^82^, ^83^, ^84^, ^85^, ^86^, ^87^, ^88^, ^89^, ^90^, ^91^, ^92^, ^93^, ^94^, ^95^, ^96^, ^97^, ^98^, ^99^, ^100^, ^101^, ^102^, ^103^ ^]^ based clip that incorporates all the aforementioned advantages in a single sensor package. This device employs multimodality by combining the domain of bioacoustics and image‐enhanced machine intelligence in the form of generative AI to probe and extract RWC from within a leaf attached to a whole plant. Operating on air‐coupled PMUTs, the device accurately detects real‐time moisture content in various substrates and effectively assesses water content in detached leaves. Incorporating a trained conditional variational autoencoder (CVAE) from 200 leaf samples, the system generates unique curves linking sensor signals to leaf hydration levels. Tested on three whole plants, the sensor continuously monitors hydration, revealing correlations with environmental variables like temperature, light, moisture, and irrigation presence. Thus, this technology demonstrates significant potential in advanced plant health monitoring and environmental response analysis. The novelty of our work, and its impact, have been further elaborated in Note SI (Supporting Information), and two separate depictions: **Tables** **1** and **2** are provided to compare our work with the major contributions in the field, highlighting key advancements in methodology, performance metrics, and overall significance in relation to prior studies.

**Table 1:** Comparison of work with all the major advancements in the domain of RWC measurement.

Author(s)	Physics	Device Modality	Plant	RWC detection range	RWC detection sensitivity	Non‐invasive?	Leaf attachable?	AI Enabled?	CMOS Compatible?	Ref.	Device Picture
**Alvarez et al**.	Broadband spectroscopy technique	In‐air ultrasound	Prunus laurocerasus	2.6%	–	No	No	No	No	[110]	Not available
**Kim et al**.	Electrical impedance spectroscopy	Conductive electrodes	Pothos	13%	–	Yes	Yes	No	No	[111]	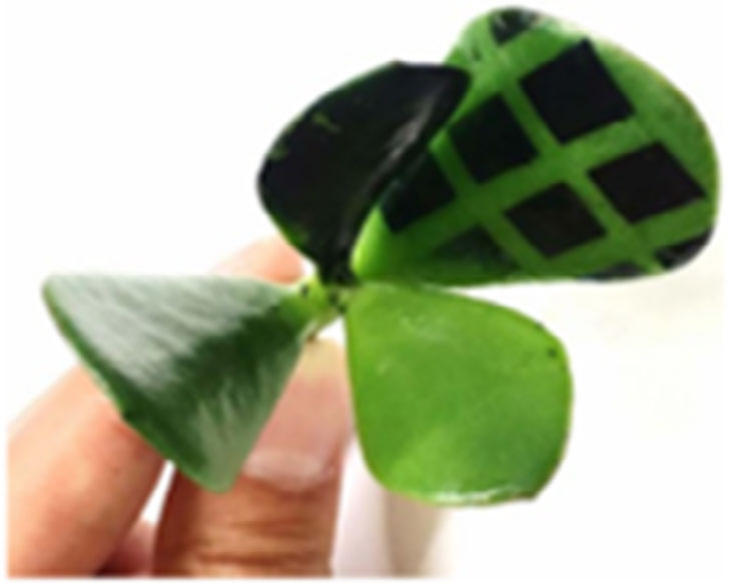
** Zimmermann et al**.	Pressure detection	Pressure sensor	Tetrastigma voinierianum	–	–	Yes	Yes	No	No	[112]	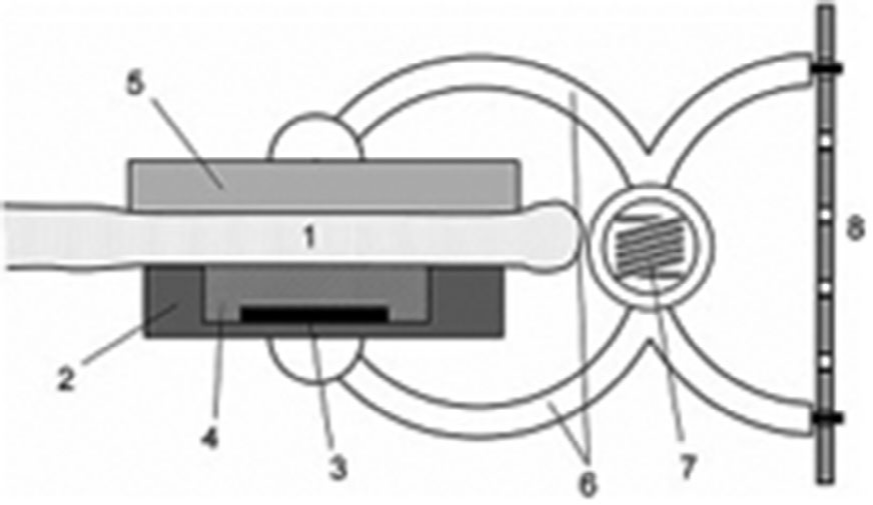
**Hunt et al**.	Beer Lambert's law	Infrared reflectance	Agave deserti	80%	–	No	No	No	No	[113]	Not available
**Farinas et al**.	Resonant ultrasound spectroscopy	In‐air ultrasound	Viburnum tinus	28%	–	Yes	No	Yes	No	[114]	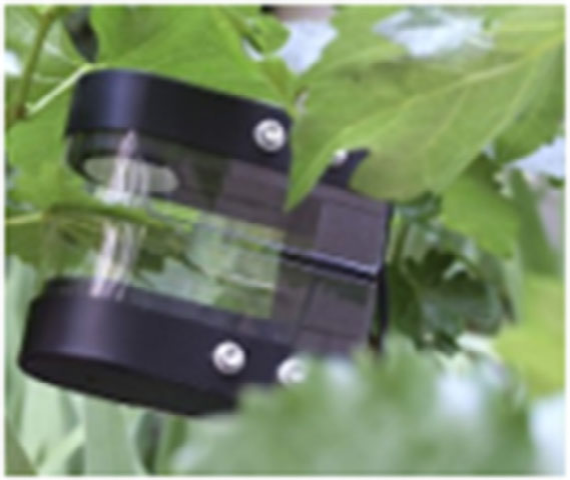
**Atherton et al**.	Thermal resistance	Thermal sensor	Lactuca sativa	22%	≈5%/°C	Yes	Yes	No	No	[115]	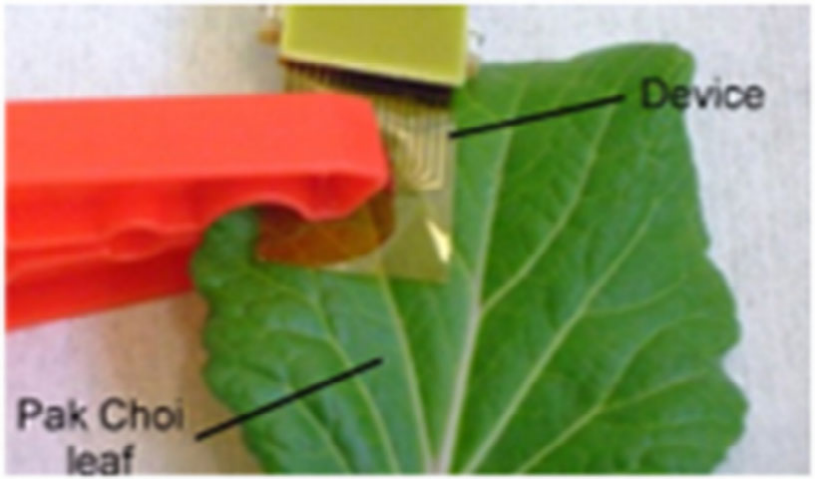
**Shihao Yin and Liang Dong**	Electrical impedance spectroscopy	Conductive electrodes	Zea mays	37%	≈‐8.75 x 10^−6^%/Ω	Yes	Yes	No	No	[116]	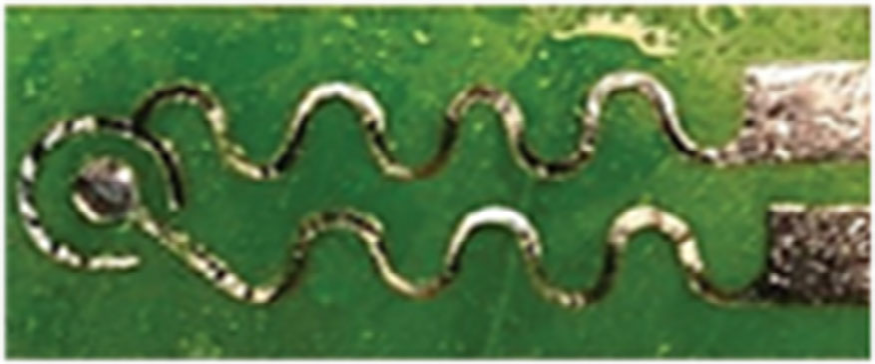
**Li et al**.	Beer Lambert's law	Terahertz transmittance	Sloanea australis	–	–	Yes	No	No	No	[117]	Not available
**Zheng et al**.	Electrical impedance	4‐point electrical probe	Zea mays	80%	≈‐37%/V	No	Yes	No	No	[118]	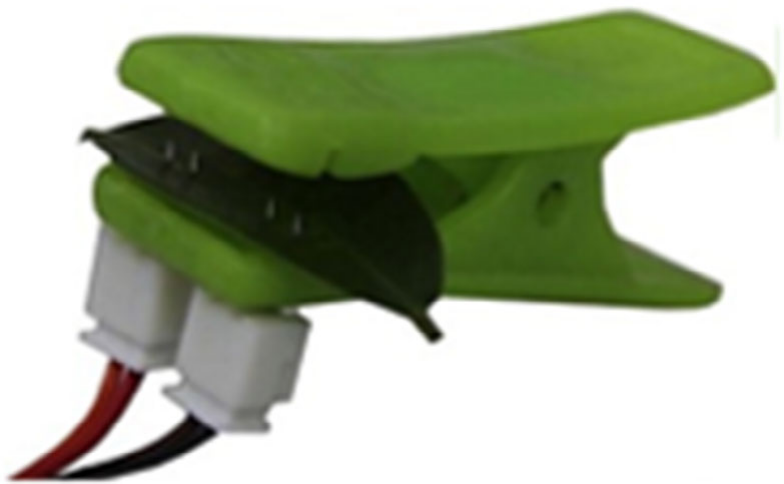
**Afzal et al**.	Thickness	Magnetic thickness sensor	Zea mays	80%	–	No	No	No	No	[119]	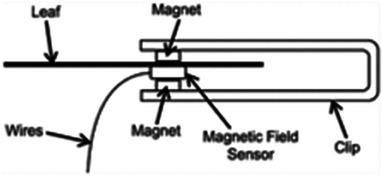
**Roy et al**. **(this work)**	Sound transmission	In‐air ultrasound	Ipomoea Batatas	70%	≈‐ 0.7%/mV	Yes	Yes	Yes	Yes		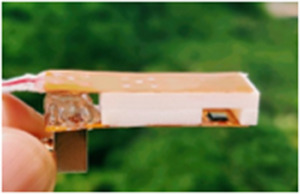

**Table 2:** A comparison of works on AI enhanced PMUTs.

Author	PMUT frequency	PMUT bandwidth/sensitivity	Operating medium	ML model used	Application	Refs.
Liu et al.	302 kHz, operating at 10 Hz – 20 kHz	wideband	Fluid coupled	Non‐generative deep learning: CNN	Speech recognition	[120]
Paramanick et al.	1 MHz	30% (fractional BW)	Fluid coupled	Non‐generative deep learning: CNN (U‐Net)	Photoacoustic image quality enhancement	[97]
**Yue et al**.	50 kHz	‐	Air coupled	Non‐generative deep reinforcement learning	Focused acoustic pressure optimization	[121]
**Roy et al. (this work)**	1.3 MHz	350 nm V^−1^	Air coupled	Generative deep learning: CVAE	Crop health monitoring	

### 1.1 The Concept of Generative AI‐Assisted Piezo‐MEMS Ultrasound Platform

**Figure 1:**
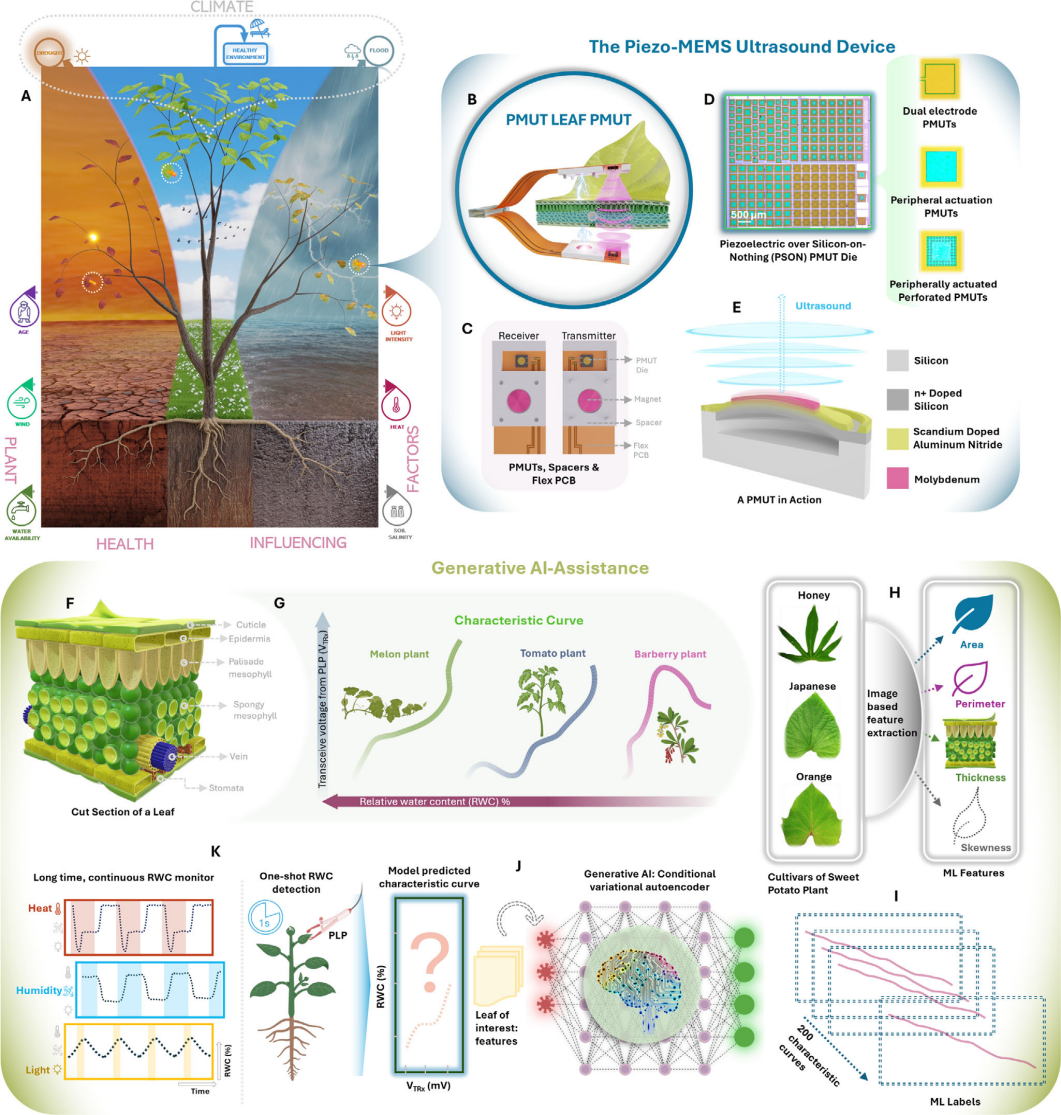
Concept abstraction depicting the idea behind the contribution. A) A picture showing a plant in three different climatic conditions: drought, normalcy, and flood along with the factors which are closely related to the climate, influencing the plant health. Some of such factors are plant age, prevailing wind condition, water availability, soil salinity, heat, and light intensity. The plant behaves differently when exposed to such climatic conditions, and it is possible to understand those behaviors by measuring plant phenotypes. Subfigures **B‐E** describes our piezo‐MEMS ultrasound device: B) Leaves (circled) are clipped with the PLP wearable—comprising a transmitter and receiver arm—that sends ultrasound through the leaf cross‐section. Water‐induced attenuation reduces the transmitted signal, so the receiver's voltage amplitude directly reflects leaf water volume. C) The constituents of the PLP system made up of two PMUT dies, male (transmitter) and female (receiver) spacers and two flex PCBs. D) Looks of each PMUT die containing 3 different kinds of PMUT cells: the dual electrode PMUT, peripherally actuated PMUT and peripherally actuated perforated PMUT, fabricated following the novel piezoelectric over silicon‐on‐nothing process (PSON). E) A vibrating ScAlN‐PSON PMUT generates ultrasound; the 3‐D cutaway reveals its silicon structure, n‐doped silicon ground, ScAlN piezo layer, and molybdenum signal electrode. Figure 1 (continued). Concept abstraction depicting the idea behind the contribution. Subfigures **F‐K** describes the assistance that generative AI provides to make the ultrasound detection of RWC generic: F) Cross‐section of a leaf, highlighting its layers and the spongy mesophyll where mobile water resides, which determines wilting. G) A hypothetical plot shows PLP transceive voltage (V_TRx_) versus leaf RWC: as RWC falls, ultrasound transmission—and thus V_TRx_—rises. Each plant type has its own “characteristic curve,” illustrated here for melon, tomato, and barberry. H) Sweet potato, chosen for its agricultural and nutritional importance, was tested in three cultivars—Honey, Japanese, and Orange—whose leaves exhibit distinct geometries (area, perimeter, thickness, skewness) that serve as AI‐readable tags. I) In order to attach the tags to labels, we used characteristic curves obtained from 200 different leaves. Thus, we had 200 different sets of features which corresponded to 200 different characteristic curves. These data formed the training data library. J) A conditional variational autoencoder was used to generate a unique V_TRx_–RWC characteristic curve for each leaf based on its features, linking PLP voltage readings directly to that leaf's hydration level. K) Once the AI‐generated characteristic curve is available, the PLP can (a) perform an immediate, one‐shot RWC measurement upon attachment and (b) continuously track RWC over extended periods, reporting plant responses to heat, humidity, light, and irrigation.

A plant's water status, impacted by environmental interactions such as soil water uptake and transpiration, influenced by leaf, root structures, and environmental factors (e.g., sunlight, temperature), is conceptualized in **Figure** **1**A, showing a tree under various climates: drought, normalcy, and flood. The PMUT‐based PLP sensor comprising a dual‐section flexible printed circuit board (PCB) assembly is proposed for continuously monitoring the RWC, as shown in Figure 1B. It features two flexible arms, each arm outfitted with an optimized spacer with diagonal snug‐fit interlocks and a central magnetic core (see Figure 1C), enhancing the acoustic response consistency of the PLP. Each spacer houses a MEMS ultrasound die hosting three kinds of PMUT cells, including dual electrode,^[^ ^85^, ^104^, ^105^ ^]^ peripherally actuated, and peripherally actuated perforated PMUTs.^[^ ^106^ ^]^ The PMUT chip nomenclature is depicted in Note SII‐A (Supporting Information). These cells form four elements, as depicted in Figure 1D, with the most acoustically responsive element chosen for the device, detailed in Note SII‐B (Supporting Information).

The PMUTs in our study, fabricated using a novel PSON process, are detailed in the methods section and Note SII‐C (Supporting Information). Figure 1E shows a 3D cut‐section of a PMUT in action, with its layers, including a 15% ScAlN on a p‐doped silicon base. The PMUT's vibration mechanism, influenced by the piezoelectric thin film's d_31,_ *_f_* coefficient, is illustrated in Note SII‐D (Supporting Information). Applying the PLP sensor to leaves involves understanding their complex structure, including layers such as cuticle, epidermis, and mesophyll, as well as veins and stomata, as shown in Figure 1F. The PLP measures hydration by sending sound waves through the leaf, with sound transmission affected by the ratio of water‐to‐air voids within the leaf's structure, directly correlating to the leaf's RWC.

Different plant species, due to their unique leaf architectures, display varied material properties like density, bulk modulus, and ultrasound attenuation coefficient, as well as differing air void volumes.^[^ ^107^ ^]^ Additionally, leaves from the same plant can vary in layer composition based on age and environmental conditions during growth, leading to diverse structures. Consequently, each leaf has a distinct relationship between V_TRx_ and its RWC, resulting in a unique each leaf has a distinct relationship between V_TRx_ and its RWC, resulting in a unique characteristic curve for each leaf (see Figure 1G).

Leaf morphologies vary significantly across and within plant species, influencing their characteristic curves. We used the sweet potato plant to study the impact of leaf geometry on hydration detection, analyzing traits like area, perimeter, thickness, and skewness and their correlation with characteristic curves for various cultivars^[^ ^108^ ^]^ (Figure 1H,I). A deep, conditional variational autoencoder (CVAE) model (Figure 1J) was developed, leveraging these geometric traits to predict characteristic curves for unseen leaves, translating PLP voltage readings into real‐time RWC values. The characteristic curves as predicted by our model were found to closely correlate with the unseen curves as obtained by feeding unknown leaf features into the model, indicating the efficacy of AI assistance to serve as a generalized translator for sensor readout to plant phenotypic parameters, such as RWC in this case. Subsequently, this approach was validated in a growth chamber for one‐shot RWC measurement as well as long‐time dynamic RWC tracking with simulated conditions, proving its accuracy in monitoring plant hydration and health (Figure 1K).

### 1.2 The PMUT‐Leaf‐PMUT (PLP) as a Water Content Measuring Device

**Figure 2:**
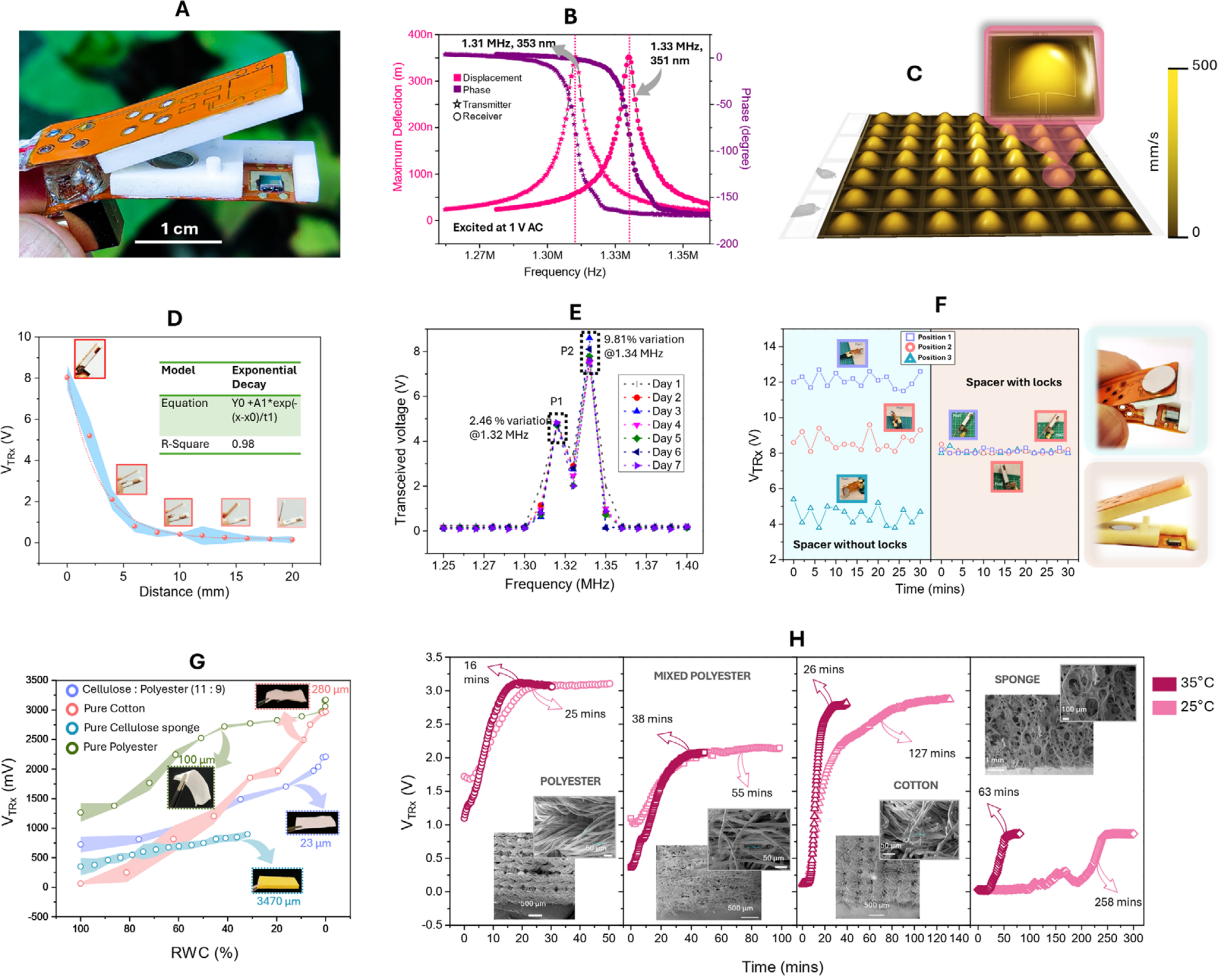
The PMUT‐Leaf‐PMUT (PLP). A) Picture of a typical PMUT‐Leaf‐PMUT (PLP). B) Peak hold deflection and phase spectra for 1 of the 49 transmitter and receiver cells and as obtained using the laser doppler vibrometer. Results depict resonant frequencies of 1.31 MHz for the transmitter and 1.33 MHz for the receiver, both with high deflection sensitivities (353 nm V^−1^ for transmitter and 351 nm V^−1^ for receiver) and ideal resonance characteristics. C) A scanning vibrometry snapshot of the transmitter devices when operated with 1 V AC. PMUTs operate in a modified‐parabolic mode at fundamental resonance (as shown in the inset) where all cells resonate in phase, reaching a maximum velocity of 500 mm s^−1^. D) Sound attenuation in air for PLP, when the distance between the flexible arms of the PLP is varied by 2 mm distance from 0 to 20 mm; transmitter element was operated at the resonance range of 1.32 MHz. An exponentially wavy decay was observed with distance, the waviness created due to generation of standing waves owing to multiple reflection of sound at the solid spacer walls. E) Results depicting the one‐week frequency sweep experiment at the resonance range to detect the time‐repeatability of the PLP. Two distinct resonant peaks were observed. Peak 1 at 1.32 MHz had a mean amplitude of 4.7 V with a 2.5% variation, while Peak 2 at 1.34 MHz showed 7.92 V with a 9.8% variation. F) Spacer architecture importance on the PLP response. Two different kinds of spacers were made: (a) without locks and an extra‐structure magnetic fastener, and another with locks and an intra‐structure magnetic fastener and PLP's functioning was tested using both the spacers. The non‐locking spacer resulted in non‐repetitive performance, with three varying mean V_TRx_ values (5, 9, and 12 V) and ≈50% V_TRx_ variation with time. In contrast, the locking spacer maintained a consistent 8 V V_TRx_ regardless of position and showed negligible time variation, thereby improving PLP's repeatability. G) Characteristic curves (V_TRx_ versus RWC) as obtained from various porous materials, having different thickness, material composition and void ratios: cotton (thickness: 280 µm), polyester (thickness: 100 µm), cellulose sponge (thickness: 3470 µm), and cellulose‐polyester blend (thickness: 23 µm). Results suggest the uniqueness of the PLP to detect moisture content across different fabrics and porous materials. H) Drying time dynamics for the 4 wet porous materials which closely mimic a leaf if it behaves as a tissue‐air composite. The result shows difference in the drying trends at two different temperatures: 25 °C and 35 °C, as captured by using the PLP.

The PLP device, featuring a transmitter (T_x_) and receiver (R_x_) PMUT on opposing flex PCBs, assesses water status through sound transmission, with receiver voltage amplitude (V_TRx_) being the indicator (**Figure** **2**A). The PMUTs are subsequently evaluated for their electro‐mechanical properties using a Laser Doppler Vibrometer (LDV). We have used the dual‐electrode element with a 7 × 7 cell array. One cell from both the Tx‐Rx die is characterized using the LDV; results show closely spaced resonant peaks, which results in a highly sensitive T_x_‐R_x_ device (Figure 2B). Additional resonant response characterization is reported in Note SII‐E,F (Supporting Information) respectively. A scanning vibrometry result depicts cells in the T_x_ element operating in a modified‐parabolic mode at fundamental resonance,^[^ ^109^ ^]^ (Figure 2C), wherein every cell resonates in phase, indicative of perfectly connected wire traces and uniformity in the ScAlN layer deposition. The process flow for making the PLP is elaborated in Note SII‐G (Supporting Information).

PLP's acoustic performance was tested by varying the distance between its arms by 2 mm increments to observe the sound attenuation pattern (Figure 2D). V_TRx_ after a charge amplification (10 V pC^−1^) from the HQA‐15M‐10T amplifier was observed to exponentially decay (from 8 V to 120 mV) with distance. This pattern, though not perfectly matching the expected hyperbolic plus exponential reduction from spreading and absorption losses, follows a similar trend, with deviations possibly due to sound diffraction and resonant cavity effects altering the anticipated loss pattern.

To ensure the PLP's time‐based repeatability, we conducted a one‐week frequency sweep (1.25 to 1.40 MHz), observing V_TRx_ consistency (Figure 2E). Two peaks were observed, with the first peak having a mean amplitude of 4.7 V with a 2.5% variation, while the second having a mean amplitude of 7.92 V with a 9.8% variation. The formation of dual peaks may be a result of a standing wave pattern formation in the spacer cavity owing to multiple reflections. Although the second peak was 1.7 times stronger than the first, its time‐variability was higher, which was considered detrimental for the machine learning integration with the PLP, which functions better with repeatable data. Thus, the first peak at 1.32 MHz was chosen for all the subsequent experiments.

Spatial repeatability was tested by optimizing the spacer architecture, crucial for consistent measurements due to the PLP's reliance on the acoustic field in the quasi‐acoustic cavity (Note SII‐H, Supporting Information). Two different spacer designs were evaluated: one without locks using an extra‐structure magnetic fastener, and another with locks and an intra‐structure magnetic fastener. The non‐locking spacer resulted in non‐repetitive performance with space, with 67% and 50% variation of V_TRx_ with space and time respectively. In contrast, the locking spacer maintained a consistent V_TRx_ level regardless of the space and time. This is mostly because the non‐locking spacer offered more degree of freedom in the sliding of the PLP's arms against one another resulting in the change of acoustic field pattern inside the spacer cavity which resulted in the V_TRx_ variation. On the contrary, the locking spacer restricts any sliding movement resulting in repeatability. Figure 2F demonstrates the importance of the spacer architecture for PLP's performance. After optimizing the spacer, it was important to observe the through transmission acoustics of the PLP as elaborated in Note SII‐I (Supporting Information).

PLP's efficacy in detecting water content was tested on diverse porous materials to mimic plant leaf structures (Figure 2G), choosing materials like cotton, polyester, cellulose sponge, and a cellulose‐polyester blend, each representing different compositions and void ratios (see Experimental Section for experimental protocol). Operating PLP at 5 V, we mapped characteristic curves linking V_TRx_ to RWC, finding significant variations across the materials. Thin polyester showed the steepest V_TRx_‐RWC slope, and the thick cellulose sponge the gentlest. This indicates that material composition, thickness, and air‐void density impact the characteristic curve. While a detailed analysis is outside this work's focus, the experiments emphasize three messages: characteristic curves are non‐linear, dependent on physical material properties and distribution, and serve as calibration references for hydration level assessments.

In drying tests on the above materials at 25 °C and 35 °C within a Jiupo Inc. growth chamber (Figure 2H), drying times spanned from 25 to 258 min at 25 °C and 16 to 63 min at 35 °C, with higher temperatures notably reducing drying times for cotton and cellulose sponge. PLP detected faster drying rates at 35 °C across all materials, evidenced by steeper V_TRx_ slopes. Initial voltage differences in polyester and mixed polyester were linked to varying initial drying times (2 min at 25 °C and 4 min at 35 °C). Scanning electron micrographs (SEM) of each textile revealed differences in thread diameter, knitting patterns, and void density. Thread thickness was measured at 16, 11, and 10 µm for polyester, polyester mix, and cotton, respectively, while the sponge had solid material with internal voids. The experiments suggest (a) drying dynamics is influenced by thread wettability, void ratio, and textile thickness, (b) PLP has the capability as a dynamic water content tracker. The comparison between porous structures and leaves was drawn because both are composite structures composed of solid components and air voids. In both systems, these air voids can be filled with water, and the sound propagation through the composite is affected by the water content.

To understand the functioning of the PMUTs and the PLP, a finite element simulation using COMSOL Multiphysics has been carried out as elaborated in Note SII‐J—L (Supporting Information). The setup used to record RWC is depicted in Note SII‐M (Supporting Information).

### 1.3 PLP on Detached Leaves: A Re‐attachable RWC Tracker

**Figure 3:**
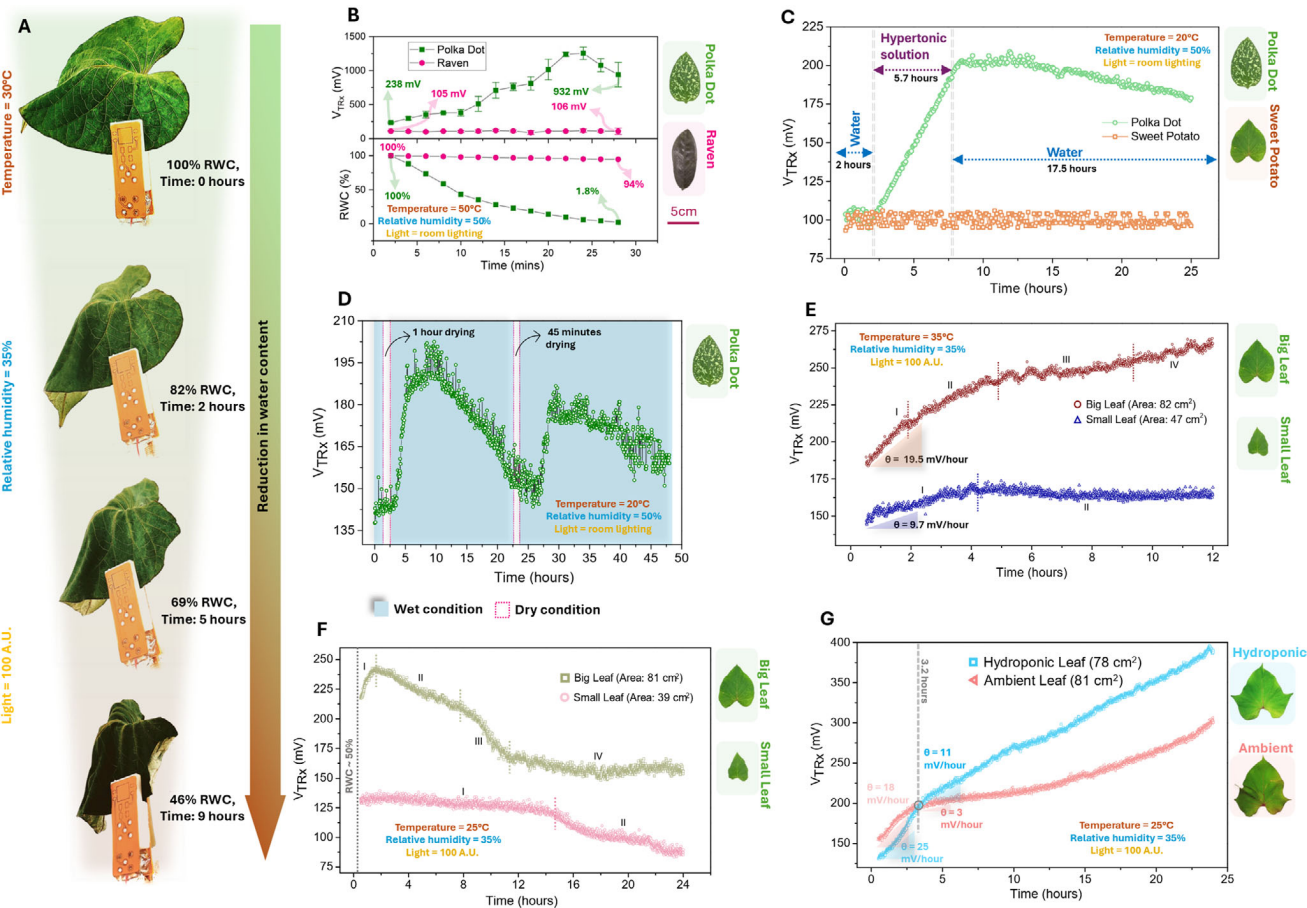
PLP on detached leaves. A) A time lapse snapshot of a separated sweet potato leaf with PLP attached on it, proving the attachability of the PLP to both turgid and wilted leaves. The PLP is always attached on the leaf blade toward the leaf margin to avoid any unnecessary reflections from the midrib. B) Water loss dynamics as observed using the PLP from dehydration‐sensitive (HP or polka dot) and dehydration‐insensitive (ZZ or raven) plants. For the HP leaf, RWC drastically decreased from 100% to 1.8%, with a corresponding V_TRx_ increase from 238 to 932 mV. In contrast, the ZZ leaf showed a minor RWC decrease from 100% to 94%, and a negligible V_TRx_ change from 105 to 106 mV, revealing the existence of the genotypical feature such as drought resistance even at singular leaf level. C) Osmotic time dynamics as observed from two different plant leaves: HP and JSP, by probing the leaves with PLP at the singular leaf level. The JSP leaf did not exhibit any response, however the HP leaf demonstrated significant fluctuations. For HP, V_TRx_ rose from 100 to 202 mV in a hypertonic solution, indicating osmotic water loss and wilting. D) Effect of water deficit on the RWC time dynamics for a separated leaf from HP. Result depicts that 1 h of drying result in a prominent increase in the V_TRx_ value with the magnitude touching 200 mV, as compared to the 45 min of drying with V_TRx_ value at 180 mV. This trend is indicative of the fact that singular leaves can respond to the change in water availability, even after detachment from the whole plant, which can be effectively captured using the PLP. E) Effect of water deficit on separated leaves of 2 different sizes: big (82 cm^2^) and small (47 cm^2^) from a particular sweet potato plant. In a 12‐h experiment, different dehydration patterns were observed between two leaves. F) Water uptake time signature of dry separated leaves of 2 different sizes: big (81 cm^2^) and small (39 cm^2^) from a particular sweet potato plant. The experiment commenced with a decreased RWC in either of the leaves at a value of 50%. In a 12‐h experiment, different dehydration patterns were observed between two leaves, with the bigger leaf showing 4 drying zones and the smaller showing 2. G) Effect of water deficit on separated leaves of equivalent size (78 and 81 cm^2^) detached from a plant growing in hydroponic conditions versus a plant growing in normal ambience. Both the leaves showed a similar drying slope (18 and 25 mV h^−1^) till 3.2 h, after which the drying curves changed their trajectory significantly (11 mV h^−1^ for hydroponic leaf versus 3 mV h^−1^ for ambient leaf).

This section highlights the proficiency of the PLP in accurately comprehending the RWC of a detached leaf, as well as its ability to monitor hydration levels over time. It's important to note that leaves from various plants exhibit distinct genotypes, which manifest as diverse phenotypes. By affixing the PLP to leaves from different species, we were able to document numerous variations in their responses to various stimuli, including dehydration, rehydration, and osmosis. Such observations are invaluable to plant biologists who are keen on exploring the dynamic behavior of leaves to these stimuli, offering insights that could significantly advance the field of plant biology. To our knowledge, the utilization of wearable technology to procure such insights marks an advancement in plant biological research. PLP was subsequently attached to a separated plant leaf (**Figure** **3**A), which was allowed to dry in a controlled growth chamber. The leaf lost water with a RWC of 46% at the 9^th^ h. The experiment demonstrated PLP's plant wearability on both turgid and wilted phases of the leaf. An effort to compare our proposed PLP device to the conventional lab method of measuring RWC has been made in Note SIII‐A,B (Supporting Information) respectively demonstrating our work's novelty. Before applying the PLP to probe plant tissues, it is important to understand its working principle (see Note SIII‐C, Supporting Information).

The PLP's performance was evaluated on leaves from the dehydration‐sensitive *Hypoestes phyllostachya* (HP or polka dot) and the tolerant *Zamioculcas zamiifolia* (ZZ or raven), showing distinct dehydration responses  (Figure 3B). For the HP leaf, RWC drastically decreased by 98%, with a corresponding 291% V_TRx_ increase. In contrast, the ZZ leaf showed a minor RWC decrease of 6% and a negligible ≈1% V_TRx_ change. The dehydration trend in HP, is nonlinear for both V_TRx_ and RWC mostly due to tissue hardening. The experiment highlighted two key findings: (a) different plant types exhibit varying hydration profiles and (b) the temporal V_TRx_ pattern closely correlates with RWC changes.

In growth chamber tests, the PLP was used to track hydration changes in detached leaves from HP and Ipomoea batatas cv. Beniazuma (JSP), to osmotic dynamics, as shown in Figure 3C. Whereas the JSP leaf did not exhibit any response, the HP leaf demonstrated significant fluctuations. For HP, V_TRx_ rose by 102% in a hypertonic solution, indicating osmotic water loss and wilting. After rehydration, the V_TRx_ continued to increase for 50 min before stabilizing, following a gradual decrease by 11% over 16.5 h, suggesting endosmosis. This delayed recovery in HP might suggest irreversible damage from severe dehydration or hysteresis effects between dehydration and rehydration. The difference in the hydration dynamics between JSP and HP might be due to the difference in the relative sap concentration. JSP having higher sap concentration as compared to the hypertonic solution does not show any variation, unlike HP.

In further experiments with HP, we subjected a detached leaf to two cycles of dehydration and rehydration over 48 h, observing the V_TRx_‐time relationship (Figure 3D). Wilting appeared within an hour of dehydration, lasting 7.5 h before rehydration began, quicker than in hypertonic solution, hinting at impaired rehydration due to increased leaf sap tonicity. As the leaf visibly recovered, the voltage dropped, stabilizing at its initial value, though recovery was slower than dehydration. Interestingly, rehydration continued for 2.75 h post‐dehydration, indicating delayed wilting due to water movement from the petiole to the lamina and delaying the onset of wilting. The second wilting period was shorter, yet recovery was slower and less complete than before, suggesting possible physiological adaptation to drought or irreversible damage from the first dehydration cycle, affecting the leaf's drought response.

In an evapotranspiration water loss test on detached leaves from an SP plant, we observed differing dehydration patterns between two leaves of varied sizes over a 12‐h period (Figure 3E). Intriguingly, the dehydration patterns between the two leaves displayed notable differences. The larger leaf had a higher initial V_TRx_ value and a faster water loss rate of 19.5 mV h^−1^, compared to 9.7 mV h^−1^ for the smaller leaf. This difference is likely due to the larger leaf's greater thickness and porosity. The dehydration response varied with size: the larger leaf showed a linear increase in V_TRx_, followed by a decrease, stagnation, and a final increase. In contrast, the smaller leaf had a more consistent linear increase and then plateaued, highlighting size‐dependent variations in leaf dehydration behavior. PLP was additionally affixed to 3 different leaves from the same SP plant, and differences in the V_TRx_‐RWC relation were observed (see Note SIII‐D, Supporting Information).

In a subsequent test to study rehydration in dehydrated leaves, we monitored changes in relative water content (RWC) over a day (Figure 3F). The rehydration patterns differed notably between the two leaf sizes. The larger leaf showed an initial increase in V_TRx_ for ≈1.7 h (Zone I), a possible lag from drying, followed by a linear decrease over 6.1 h (Zone II), a sharper drop for 3.4 h (Zone III), and then saturating (Zone IV), signaling rehydration completion. Conversely, the smaller leaf displayed simpler dynamics: minimal change for 14.4 h (Zone I), then a gentle decline (Zone II). This experiment demonstrated distinct rehydration behaviors in leaves of different sizes, highlighting the complexity of plant physiological responses under varied hydration conditions.

In our final test, we used PLP to assess RWC in JSP grown in different cultivation formats (Figure 3G), deriving that different growing conditions affect plant physiology. Initially, the dehydration rates of leaves from hydroponically and ambiently grown plants were similar. However, after ≈3.2 h, their dehydration trajectories diverged. The hydroponic leaf's rate slowed to a linear 11 mV h^−1^, while the ambient leaf decreased to ≈3 mV h^−1^ before gradually increasing to match the hydroponic leaf. This variation likely reflects their environmental acclimation, with the hydroponically grown leaf having adaptations like thinner cuticles due to its water‐rich indoor environment. The initial wait time of 3.2 h might represent the hydroponic leaf's reaction time to understand the environmental conditions, after which it starts losing water faster than the ambient leaf, owing to a higher environmental temperature and lower humidity as compared to its native hydroponic environment. Over time, even the ambient leaf, despite its initial drought tolerance, began to dehydrate at a rate similar to the hydroponic leaf, highlighting the impacts of cultivation methods on plant hydration response.

In addition, to the above experiments, it was also observed that plants having different phenotypes have differences in drought response (Note SIII‐E, Supporting Information), thereby strengthening PLP's capability to extract meaningful RWC‐related inferences.

All the detached leaf experiments were carried out in a controlled growth chamber (Note SIII‐F, Supporting Information). Further details about experimental protocols can be found in Experimental Section.

### 1.4 Premise for Application of Machine Learning with PLP

PLP as a standalone device can qualitatively understand and track RWC changes inside a plant leaf through its readout voltage, however, this method alone is insufficient to accurately quantify the value of RWC. This is due to the inexistence of a generalized singular calibration curve which can possibly link the readout voltage to the RWC value. Such a curve could have existed for a linear artificial system; however, leaves being naturally occurring tend to be highly non‐linear, thereby making it impossible to have a generalized singular curve that links readout voltages to the RWC of any universal leaf. This also results in the non‐existence of a singular value for PLP's sensitivity to RWC detection. Thus, it is essential to develop a unique curve generator that can adapt to the specific characteristics of each leaf from every cultivar.

Achieving this level of precision requires the application of generative deep learning models, offering a tailored approach to accurately measure RWC in leaves.

**Figure 4:**
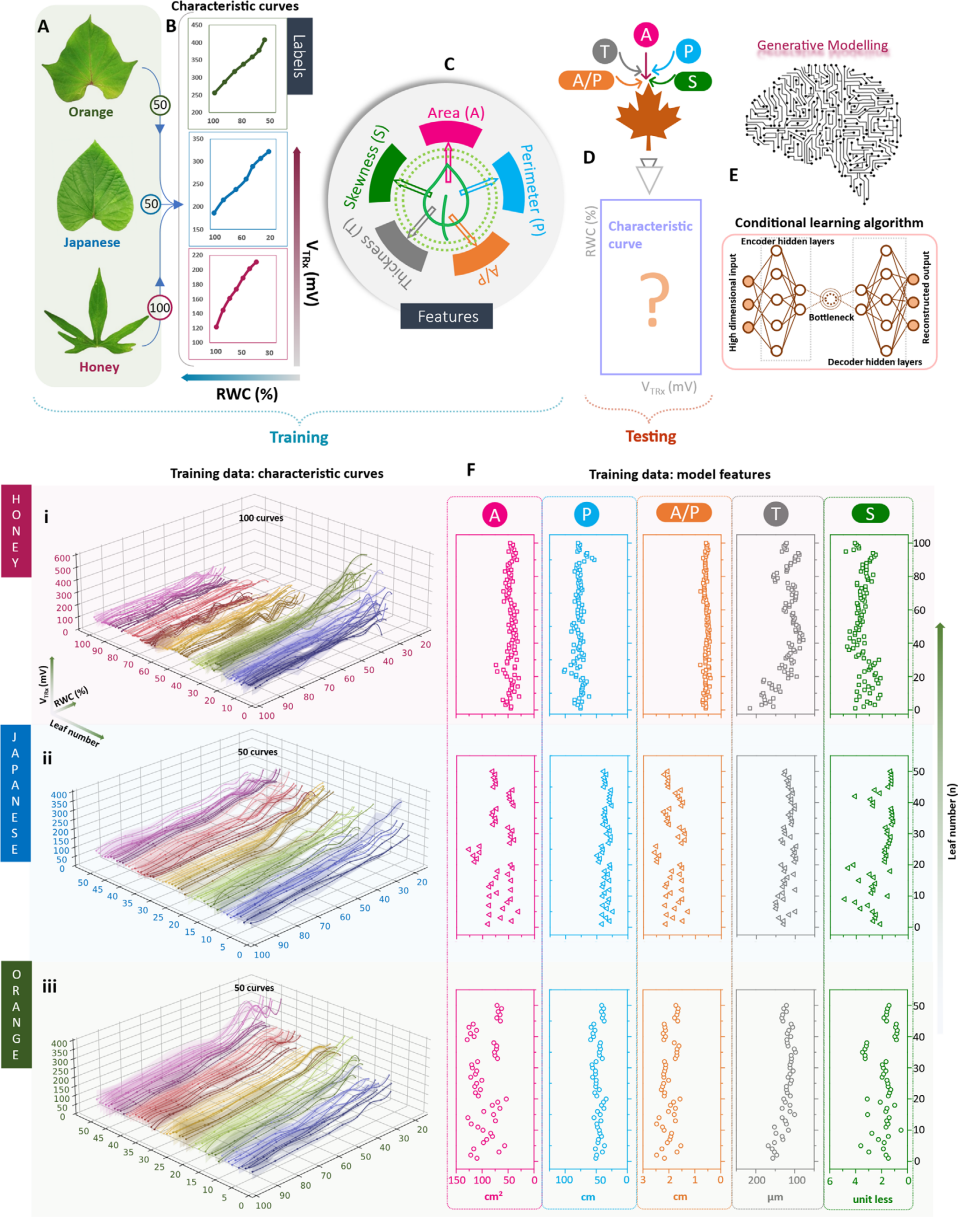
Premise for Application of Machine Learning with PLP. A) Leaves from three sweet potato cultivars were used in the machine learning experiments: OFSP (orange), JSP (Japanese) and Cilembu (honey). B) A cartoon of characteristic curves (V_TRx_ versus RWC) as would be obtained from the leaves, which served as the labels/inputs for the machine learning model; 50 different leaves with 50 different curves, 50 different leaves with 50 different curves, and 100 different leaves with 100 different curves were obtained for orange, Japanese and honey respectively. Figure 4 (continued). Premise for Application of Machine Learning with PLP. C) Machine learning features (such as the area, perimeter, thickness, skewness, and area‐to‐perimeter ratio) to which the curves are related. The area, perimeter and skewness are directly obtained from taking pre‐scaled images of leaf, and then post‐processing the image to extract these geometrical features. Area‐to‐perimeter ratio is next derived from the area and perimeter values. Thickness is measured using the vernier caliper, a mean value is taken a reading D) Prediction of the characteristic curve by the model, given the particular set of features for a particular leaf of interest. E) The task of predicting the characteristic curve is carried out by the conditional variational autoencoder (CVAE) which is a form of generative AI. The structure of CVAE consists of 3 different parts: the encoder neural network, the bottleneck, and the decoder neural network. F) Machine learning training data library, which can be divided into: (a) characteristic curves and (b) model features. The collection of these 2 sets of data together forms the high dimensional input which is fed to the ML algorithm for training purposes.

In this section, we develop such a model using a supervised deep‐learning algorithm. The input for this model includes the characteristic curves of the leaves, linked through their geometrical features. This model is designed to understand the probability distribution that bridges these curves with the specific features of the leaves. Through this process, it learns to act as a bespoke curve generator, tailored to each leaf's unique attributes. To accomplish this leaves from three different sweet potato cultivars were used to build the deep learning model: OFSP (Orange), JSP (Japanese), and Cilembu (Honey). The cultivars are depicted in Note SIV‐A (Supporting Information). Three different cultivars were selected over a single cultivar to diversify the machine‐learning dataset and prove the capability of the AI‐assisted PLP as a diverse RWC sensor (see Experimental Section). The training dataset for the model includes 50 leaves each from OFSP and JSP, and 100 from the Honey cultivar. **Figure** **4**B presents unique schematic characteristic curves for each cultivar, illustrating the inverse relationship between RWC and V_TRx_. These curves are used as the labels/conditions. Each of these curves is unique and is intricately related to the leaves’ geometry such as – area (A), perimeter (P), area‐to‐perimeter ratio (A/P), thickness (T), and skewness (S). Thus, these parameters are used as features in our model (Figure 4C).

The task of predicting the correct characteristic curve based on provided features (Figure 4D) is accomplished using a CVAE and consists of three key components: the encoder hidden neural network, a latent space also known as the bottleneck, and the decoder neural network (Figure 4E). The reason for using CVAE as the deep learning algorithm is delineated in Note SIV‐B (Supporting Information). Figure 4F demonstrates the training data graphically for the different cultivars (see Experimental Section). For each one of the cultivars, the data has been divided into two groups: characteristic curves and model features (see Experimental Section). Features such as the area, perimeter, and skewness are extracted from pre‐scaled leaf photographs (Note SIV‐C, Supporting Information), whereas leaf thickness is measured using a vernier caliper (Note SIV‐D, Supporting Information) In the characteristic curves, for Honey, the V_TRx_ and RWC values varied by 91% and 79% respectively (see Figure 4Fi). For JSP, the V_TRx_ and RWC values varied by 75% and 81% respectively (see Figure 4Fii). For OFSP, the V_TRx_ and RWC values varied by 77% and 84% respectively (see Figure 4Fiii). Although the variation in the RWC was nearly identical for the cultivars, V_TRx_ showed a difference for the Honey cultivar in contrast to JSP and OFSP. For Honey, two clear V_TRx_ groups exist: group A having a maximum range of 300 mV, and group B has a maximum range of 600 mV. Although there is not a clear explanation for the reason behind such an observation, the values depict the possibility of the existence of genotypical variation in different plants from the same cultivar of sweet potato. In the model features, for Honey, the A, P, A/P, T, and S values varied by 155, 91, 100, 173, and 125% respectively (see Figure 4Fi). For JSP, the A, P, A/P, T, and S values varied by 302, 102, 158, 54, and 326% respectively (see Figure 4Fii). For OFSP, the A, P, A/P, T, and S values varied by 141, 73, 60, 72, and 620% respectively (see Figure 4Fii). Although a direct correlation between the features and the characteristic curves is difficult to visualize, it is strongly believed that such differences will be apparent in the machine learning model, thereby strengthening the need to use the AI‐assistance. The look of a sample dataset that contains the curve‐feature information is depicted in Note SIV‐E (Supporting Information). Model training curves are re‐depicted from a different angle for clarity (Note SIV‐F, Supporting Information)

### 1.5 Generative Modeling Predictions and RWC Estimation

**Figure 5:**
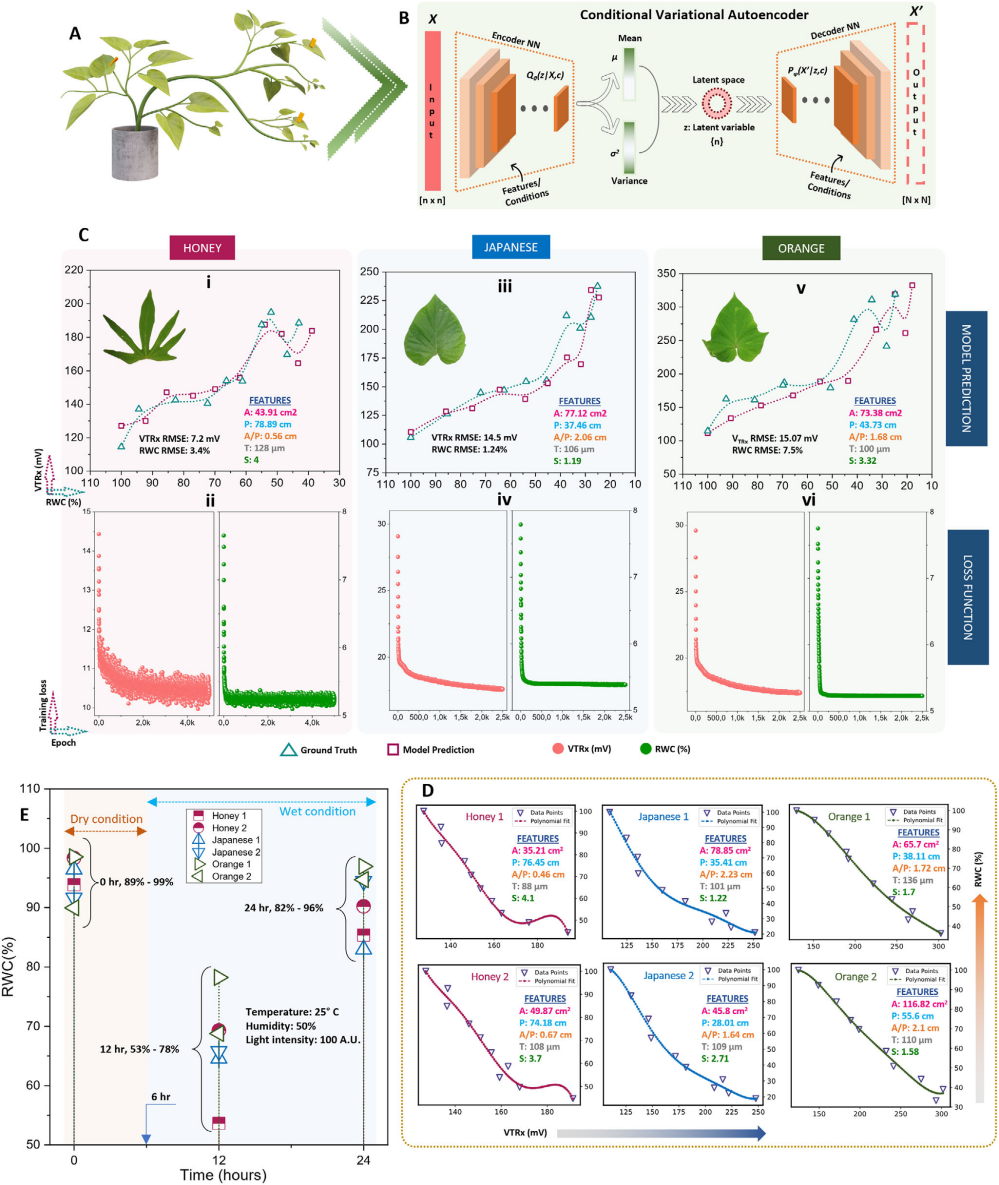
Generative modeling prediction and RWC estimation. A) A 3D schematic of the JSP sweet potato plant with PLP attached to various leaves. B) The structure of the generative AI: conditional variational autoencoder model used for generating the characteristic curves. Data in the form of n x n array is provided as the model input and first fed into the encoder neural network consisting of hidden layers. Features are inputted separately as conditions in the model. The model then learns the governing probability distribution elements such as the mean and variance which is passed on to the lower dimensional latent space. The output from the latent space is then fed into the decoder neural network containing hidden layers which finally outputs a particular curve, given the set of features. C) Model prediction versus ground truth as observed in leaves from three different sweet potato plant cultivars, with V_TRx_ root mean square error (RMSE) and RWC RMSE being 7.2 mV and 3.4% for honey, 14.5 mV and 1.24% for Japanese, and 15.07 mV and 7.5% for the orange cultivars respectively. Loss functions for each of the predictions depict an exponential decay in loss and thereby attaining saturation in the prediction capability, over 20 000 epochs for honey, 1000 epochs for Japanese and orange respectively. Figure 5 (continued). Generative modeling prediction and RWC estimation. D) Whole plant on‐shot RWC measurement: Characteristic curves as obtained from a pair of leaves belonging to three different sweet potato cultivars using the generative model. Each such characteristic curve is specific to the particular set of geometric features pertaining to the leaves. These curves will thus transfer the V_TRx_ obtained from the device upon attachment to the leaves and to the corresponding RWC values. E) One‐shot time dynamic PLP reading from the leaf pair belonging to three different cultivars when exposed to water stress. The leaves to which the characteristic curves were generated were then dynamically attached with the PLP which along with the generated curves were able to measure the RWC value from each leaf. The experiment started with dry conditions till the first 6 h, after which the plants were irrigated. One‐shot readings were taken at the 0th, 12th, and 24th h respectively. At 0th h, the RWC values as recorded by the PLP was in the range of 89 to 99% representing the initial turgid condition. At 12th h, the RWC values were in the range of 53–78%, which was seemingly lower than the initial RWC range, suggesting the effect of drought on the plants. At 24th h, the RWC range increased to 82% to 96%, suggesting moisture absorption and re‐attainment of turgidity.

This section elucidates the efficacy of our proposed model to predict the unique leaf characteristic curve, based on the provided geometrical features. Following the model's development, we further enhance its capabilities by integrating V_TRx_ along with the generative AI‐assistance to quantify RWC dynamically via using the re‐attachable feature of the PLP. Consequently, we have been able to showcase the PLP as an effective real‐time, one‐shot RWC detector when applied to leaves from whole plants of three distinct SP cultivars. To our knowledge, such an application has not been previously documented and represents a novel method for assessing plant health outdoors with just a single touch on any leaf. **Figure** **5**A features a 3D picture of a typical JSP plant with the PLP attached to leaves of varying features. The working architecture of a CVAE algorithm is pictorially represented in Figure 5B with more detailed information in Note SIV‐G (Supporting Information) (see Experimental Section). The CVAE algorithm was trained for 20 000 epochs, 10 000 epochs, and 10 000 epochs for the honey, Japanese, and orange cultivars with predictions being demonstrated in Figure 5C. Honey, JSP, and OFSP cultivars were tested for 10, 5, and 5 different leaves bearing different characteristic curves and the best result obtained for each cultivar is depicted in Figure 5C i,iii,v.

The root mean squared error (RMSE) was utilized to identify the most accurate characteristic curves for different sweet potato cultivars. For Honey, the RMSE for V_TRx_ and RWC was 7.2 mV and 3.4%, respectively, with the loss function reducing from 14.42 mV^2^ and 7.64%^2^ to 9.96 mV^2^ and 5.09%^2^ (Figure 5Ci, 5Cii). The JSP showed a V_TRx_ RMSE of 14.5 mV and an RWC RMSE of 1.24%, with the loss function declining from 29.04 mV^2^ and 7.78%^2^ to 17.58 mV^2^ and 5.38%^2^ (Figure 5Ciii, 5Civ). For OFSP, the RMSE values for V_TRx_ and RWC were 15.07 mV and 7.5%, respectively, and the loss function decreased from 29.62 mV^2^ and 7.76%^2^ to 17.39 mV^2^ and 5.22%^2^ (Figure 5Cv,Cvi). As can be observed, the model‐predicted characteristic curve matched closely with the ground truth, with slight differences attributed to the number of leaf samples for which the model was developed. The use of 200 leaves to train the model might be slightly insufficient for a generative AI model, and an increase in the number to 500 might prove further useful (Note SIV‐H, Supporting Information).

Next, generative AI‐assisted PLP was used as a one‐touch RWC sensor. Three JSP plants from different cultivars were chosen. Two leaves per cultivar were randomly selected, their features extracted, and fed into the model to predict their characteristic curves. Figure 5D displays the six distinct characteristic curves corresponding to the diverse feature sets of the six leaves. Figure 5E illustrates the PLP's performance on whole plants of three different sweet potato cultivars (see Experimental Section) which were subjected to 6 h of environmental drying followed by 18 h of irrigation. RWC values measured by PLP varied from 89 to 99% initially, and dropped from 53 to 78% at the 12th h, due to the first 6 h of drying. This was followed by an increase from 82 to 96% at the 24th h. This experiment thereby confirms the capability of the AI‐assisted PLP plant wearable as a one‐touch RWC sensor and suggests the applicability of such a device in detecting RWC from a diverse range of plants. Such a system can be used by plant biologists to measure the RWC of various leaves in their plants in a single shot without the need for leaf detachment, thus not only facilitating fast RWC detection (reducing the detection time by 99.8% over the conventional lab technique) but also making the RWC detection free of usage of bulkier equipment, thereby making it energy efficient.

### 1.6 Whole Plant Dehydration‐Irrigation Time Dynamics with PLP

**Figure 6:**
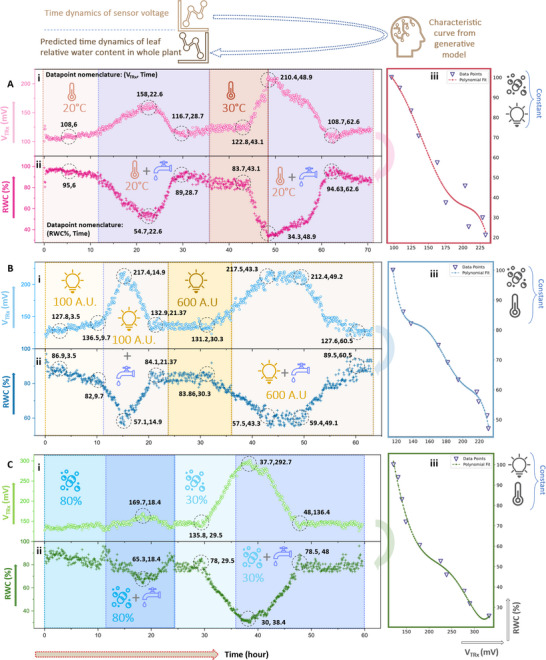
Whole Plant Dehydration‐Irrigation Time Dynamics with PLP. The V_TRx_ values obtained from the PLP are converted to RWC value via the characteristic curve generated by the generative AI model. The PLP was attached to 3 different leaves from 3 different whole plants of the Japanese sweet potato. All the 3 leaves had different geometric features. The plants were then exposed to variation of temperature, light, and humidity with variation in the levels of irrigation and PLP attached to the specified leaf was used to map/monitor the plant's health by using RWC as the biomarker. A) The effect of the duo of temperature and irrigation on RWC time dynamics: i. V_TRx_ versus time as obtained from the PLP ii. RWC versus time as obtained from the PLP + generative AI iii. Characteristic curve as predicted using generative AI. B) The effect of the duo of light intensity and irrigation on RWC time dynamics: i. V_TRx_ versus time as obtained from the PLP ii. RWC versus time as obtained from the PLP + generative AI iii. Characteristic curve as predicted using generative AI. C) The effect of the duo of humidity and irrigation on RWC time dynamics: i. V_TRx_ versus time as obtained from the PLP ii. RWC versus time as obtained from the PLP + generative AI iii. Characteristic curve as predicted using generative AI. Because the PLP can remain attached for multiple days without recalibration, the sensor captures slow diurnal and multi‐day hydration trends that conventional spot‐measurement tools miss. These time‐resolved data form the basis for predictive irrigation scheduling and early drought‐stress warnings at the leaf level.

The final stage of our study involved evaluating the generative AI‐assisted PLP device for measuring leaf RWC on whole plants (Experimental Section). The experimental setup is depicted in Note SIV‐I (Supporting Information). Three different leaves having different features on three different JSP whole plants were selected, and their respective characteristic curve were extracted from the ML model upon providing the respective features. The characteristic curve transfers the V_TRx_ values from PLP to the respective RWC values. The combined effects of environmental variables such as temperature, light intensity, humidity, and controlled irrigation on the RWC time dynamics is thus observed in this study. **Figures** **6**Ai,ii and 6Bi,ii and 6Ci,ii present the time dynamics of V_TRx_ and RWC, while Figures 6Aiii and 6Biii and 6Ciii showcase the V_TRx_ to RWC transfer function (characteristic curve), as determined by the model.

Figure 6A shows the effect of temperature and irrigation on the plant's RWC dynamics (see Experimental Section). Over a period from 6 to 22.6 h, at 20 °C, RWC decreased from 95% and stabilized at ≈54.7% for 3 h, then recovered to ≈89% by 28.7 h. At 30 °C, RWC began falling at 43.1 h, reaching a minimum of 34.3%, significantly lower than at 20 °C. Notably, the plant's response to rehydration was faster after the second dehydration phase, despite more stressful conditions and lower RWC, contrasting with the delayed rehydration observed in detached leaf tests (Figure 3D). Furthermore, this immediate response was not observed in the subsequent trials, further suggesting that temperature may be a much more impactful stressor from the plant's perspective. This far more rapid response to rehydration may be instigated by the rest of the plant, rather than solely the leaf, and could be the outcome of physiological adaptation to the previous dehydration state or to growth chamber conditions.

The effect of light intensity and irrigation on the plant's RWC dynamics is depicted in Figure 6B (see Experimental Section). Initially, at 100 A.U. of light intensity, RWC fell from 86.9% to 82% between 3.5 and 9.7 h, then reversed direction, stabilizing at 84.1% by 21.37 h. In the next irrigation cycle with increased light intensity (600 A.U.), RWC dropped from 83.86 to 57.5% between 30.3 and 43.3 h, then began recovering from 49.1 h, reaching a final RWC of 89.5%. Notably, there was a delayed dehydration response during the second irrigation cycle, with rehydration occurring less abruptly compared to the scenario with increased ambient temperature. The delayed dehydration and rehydration in combination with prolonged temperature. The delayed dehydration and rehydration in combination with prolonged saturation might be the result of the plant's short‐term adaptation to the environment.

The effect of humidity and irrigation on the plant's RWC dynamics is shown in Figure 6C (see Experimental Section). In the initial high humidity condition, the plant's RWC changed minimally, reaching 65.3% at 18.4 h, as high humidity reduces water loss through evapotranspiration. During this period, minimal fluctuation in leaf RWC was noted, but the roots appeared visibly dry, indicating less desiccation tolerance. In the subsequent cycle under low humidity conditions, a more significant RWC decrease was observed, dropping from 78% to 30% between 29.5 and 38.4 h, followed by a rapid recovery to 78.5% by 48 h. The uninterrupted three‐day trace demonstrates that PLP‐derived RWC dynamics track ambient temperature and humidity cycles with sub‐hour resolution, enabling data‐driven irrigation decisions that can reduce water use by an estimated 15–20% compared with fixed‐schedule watering. Such long‐horizon, high‐density datasets are otherwise unattainable with destructive or manually repositioned probes.

In all, these final series of whole plant trials demonstrate the capability of the AI‐assisted PLP device for studying plant water dynamics at a precise temporal resolution. Key features of the plants’ responses to irrigation cycles were clearly distinguishable within the same plant as well as between different environment regimes. Despite the representative leaves in each trial exhibiting different magnitudes of V_TRx_, the mapped RWC values were comparable, further validating the robustness of our AI‐based RWC sensor.

## 2 Conclusion

We developed a non‐invasive plant‐wearable sensor (PLP) that combines CMOS‐compatible piezo‐MEMS ultrasound with generative AI to measure leaf RWC in real‐time. Unlike traditional destructive methods, this device delivers durable, accurate hydration readings for both one‐time and continuous monitoring. Its indefinitely re‐attachable design enables repeated measurements across multiple leaves without any performance loss, making it more practical and versatile than conventional disposable e‐skin plant sensors.

As an initial step in this study, we fabricate the PMUT die, which serves as the fundamental building block of the PLP, using the novel PSON fabrication process. This process is followed by a detailed vibroacoustic characterization to evaluate the performance and reliability of the PMUT under various conditions. Subsequently, we carry out a series of iterative optimization trials to determine the most effective strategy for packaging the PMUT die into the PLP. During this stage, we emphasize critical operational traits such as the re‐attachability of the device and its non‐invasive interaction with plant leaves, ensuring it aligns with the long‐term usability goals of the sensor. To further validate the PLP, we demonstrate its capability as a hydration sensor by testing it in porous fabrics designed to mimic the intricate structural and physiological properties of biological tissues, such as plant leaves. Through this approach, the PLP provides insights into the non‐linear drying dynamics of porous materials, a process that is otherwise difficult to quantify.

Additionally, by attaching the PLP to detached leaves, we open new avenues for analyzing key biological processes, including transpiration, water absorption, and osmotic movement. These measurements are performed across leaves of varying ages, enabling a deeper understanding of plant hydration behavior over time. To make the PLP a versatile RWC sensor capable of accommodating the natural diversity of leaves, we incorporate advanced generative AI models, specifically autoencoders. These models analyze electrical signals generated by the PLP, along with visual leaf traits, to create highly accurate, leaf‐specific voltage‐to‐RWC conversion functions. Using 200 input curves for training, the AI achieves a minimal root mean square error (RMSE) of 6.7% for voltage predictions and 1.55% for RWC estimations.

Finally, deploying the PLP across the foliage of a plant grown in a controlled growth chamber allows for continuous, real‐time monitoring of hydration levels. This setup provides important data on how plants respond to various environmental stimuli, such as light, temperature, humidity, and irrigation practices. By unraveling these dynamics, the PLP significantly advances our understanding of plant physiology and offers a powerful tool for agricultural applications, including optimizing irrigation strategies and improving crop resilience under diverse environmental conditions.

Compared to traditional ultrasound systems, our technology offers significant advantages such as lower power consumption, lighter weight, increased portability, greater miniaturization, and improved plant compatibility. These improvements mark a major advancement in using ultrasound for monitoring plant dehydration, which is a vital marker for detecting and managing plant responses to environmental stress and disease. This innovation paves the way for future developments, such as further miniaturization through CMOS‐PMUT monolithic integration, the incorporation of the Internet of Things (IoT) enabling global mobile connectivity, the integration of edge/in‐sensor computing for faster AI‐enabled response, and the combination of plant sensor area networks with digital twin, enabling multi‐plant digital health monitoring.^[^ ^122^ ^]^ Thus, this integration of thin‐film piezo‐MEMS ultrasound, generative AI, and direct plant‐leaf engagement paves the way for more informed management of water‐related influences on agriculture productivity such as in developing drought and waterlogging coping measures. The prospective influence on biologists to study plant phenotypes using RWC as the biomarker and on agriculturists to determine the efficiency of agricultural outputs is profound, signaling the dawn of an advanced phase in intelligent plant care.

## 3 Experimental Section

### PSON ScAlN PMUT Fabrication

The fabrication process begins with an array of holes patterned and etched into a bulk silicon wafer as shown in Figure S4i (Supporting Information). These holes were then annealed in a hydrogen ambient at high temperature, and low pressure to form the silicon‐on‐nothing (SON) structure with a 2 µm silicon membrane over an ≈1 µm deep cavity as shown in Figure S4ii (Supporting Information). The silicon was then locally diffusion doped (n++) using a silicon oxide mask, forming the bottom electrode as shown in Figure S4iii (Supporting Information). Next, 0.3 µm Sc_0.15_Al_0.85_N was deposited and patterned for the piezoelectric transduction layer as shown in Figure S4iv (Supporting Information). Then, 0.2 µm Mo was deposited and patterned to define the top electrode. The Mo top electrode was then passivated with an additional 50 nm ScAlN to avoid subsequent oxidation of the Mo electrodes as shown in Figure S4v (Supporting Information). Thereafter, a 2 µm silicon oxide layer was pattern deposited for insulation as shown in Figure S4vi (Supporting Information). The silicon oxide was next patterned thereby following a reactive ion etch to define vias to connect the Molybdenum layer. This was followed by patterned deposition of the Aluminum layer to define the bond pads as shown in Figure S4vii (Supporting Information).

### PLP Fabrication

The PLP fabrication process starts with designing and manufacturing the flexible PCB to which SMA connector is first soldered followed by the bonding of the PMUT die using the H70 E non‐conductive thermally insulating epoxy from EpoTek Inc (Figure S8i, Supporting Information). The perimeter of the die is next secured and levelled following a gradual slope using the Crystal 2K epoxy from Araldite Inc. (Figure S8ii, Supporting Information). The die is then wire bonded to the PCB for effective connection (Figure S8iii, Supporting Information). The naked wirebonds are next protected using the Crystal 2K epoxy thereby strengthening the contacts (Figure S8iv, Supporting Information). The spacer containing a coin magnet is next 3D printed and carefully bonded to the flex PCB (Figure S8v, Supporting Information). The naked end of the solder is next levelled using the clear epoxy. Next, the flex PCBs are visually aligned, and the epoxy insulated part fused together to form the PLP clip (Figure S8vi, Supporting Information).

### Textile Experiments Protocols

For the characteristic curve experiment (Figure 2G): The transmitter PMUT was operated at 5 V AC for this experiment. The pieces of textiles were cut in the dimension of 3 cm x 2 cm and submerged in water in a container for 5 min, ensuring its wet. After removal, each piece are waved 20 times to drip off excess water. Mass and the voltage were then measured using the mass balance and the PLP respectively. The pieces were put in the muffled oven for 2 min at 50 °C. The process was repeated 10 times.

For the real time temperature experiment (Figure 2H): Each textile was first cut into small pieces of 3 cm x 2 cm and submerged in water for 1 min. After wetting in water, the pieces were suspended in the natural ambience for a few min to drop any excess water. The transmitter PMUT was driven at 5 V AC.

### PLP on Detached Leaves Protocols

General: Any plants used were hydrated for 12 h before the commencement of experiment to ensure maximum turgor. Leaves detached from those plants were additionally hydrated for 12 h water to offset any wilting caused due to the detachment.

For the water loss dynamics of separated leaves from drought sensitive and insensitive plants (Figure 3B): The temperature maintained was 50 °C, at a relative humidity of 50%. RWC and V_TRx_ measurements were taken after every 2 min.

For Osmotic time dynamics as observed form a Polka Dot and a sweet potato separated leaf (Figure 3C): Fully hydrated, detached leaves fitted of comparable size from each species were fitted with the PLP and then immersed for 2 h by submerging the petioles in water, followed by immersing in a saturated sugar solution for 5.7 h and then replacing the leaves in water for another 17.5 h. To reduce the confounding effects from evapotranspiration, each leaf was uniformly coated with petroleum jelly.

For Effect of water deficit on separated leaves of different sizes from a particular sweet potato plant: Two distinct leaf units were selected: (a) a larger leaf spanning 82 cm^2^ and (b) a smaller counterpart covering 47 cm^2^. For the experiment, they were placed in a controlled setting with no water, at a temperature of 35 °C and 35% relative humidity (RH) of light to ensure uniform growth conditions. This setup was meticulously maintained in a growth chamber to mitigate any minor environmental fluctuations (Figure 3E).

Water uptake time signature of dry separated leaves of different sizes from a particular sweet potato plant: Proceeding onward to investigating rehydration dynamics from a dehydrated state, a second test was carried out with two different‐sized leaves measuring 81 and 39 cm^2^. Following this, they underwent oven drying at 40 °C – the larger leaf for 35 min and its smaller counterpart for 20 min–to achieve an ≈50% RWC. This specific drying duration was deduced from preliminary trials. The test commenced after submerging the petioles of both leaf units in water, with environmental conditions meticulously maintained at 25 °C, and 35% RH (Figure 3F).

Effect of water deficit on separated leaves of equivalent size detached from a plant growing in hydroponic conditions versus a plant growing in normal ambience: One leaf was sourced from a plant in potted soil grown outdoors, while another of comparable size was sourced from a plant in potted soil grown in an indoor hydroponic environment (Figure 3G).

### Premise for Application of Machine Learning

Three different cultivars of the sweet potato plant such as: the Orange (orange fleshed sweet potato: OFSP), Honey (Cilembu) and Japanese (Beniazuma) were selected as the test plants for which the RWC values were to be determined by using the PLP (Figure 4A). As is visually evident, the cultivars demonstrate striking differences in their shapes and can be classified by geometrical quantifiers. The reason for selecting three distinct cultivars is to generate a level of diversity within the dataset, which would improve the robustness of our ML approach. A greater number of leaves were selected to cater to the higher population density of leaves in a similar sized plant belonging to the honey cultivar as compared with the other cultivars all having diverse geometrical features.

As described in the concept abstraction, the purpose of using deep learning in this contribution is to predict characteristic curves for unseen leaves, based on the learning data (the labels and the features), thereby achieving a generality in relative water content prediction in plants. We aim at achieving this by using generative deep learning algorithms such as the conditional variational autoencoder (CVAE) which is comprised of three different hidden functional blocks such as the encoder hidden neural network, a latent space also known as the bottleneck and the decoder neural network as shown in Figure 4E.

Characteristic curves and features (Figure 4F): The high dimensional input is the array of the (x,y) ordered pairs of datapoints making up each characteristic curve multiplied by the number of total curves. Thus, the input matrix is 100 x 10, 50 x 10 and 50 x 10 for honey, Japanese and orange sweet potato cultivars respectively. The reconstructed output corresponds to the characteristic curve that the learnt model will produce once a known/unknown feature set is presented to the model. Three different master models were used in this contribution for three different cultivars to achieve better predictability, with each model having 2 slave models for predicting the voltage and the water content separately.

The characteristic curves have been represented in a stacked 2D plot depicting a 3D visualization. Each 2D plot has two axes: V_TRx_ in mV and RWC in %, stacked over the total number of curves used to train the model. Obtaining such a curve follows the following experimental protocol: The plant from which the leaves are separated is pre‐watered for 24 h thereby ensuring fully turgid conditions. Next, visually healthy leaves are selected and detached from the plant in a set of six. Each leaf is placed on a white sheet of paper having scaled ruler graduations, and then a photograph is captured for post processing to extract the features (geometric quantifiers) as described in Figure S23 (Supporting Information). Leaf thickness is measured separately using a vernier caliper (Mitutoyo Inc.) in leaf blade regions devoid of veins, to ensure uniformity in results. Next, the leaves are measured and recorded for their masses using a mass balance and the voltage across the leaf blades (V_TRx_) at various positions are determined by using the PLP and recorded. V_TRx_ has been recorded at two regions for the honey cultivar and four regions for the Japanese and orange cultivars respectively. The positions of the vernier and PLP placement in leaves from various cultivars has been depicted in Figure S24 (Supporting Information). Next, the set of leaves are heated at 60 °C in a muffled furnace for two and a half minutes followed by the recording of the leaf mass and the V_TRx_. This process is looped 10 times in total to obtain the entire V_TRx_ versus leaf mass curve. The leaves are then kept for drying over a period of 24 h at the same temperature to obtain the dried mass thereby enabling the calculation for the RWC for each reading in the dataset. Each of the 5 features are graphed as 2D scatter plot with the y axis representing the leaf number and the x axis representing the respective feature magnitudes in cm^2^, cm, cm, µm and unit less for area, perimeter, area‐to‐perimeter ratio, thickness, and skewness respectively.

### Predictions of CVAE: The Generative Algorithm

A CVAE is an extension of VAE (variational autoencoder) which is a class of generative models that leverage principles from variational inference to enable the learning of complex probability distributions. It is characterized by its ability to encode input data into a compressed latent space with an imposed probabilistic structure and subsequently generate new data by sampling from this space. Figure 5B represents the architectural construct of the model. A more detailed description is also available in Figure S27 (Supporting Information).

The high dimensional input which in this case is the set of labeled characteristic curves after being concatenated with the features/conditions is fed to the algorithm as the net input, which first enters the encoder neural network (NN). The encoder NN is associated with mapping the data to a lower dimensional representation by processing the data through several fully connected layers. The output of the encoder NN is the parameters of a probability distribution for the latent variables which are in essence hidden or unobserved variables that are probabilistic in nature and are often associated with a Gaussian distribution. The Gaussian distribution will be characterized by probabilistic parameters that the encoder NN will produce such as the mean (*µ*) and the variance (*σ^2^*). The number dependent variables depend on the dimension of the latent space which is a design choice and is set while making the algorithm architecture. As shown in the figure, for the input X, the encoder NN will output statistical parameters such as the *µ* and the *σ^2^* defining a probability distribution *Q_θ_(z|X,c)* in the latent space. Here, *z* represents the latent variables, *θ* denotes the parameters of the encoder network and c stands for the conditional input in terms of features. Subsequently, the architecture will have a decoder NN which takes samples from the latent space and attempts to reconstruct the original input data. However, like the encoder, the decoder is also probabilistic. Instead of mapping the latent representation directly to an output, it maps it to a distribution over the possible outputs. The decoder NN defines a probability distribution *P_φ_(X’|z,c)* over the original data space where X’ is the reconstructed data and are the parameters of the decoder NN. During the reconstruction/training phase, the decoder NN generates data by sampling from *P_φ_(X’|z,c)*. In the generative phase (after the model has been trained has been trained), new data can be generated by first sampling a latent vector *z* from the prior distribution and then passing it through the decoder NN to get *P_φ_(X’|z,c)*.

As both the V_TRx_ loss and the RWC training losses are minimized, and the loss function assumes steady state after a rapid descent, following a hyperbolic decay, the training is assumed to be complete, and model's efficacy to prediction is tested.

24‐h whole plant one‐shot PLP attaching experiment (Figure 5E): The roots of three plants were removed from the soil and placed in a beaker full of water for 12 h span of time, after which the water was removed, and the plants were exposed to drying for the next 6 h. The point of time when the water was removed can be considered as the starting point for the experiment corresponding to the 0th h. The experiment took place in the growth chamber having maintained temperature of 25 °C, Humidity of 50% and light intensity of 100 A.U. A 24‐h experiment was next conducted for these six different leaves as attached to the whole plant to test the capability and versatility of the PLP to detect dynamic changes in water concentration in three different cultivars. For each of the six leaves, the PLP was attached to obtain the RWC values at the 0th, 12th, and 24th h respectively.

### Whole Plant Dehydration‐Irrigation Time Dynamics with PLP

The complete deep learning enabled PLP sensing platform's performance for measuring leaf RWC on intact plants was investigated as the final stage of this contribution. The experimental setup as placed inside the growth chamber is depicted in Figure S29 (Supporting Information). Using JSP cultivar plants, three different trials were conducted within the growth chamber, with temperature, light, and humidity being the environmental variables of interest, with the goal being to demonstrate the PLP platform's performance for monitoring RWC under different environmental parameters. This in conjunction with a micro irrigation scheme with time dependent watering capability was used to create short periods of water availability and non‐availability, thereby creating an environment of time variant water stress. The micro irrigation setup was created by implementing the usage of two peristaltic pumps with a flow rate of 100 mL min^−1^ which could water and dewater the plant via a storage reservoir. Whole JSP plants were placed bare root into glass jars; soil was omitted to eliminate nonlinear time latencies from its water buffering effect. For these trials, leaf features were first collated and fed to the deep learning model prior to being affixed with the PLP sensor to predict the output characteristic graph containing 10 data points. From this, the continuous dependency of the RWC on V_TRx_ could be obtained by fitting with a polynomial function, which was subsequently used to translate values from voltage to the RWC domain.

Effect of temperature plus irrigation on plant RWC dynamics (Figure 6A): Using the PLP platform, the response of leaf RWC to ambient temperature differences while under dynamic irrigation was assessed. The whole plant was subjected to two cycles of 12 h of dehydration followed by 24 h of rehydration. The ambient temperature was set to 20 °C throughout the trial except during the second dehydration phase, during which was set to 30 °C. Light intensity and relative humidity were kept at 100 A.U. and 30% respectively.

Effect of light intensity plus irrigation on plant RWC dynamics (Figure 6B): In the second trial, the RWC response to changes in ambient lighting environment together with irrigation cycling was examined using the PLP. As before, a whole plant was transplanted to a dry jar and subjected to the following irrigation and light intensity regime: 12‐h dehydration and 12‐h rehydration at 100 A.U. of light intensity, followed by 12‐h dehydration and 36‐h rehydration at 600 A.U. of light intensity. The temperature and relative humidity were kept at 25 °C and 30% respectively.

Effect of humidity plus irrigation on plant RWC dynamics (Figure 6C): In the third trial, the RWC response to changes in relative humidity and irrigation cycling was examined. Once more, a whole plant was transplanted to a dry jar and subjected to the following humidity regime: 12‐h dehydration and 12‐h rehydration at 80% relative humidity, followed by 12‐h dehydration and 36‐h rehydration at 30% relative humidity. The lighting and the temperature levels were kept constant at 100 A.U. and 25 °C throughout.

## Conflict of Interest

The authors declare no conflict of interest.

## Author Contributions

K.R. and C.L. conceived the project. K.R designed the general flow of the manuscript, designed, and conducted all experiments, established the premise of using machine learning in conjunction with PMUTs, created all the figures and drafted the manuscript. D.S. and S.S. advised on plant biological phenologies, provided fresh sweet potato plants used in the project. S.S. also provided access to the growth chamber used for conducting experiments. L.W. coded the generative AI algorithm. Z.Z. determined the specific deep learning model to be used in this project and supervised the work of L.W. Z.Y. and her team developed PSON PMUTs. K.R., D.S., X.G., Z.Y., S.S., and C.L. advised on the writing of the manuscript. C.L., S.S., and Z.Y. wrote successful grants which supported this project.

## Supporting information



Supporting Information

## Data Availability

The data that support the findings of this study are available from the corresponding author upon reasonable request.

## References

[1] A. Singh , B. Ganapathysubramanian , A. K. Singh , S. Sarkar , Trends Plant Sci. 2016, 21, 110. 26651918 10.1016/j.tplants.2015.10.015

[2] M. M. Tahat , K. M. Alananbeh , Y. A. Othman , D. I. Leskovar , Sustainability 2020, 12, 4859.

[3] O. Calicioglu , A. Flammini , S. Bracco , L. Bellù , R. Sims , Sustainability 2019, 11, 222.

[4] K. Al‐Kodmany , Buildings 2018, 8, 24.

[5] C.‐C. Qu , X.‐Y. Sun , W.‐X. Sun , L.‐X. Cao , X.‐Q. Wang , Z.‐Z. He , Small 2021, 17, 2104482. 10.1002/smll.202104482 34796649

[6] H. Malik , N. Fatema , J. A. Alzubi , AI and Machine Learning Paradigms for Health Monitoring System: Intelligent Data Analytics, ol. 86. Springer Nature, Berlin, 2021.

[7] S. Madakam , V. Lake , V. Lake , V. Lake , J. Comput. Commun. 2015, 3, 164.

[8] F. Shah , W. Wu , Sustainability 2019, 11, 1485.

[9] J. S. Duhan , R. Kumar , N. Kumar , P. Kaur , K. Nehra , S. Duhan , Biotechnol. Reports 2017, 15, 11. 10.1016/j.btre.2017.03.002 PMC5454086 28603692

[10] H. Yin , Y. Cao , B. Marelli , X. Zeng , A. J. Mason , C. Cao , Adv. Mater. 2021, 33, 2007764. 10.1002/adma.202007764 33829545

[11] Y. Luo , M. R. Abidian , J.‐H. Ahn , D. Akinwande , A. M. Andrews , M. Antonietti , Z. Bao , M. Berggren , C. A. Berkey , C. J. Bettinger , J. Chen , P. Chen , W. Cheng , X. Cheng , S.‐J. Choi , A. Chortos , C. Dagdeviren , R. H. Dauskardt , C.‐A. Di , M. D. Dickey , X. Duan , A. Facchetti , Z. Fan , Y. Fang , J. Feng , X. Feng , H. Gao , W. Gao , X. Gong , C. F. Guo , et al., ACS Nano 2023, 17, 5211. 36892156 10.1021/acsnano.2c12606 PMC11223676

[12] S. P. Lee , G. Ha , D. E. Wright , Y. Ma , E. Sen‐Gupta , N. R. Haubrich , P. C. Branche , W. Li , G. L. Huppert , M. Johnson , H. B. Mutlu , K. Li , N. Sheth , J. A. Wright , Y. Huang , M. Mansour , J. A. Rogers , R. Ghaffari , NPJ Digit. Med. 2018, 1, 2. 31304288 10.1038/s41746-017-0009-x PMC6550217

[13] D. Franklin , A. Tzavelis , J. Y. Lee , H. U. Chung , J. Trueb , H. Arafa , S. S. Kwak , I. Huang , Y. Liu , M. Rathod , J. Wu , H. Liu , C. Wu , J. A. Pandit , F. S. Ahmad , P. M. McCarthy , J. A. Rogers , Nat. Biomed. Eng. 2023, 7, 1229. 37783757 10.1038/s41551-023-01098-y PMC10653655

[14] Y. Park , H. Luan , K. Kwon , T. S. Chung , S. Oh , J.‐Y. Yoo , G. Chung , J. Kim , S. Kim , S. S. Kwak , J. Choi , H.‐P. Phan , S. Yoo , H. Jeong , J. Shin , S. M. Won , H.‐J. Yoon , Y. H. Jung , J. A. Rogers , npj Flex. Electron. 2024, 8, 6.

[15] S. Chen , J. Qi , S. Fan , Z. Qiao , J. C. Yeo , C. T. Lim , Adv. Healthcare Mater. 2021, 10, 2100116. 10.1002/adhm.202100116 33960133

[16] Q. Li , L.‐N. Zhang , X.‐M. Tao , X. Ding , Adv. Healthcare Mater. 2017, 6, 1601371.

[17] Y. Jin , G. Chen , K. Lao , S. Li , Y. Lu , Y. Gan , Z. Li , J. Hu , J. Huang , J. Wen , H. Deng , M. Yang , Z. Chen , X. Hu , B. Liang , J. Luo , npj Flex. Electron. 2020, 4, 28.

[18] F. Gao , C. Liu , L. Zhang , T. Liu , Z. Wang , Z. Song , H. Cai , Z. Fang , J. Chen , J. Wang , M. Han , J. Wang , K. Lin , R. Wang , M. Li , Q. Mei , X. Ma , S. Liang , G. Gou , N. Xue , Microsystems Nanoeng. 2023, 9, 1. 10.1038/s41378-022-00443-6 PMC9805458 36597511

[19] M. Bariya , H. Y. Y. Nyein , A. Javey , Nat. Electron. 2018, 1, 160.

[20] Y. U Song , J. Min , Y. Yu , H. Wang , Y. Yang , H. Zhang , W. Gao , Sci. Adv. 2020, 6, aay9842. 10.1126/sciadv.aay9842 PMC7527225 32998888

[21] L. B. Baker , J. B. Model , K. A. Barnes , M. L. Anderson , S. P. Lee , K. A. Lee , S. D. Brown , A. J. Reimel , T. J. Roberts , R. P. Nuccio , J. L. Bonsignore , C. T. Ungaro , J. M. Carter , W. Li , M. S. Seib , J. T. Reeder , A. J. Aranyosi , J. A. Rogers , R. Ghaffari , Sci. Adv. 2020, 6, abe3929. 10.1126/sciadv.abe3929 PMC7732194 33310859

[22] A. Koh , D. Kang , Y. Xue , S. Lee , R. M. Pielak , J. Kim , T. Hwang , S. Min , A. Banks , P. Bastien , M. C. Manco , L. Wang , K. R. Ammann , K.‐I. Jang , P. Won , S. Han , R. Ghaffari , U. Paik , M. J. Slepian , G. Balooch , Y. Huang , J. A. Rogers , Sci. Transl. Med. 2016, 8, 366ra165. 10.1126/scitranslmed.aaf2593 PMC5429097 27881826

[23] T. He , J. Wang , D. Hu , Y. Yang , E. Chae , C. Lee , Nat. Commun. 2025, 16, 3244. 40185801 10.1038/s41467-025-58584-x PMC11971386

[24] Y. Yang , T. He , P. Ravindran , F. Wen , P. Krishnamurthy , L. Wang , Z. Zhang , P. P. Kumar , E. Chae , C. Lee , Sci. Adv. 2024, 10, adk7488. 10.1126/sciadv.adk7488 PMC10871535 38363835

[25] J. M. Nassar , S. M. Khan , D. R. Villalva , M. M. Nour , A. S. Almuslem , M. M. Hussain , npj Flex. Electron. 2018, 2, 24.

[26] W. Tang , T. Yan , F. Wang , J. Yang , J. Wu , J. Wang , T. Yue , Z. Li , Carbon N. Y. 2019, 147, 295.

[27] K. Lee , J. Park , M. I‐S. Lee , J. Kim , B. G. Hyun , D. J. Kang , K. Na , C. Y. Lee , F. Bien , J.‐U. Park , Nano Lett. 2014, 14, 2647. 24742260 10.1021/nl500513n

[28] B. Esser , J. M. Schnorr , T. M. Swager , Angew. Chem., Int. Ed. 2012, 51, 5752. 10.1002/anie.201201042 22517760

[29] R. Liu , L. He , M. Cao , Z. Sun , R. Zhu , Y. Li , Front. Chem. 2021, 9, 539678. 34631655 10.3389/fchem.2021.539678 PMC8492987

[30] Y. Lu , K. Xu , L. Zhang , M. Deguchi , H. Shishido , T. Arie , R. Pan , A. Hayashi , L. Shen , S. Akita , K. Takei , ACS Nano 2020, 14, 10966. 32806070 10.1021/acsnano.0c03757

[31] J. J. Kim , L. K. Allison , T. L. Andrew , Sci. Adv. 2019, 5, aaw0463. 10.1126/sciadv.aaw0463 PMC6420315 30899786

[32] C. B. Wetterich , R. Kumar , S. Sankaran , J. Belasque Junior , R. Ehsani , L. G. Marcassa , J. Spectrosc. 2013, 2013.

[33] K.‐Y. Huang , Comput. Electron. Agric. 2007, 57, 3.

[34] C. Hillnhütter , A.‐K. Mahlein , R. A. Sikora , E.‐C. Oerke , F. Crop. Res. 2011, 122, 70.

[35] J. J. Casanova , S. A. O'Shaughnessy , S. R. Evett , C. M. Rush , Sensors 2014, 14, 17753. 25251410 10.3390/s140917753 PMC4208247

[36] S. D. Bauer , F. Korč , W. Förstner , Precis. Agric. 2011, 12, 361.

[37] M. Schikora , B. Neupane , S. Madhogaria , W. Koch , D. Cremers , H. Hirt , K.‐H. Kogel , A. Schikora , BMC Bioinformat. 2012, 13, 1. 10.1186/1471-2105-13-171 PMC3519609 22812426

[38] L. Chaerle , D. Van Der Straeten , Biochim. Biophys. Acta (BBA)‐Gene Struct. Expr. 2001, 1519, 153. 10.1016/s0167-4781(01)00238-x 11418181

[39] A. Blum , Aust. J. Agric. Res. 2005, 56, 1159.

[40] N. McDowell , W. T. Pockman , C. D. Allen , D. D. Breshears , N. Cobb , T. Kolb , J. Plaut , J. Sperry , A. West , D. G. Williams , E. A. Yepez , New Phytol 2008, 178, 719. 18422905 10.1111/j.1469-8137.2008.02436.x

[41] M. M. Chaves , J. P. Maroco , J. S. Pereira , Funct. plant Biol. 2003, 30, 239. 32689007 10.1071/FP02076

[42] E. Fereres , M. A. Soriano , J. Exp. Bot. 2007, 58, 147. 17088360 10.1093/jxb/erl165

[43] A. Burquez , J. Exp. Bot. 1987, 38, 109.

[44] E. R. Hunt Jr , B. N. Rock , P. S. Nobel , Remote Sens. Environ. 1987, 22, 429.

[45] G. Sepulcre‐Canto , P. Zarco‐Tejada , J. Jimenez‐Munoz , J. Sobrino , M. Soriano , E. Fereres , V. Vega , M. Pastor , Remote Sens. Environ. 2007, 107, 455.

[46] P. J. Zarco‐Tejada , J. A. J. Berni , L. Suárez , G. Sepulcre‐Cantó , F. Morales , J. R. Miller , Remote Sens. Environ. 2009, 113, 1262.

[47] O. M. Grant , Ł. Tronina , H. G. Jones , M. M. Chaves , J. Exp. Bot. 2007, 58, 815. 17032729 10.1093/jxb/erl153

[48] X. Guo , L. Wang , Z. Jin , C. Lee , Nano‐Micro Lett. 2025, 17, 1. 10.1007/s40820-024-01587-y PMC11602912 39602030

[49] L. Wang , M. Xiao , X. Guo , Y. Yang , Z. Zhang , C. Lee , Biosensors 2024, 14, 629. 39727894 10.3390/bios14120629 PMC11674220

[50] D. Li , A. Yadav , H. Zhou , K. Roy , P. Thanasekaran , C. Lee , Glob. Challenges 2024, 8, 2300244. 10.1002/gch2.202300244 PMC10862192 38356684

[51] X. Guo , T. He , Z. Zhang , A. Luo , F. Wang , E. J. Ng , Y. Zhu , H. Liu , C. Lee , ACS Nano 2021, 15, 19054. 34308631 10.1021/acsnano.1c04464

[52] Z. Zhang , Q. Shi , T. He , X. Guo , B. Dong , J. Lee , C. Lee , Nano Energy 2021, 90, 106517.

[53] C. Wang , T. He , H. Zhou , Z. Zhang , C. Lee , Bioelectron. Med. 2023, 9, 17. 37528436 10.1186/s42234-023-00118-1 PMC10394931

[54] Q. Zhang , Q. Liang , D. K. Nandakumar , H. Qu , Q. Shi , F. I. Alzakia , D. J. J. Tay , L. Yang , X. Zhang , L. Suresh , C. Lee , A. T. S. Wee , S. C. Tan , Nat. Commun. 2021, 12, 616. 33504813 10.1038/s41467-021-20919-9 PMC7841174

[55] S. Lee , H. Wang , J. Wang , Q. Shi , S.‐C. Yen , N. V. Thakor , C. Lee , Nano Energy 2018, 50, 148.

[56] L. Wang , T. He , Z. Zhang , L. Zhao , C. Lee , G. Luo , Q. Mao , P. Yang , Q. Lin , X. Li , R. Maeda , Z. Jiang , Nano Energy 2021, 80, 105555.

[57] T. Wang , T. Jin , W. Lin , Y. Lin , H. Liu , T. Yue , Y. Tian , L. Li , Q. Zhang , C. Lee , ACS Nano 2024, 18, 9980. 38387068 10.1021/acsnano.3c11281

[58] D. Li , H. Zhou , Z. Ren , C. Lee , Small Sci 2025, 5, 2400250. 40657199 10.1002/smsc.202400250 PMC12245038

[59] J. Zhou , H. Zhang , Q. Qiao , H. Chen , Q. Huang , H. Wang , Q. Ren , N. Wang , Y. Ma , C. Lee , Nat. Commun. 2024, 15, 10260. 39592609 10.1038/s41467-024-54704-1 PMC11599558

[60] Y. Li , Z. Sun , M. Huang , L. Sun , H. Liu , C. Lee , Adv. Energy Sustain. Res. 2024, 5, 2400116.

[61] S. Duan , H. Zhang , L. Liu , Y. Lin , F. Zhao , P. Chen , S. Cao , K. Zhou , C. Gao , Z. Liu , Q. Shi , C. Lee , J. Wu , Mater. Today 2024, 80, 450.

[62] X. Liu , Z. Zhang , J. Zhou , W. Liu , G. Zhou , C. Lee , Small 2024, 20, 2400035. 10.1002/smll.202400035 38576121

[63] Z. Zhang , X. Guo , C. Lee , Nat. Commun. 2024, 15, 6465. 39085214 10.1038/s41467-024-50261-9 PMC11291476

[64] Z. Luo , D. Li , X. Le , T. He , S. Shao , Q. Lv , Z. Liu , C. Lee , T. Wu , Nanoscale 2024, 16, 10230. 38629471 10.1039/d3nr05684h

[65] M. Mitra , A. Kumar , S. Khandare , P. Gaddale , Y. Anandan , S. Pedibhotla , K. Roy , H. Chen , R. Pratap , S.‐R. (Raj) Kothapalli , in Photons Plus Ultrasound Imaging Sens (Eds: A. A. Oraevsky , L. V. Wang ), SPIE, San Francisco, CA 2024, p. 89. 10.1109/JSEN.2023.3344824 PMC10947080 38505656

[66] M. Mitra , A. Kumar , S. Khandare , P. Gaddale , Y. Anandan , S. Pedibhotla , K. Roy , H. Chen , R. Pratap , S.‐R. Kothapalli , IEEE Sens. J. 2023. 10.1109/JSEN.2023.3344824 PMC10947080 38505656

[67] K. Roy , S. Agrawal , A. Dangi , T. Liu , H. Chen , T. N. Jackson , IEEE Int. Ultrason. Symp. IUS 2020, 2020, 9251458.

[68] C. Wang , X. Chen , L. Wang , M. Makihata , H.‐C. Liu , T. Zhou , X. Zhao , Science 2022, 377, 517. 35901155 10.1126/science.abo2542

[69] L. Zhang , W. Du , J.‐H. Kim , C.‐C. Yu , C. Dagdeviren , Adv. Mater. 2023, 36, 2307664. 10.1002/adma.202307664 37792426

[70] H. Hu , H. Huang , M. Li , X. Gao , L. Yin , R. Qi , R. S. Wu , X. Chen , Y. Ma , K. Shi , C. Li , T. M. Maus , B. Huang , C. Lu , M. Lin , S. Zhou , Z. Lou , Y. Gu , Y. Chen , Y. Lei , X. Wang , R. Wang , W. Yue , X. Yang , Y. Bian , J. Mu , G. Park , S. Xiang , S. Cai , P. W. Corey , et al., Nature 2023, 613, 667. 36697864 10.1038/s41586-022-05498-z PMC9876798

[71] X. Gao , X. Chen , H. Hu , X. Wang , W. Yue , J. Mu , Z. Lou , R. Zhang , K. Shi , X. Chen , M. Lin , B. Qi , S. Zhou , C. Lu , Y. Gu , X. Yang , H. Ding , Y. Zhu , H. Huang , Y. Ma , M. Li , A. Mishra , J. Wang , S. Xu , Nat. Commun. 2022, 13, 7757. 36522334 10.1038/s41467-022-35455-3 PMC9755152

[72] L. Zhang , C. Marcus , D. Lin , D. Mejorado , S. J. Schoen , T. T. Pierce , V. Kumar , S. V. Fernandez , D. Hunt , Q. Li , I. I. Shuvo , D. Sadat , W. Du , H. Edenbaum , L. Jin , W. Liu , Y. C. Eldar , F. Li , A. P. Chandrakasan , A. E. Samir , C. Dagdeviren , Nat. Electron. 2024, 7, 77.

[73] H.‐C. Liu , Y. Zeng , C. Gong , X. Chen , P. Kijanka , J. Zhang , Y. Genyk , H. Tchelepi , C. Wang , Q. Zhou , X. Zhao , Sci. Adv. 2024, 10, adk8426. 10.1126/sciadv.adk8426 PMC10857377 38335289

[74] H. Hu , Y. Ma , X. Gao , D. Song , M. Li , H. Huang , X. Qian , R. Wu , K. Shi , H. Ding , M. Lin , X. Chen , W. Zhao , B. Qi , S. Zhou , R. Chen , Y. Gu , Y. Chen , Y. Lei , C. Wang , C. Wang , Y. Tong , H. Cui , A. Abdal , Y. Zhu , X. Tian , Z. Chen , C. Lu , X. Yang , J. Mu , et al., Nat. Biomed. Eng. 2023, 7, 1321. 37127710 10.1038/s41551-023-01038-w

[75] M. D. Fariñas , D. Jimenez‐Carretero , D. Sancho‐Knapik , J. J. Peguero‐Pina , E. Gil‐Pelegrín , T. Gómez Álvarez‐Arenas , Plant Methods 2019, 15, 1. 31709000 10.1186/s13007-019-0511-z PMC6836334

[76] D. Sancho‐Knapik , J. J. Peguero‐Pina , M. D. Fariñas , T. G. Álvarez‐Arenas , E. Gil‐Pelegrín , Tree Physiol 2013, 33, 695. 23933828 10.1093/treephys/tpt052

[77] D. Sancho‐Knapik , T. Gómez Álvarez‐Arenas , J. J. Peguero‐Pina , E. Gil‐Pelegrín , J. Exp. Bot. 2010, 61, 1385. 20176889 10.1093/jxb/erq001

[78] T. E. Gómez Álvarez‐Arenas , D. Sancho‐Knapik , J. J. Peguero‐Pina , E. Gil‐Pelegrín , Appl. Phys. Lett. 2009, 95, 193702.

[79] M. D. Fariñas , D. S. Knapik , J. J. P. Pina , E. G. Pelegrin , T. E. G. Álvarez‐Arenas , Ultrasound Med. Biol. 2014, 40, 2183. 25023117 10.1016/j.ultrasmedbio.2014.04.004

[80] V. Shastri , S. Talukder , K. Roy , P. Kumar , R. Pratap , ACS Omega 2022, 7, 12111. 35449943 10.1021/acsomega.2c00364 PMC9016874

[81] K. Roy , H. Gupta , V. Shastri , A. Dangi , A. Jeyaseelan , S. Dutta , R. Pratap , IEEE Sens. J. 2019, 20, 6802.

[82] K. Roy , H. Gupta , V. Shastri , A. Dangi , R. Pratap , IEEE Sens. 2018, 20, 1.

[83] V. Shastri , S. Talukder , K. Roy , P. Kumar , R. Pratap , Nanotechnology 2022, 33, 455301. 10.1088/1361-6528/ac83cc 35878592

[84] K. Roy , K. Kalyan , A. Ashok , V. Shastri , R. Pratap , in 21st International Conference on Solid‐State Sensors, Actuators and Microsystems (Transducers) , IEEE, Orlando, FL 2021, pp. 172–175.

[85] K. Roy , A. Mandal , A. Ashok , H. Gupta , V. Shastri , R. Pratap , IEEE Int. Ultrason. Symp. IUS 2020, 2020, 11.

[86] V. Shastri , S. Majumder , A. Ashok , K. Roy , R. Pratap , P. Kumar , Nanotechnology 2022, 34, 105301. 10.1088/1361-6528/aca76e 36537737

[87] K. Roy , K. Kalyan , A. Ashok , V. Shastri , R. Pratap , in 2021 IEEE International Ultrasonics Symposium (IUS) , Xi'an, China, September 2021.

[88] K. Roy , V. Shastri , A. Kalyan , Photons Plus Ultrasound: Imaging Sens. 2022, 11960, 282.

[89] K. Roy , S. Agrawal , A. Dangi , T. Liu , H. Chen , T. N. Jackson 2020 IEEE International Ultrasonics Symposium (IUS) , IEEE, Las Vegas, NV 2020, pp. 1–4.

[90] B. Nayak , H. Gupta , K. Roy , A. Ashok , V. Shastri , R. Pratap , in 2020 5th IEEE International Conference on Emerging Electronics (ICEE) , IEEE, New Delhi, India, 2020, pp. 1–4.

[91] A. Dangi , K. Roy , S. Agrawal , H. Chen , A. Ashok , C. Wible , M. Osman , R. Pratap , Sri‐R. Kothapalli , InPhotons Plus Ultrasound: Imaging Sens. 2020, 11240, 317.

[92] A. Paramanick , K. Roy , D. Samanta , K. S. Aiswarya , R. Pratap , M. S. Singh , Photons Plus Ultrasound: Imaging Sens. 2023, 12379, 386.

[93] H. Gupta , B. Nayak , K. Roy , A. Ashok , A. J. A. , R. Pratap , IEEE Int. Ultrason. Symp. IUS 2020, 2020, 1.

[94] K. Roy , K. Roy , A. Thomas , S. Paul , A. Ashok , V. Shastri , K. Kalyan , M. Suheshkumar Singh , R. Pratap , Microfluidics, BioMEMS, and Medical Microsystems XIX 2021, 11637, 89.

[95] K. Roy , A. Ashok , K. Kalyan , V. Shastri , A. Jeyaseelan , N. VeeraPandi , M. Nayak , R. Pratap , Photons Plus Ultrasound: Imaging Sens. 2021, 11642, 309.

[96] S. H. Paladugu , K. Roy , A. Ashok , B. Nayak , A. Rangarajan , R. Pratap , Appl. Phys. Rev. 2025, 12, 021407.

[97] A. Paramanick , K. Roy , D. Samanta , T. Das , R. Pratap , M. S. Singh , Photons Plus Ultrasound: Imaging Sens. 2025, 13319, 436.

[98] K. Roy , A. Kumar , V. Shastri , I. Munjal , K. Kalyan , A. Ashok , A. Jeyaseelan , J. Prakash , R. Pratap , IEEE Trans. Instrum. Meas. 2024, 73, 9510408.

[99] S. J. Z. Wong , K. Roy , C. Lee , Y. Zhu , IEEE Trans. Ultrason. Ferroelectr. Freq. Control 2024, 622. 38635378 10.1109/TUFFC.2024.3390807

[100] A. Paramanick , K. Roy , S. Paul , A. Kumar , A. Ashok , R. Pratap , M. S. Singh , IEEE Sensors Lett. 2024, 8, 1.

[101] E. Moisello , L. Novaresi , E. Sarkar , P. Malcovati , T. L. Costa , E. Bonizzoni , IEEE Access 2024, 12, 18640.

[102] K. Roy , K. Kalyan , A. Ashok , V. Shastri , A. Antony Jeyaseelan , A. Mandal , J. Microelectromech. Syst. 2021, 30, 642.

[103] R. Pratap , A. Dangi , K. Roy , H. Gupta , ECS Trans. 2018, 86, 13.

[104] S. Akhbari , F. Sammoura , C. Yang , M. Mahmoud , N. Aqab , L. Lin , 2015 28th IEEE International Conference on Micro Electro Mechanical Systems (MEMS) , Estoril, Portugal, Jan, 2015, 928.

[105] S. Akhbari , A. Voie , Z. Li , B. Eovino , L. Lin , 2016 IEEE 29th International Conference on Micro Electro Mechanical Systems (MEMS) , Shanghai, China, Jan, 2016, 1102.

[106] T. Wang , R. Sawada , C. Lee , IEEE Electron Device Lett. 2015, 36, 957.

[107] T. G. Álvarez‐Arenas , D. Sancho‐Knapik , J. J. Peguero‐Pina , E. Gil‐Pelegrín , Proc. ‐ IEEE Ultrason. Symp, Rome, Italy, Sep, 2009, 771.

[108] A. I. K. S. Rupp , P. Gruber , Biomimetics 2019, 4, 75. 31783650 10.3390/biomimetics4040075 PMC6963917

[109] K. Roy , J. E.‐Y. Lee , C. Lee , Microsystems and Nanoeng. 2023, 9, 95. 10.1038/s41378-023-00555-7 PMC10359338 37484500

[110] T. G. Álvarez‐Arenas , D. Sancho‐Knapik , J. J. Peguero‐Pina , E. Gil‐Pelegrín , Proc. ‐ IEEE Ultrason. Symp, Rome, Italy, Sep, 2009, 771.

[111] J. J. Kim , L. K. Allison , T. L. Andrew , Sci. Adv. 2019, 5, aaw0463. 10.1126/sciadv.aaw0463 PMC6420315 30899786

[112] U. Zimmermann , D. Zimmermann , R. Reuss , M. Westhoff , P. Geßner , W. Bauer , E. Bamberg , F‐W. Bentrup , J. Exp. Bot. 2008, 59, 3157. 18689442 10.1093/jxb/ern171 PMC2504341

[113] E. R. Hunt , B. N. Rock , P. S. Nobel , Remote Sens. Environ. 1987, 22, 429.

[114] M. M. Farinas , D. Jimenez‐Carretero , D. Sancho‐Knapik , J. J. Peguero‐Pina , E. Gil‐Pelegrín , T. G. Álvarez‐Arenas , Plant Methods 2019, 15, 128. 31709000 10.1186/s13007-019-0511-z PMC6836334

[115] M. A. Atherton , G. R. Tucker , D. R. Myers , Sens. Actuators A Phys. 2012, 187, 67.

[116] S. Yin , L. Dong , Adv. Mater. Technol. 2024, 9, 2302073.

[117] R. Li , Y. Lu , J. M. R. Peters , B. Choat , A. J. Lee , Sci. Rep. 2020, 10, 21028. 33273649 10.1038/s41598-020-78154-z PMC7713062

[118] L. Zheng , Z. Wang , H. Sun , M. Zhang , M. Li , Comput. Electron. Agric. 2015, 112, 102.

[119] H. Afzal , M. B. Bashir , H. Iqbal , D. Anderson , Biosyst. Eng. 2017, 156, 148.

[120] T. Liu , M. Zhang , Z. Li , H. Dou , W. Zhang , J. Yang , P. Wu , D. Li , X. Mu , Nat. Commun. 2025, 16, 2363. 40064879 10.1038/s41467-025-57629-5 PMC11894117

[121] M. Teng , W. Yue , Y. Peng , P.‐C. Tsao , H. Deng , F. Xia , 2024 IEEE 37th International Conference on Micro Electro Mechanical Systems (MEMS) , Austin, TX Jan, 2024, 971.

[122] T. Leng , L. Li , C. Lee , AI Sensors 2025, 1, 1.

