# Supplementary material for: A Generative AI‐Assisted Piezo‐MEMS Ultrasound Device for Plant Dehydration Monitoring
Source: Adv Sci (Weinh). 2025 Jun 19;12(32):e04954. doi: 10.1002/advs.202504954 (PMC12407371; doi:10.1002/advs.202504954)
Supplement: Supplementary file 1 — Supporting Information [file ADVS-12-e04954-s001.pdf]

ADVANCED SCIENCE
Open Access

Supporting Information

for *Adv. Sci.*, DOI 10.1002/advs.202504954

A Generative AI-Assisted Piezo-MEMS Ultrasound Device for Plant Dehydration Monitoring

*Kaustav Roy, Darren Sim, Luwei Wang, Zixuan Zhang, Xinge Guo, Yao Zhu, Sanjay Swarup and Chengkuo Lee**

Supplementary Information

A Generative AI-Assisted Piezo-MEMS Ultrasound Device for Plant Health Monitoring

*Kaustav Roy[1,2], Darren Sim[3], Luwei Wang[1,2], Zixuan Zhang[1,2], Xinge Guo[1,2], Yao Zhu[4], Sanjay Swarup[3], and Chengkuo Lee[1,2]**

[1] Department of Electrical and Computer Engineering, National University of Singapore, Singapore 117576, Singapore

[2] Center for Intelligent Sensors and MEMS (CISM), National University of Singapore, Singapore 117583, Singapore.

[3] Department of Biological Sciences, National University of Singapore, Singapore 117543,

[4] Institute of Microelectronics (IME), Agency for Science, Technology and Research (A*STAR), Singapore 117685, Singapore

*E-mail: (C. L.) elelc@nus.edu.sg

Table of Contents

I. Our Work, Novelty, and Impact 3

II. Piezo-MEMS Ultrasound and the PMUT-Leaf-PMUT (PLP)

A. PMUT Chip Nomenclature: Die, Element and Cell 9
B. Laser Doppler Vibrometry of Different PMUT Cells & Acoustic Attenuation 11
C. Piezoelectric over Silicon-on-Nothing (PSON) PMUT Fabrication Process Flow 14
D. Mechanism of PMUT's Vibration 16
E. PMUT Electrical Impedance 19
F. PMUT Element Response Characterization 21
G. Process Flow for Making the PMUT-Leaf-PMUT Clip 23
H. Spacer Optimization 25
I. PLP's Time Domain Acoustic Response 27
J. Finite Element Simulation (Part I): PMUT's Vibroacoustic Response 29
K. Finite Element Simulation (Part II): PMUT-Air-PMUT 31
L. Finite Element Simulation (Part III): PMUT-Leaf-PMUT 33
M. Setup Used to Detect RWC using PLP 36

III. PLP Device on the Leaf

A. Leaf Water Content Metrics 37
B. RWC Determination Techniques: Our Work's Novelty 39
C. PMUT-Leaf-PMUT Working Principle 43
D. Detached Leaf Experiment Using PLP 48
E. Whole Plant Experiment Using PLP 50
F. Growth Chamber Used for Experiment 52

IV. Machine Learning with PLP on Plants

A. Cultivars of sweet potato plant used in the project 53
B. Reason for using generative model such as CVAE as the deep learning algorithm 55
C. Steps to Find leaf features such as the area, perimeter, and skewness 59
D. Leaf probing zones for obtaining training data 60
E. Looks of a sample data set for training the ML models 61
F. Model Training Curves 62
G. Structure of the CVAE ML model 64
H. Effect of increase in training data on ML prediction 65
I. Experimental setup for whole plant experiments 66

Supplementary Note I: Our Work, Novelty, and Impact

In this work we have constructed a precision agricultural [1], [2], plant wearable device [3], [4] which is capable of: (a) measuring leaf relative water content (RWC) in a single shot within a second of time, (b) being re-attached to any leaf of any plant for indefinite number of times, and (c) remaining attached to any leaf of any plant for sufficiently long duration and continuously measure its RWC, thereby tracking the plant's health. The device does this by non-invasively probing a leaf with ultrasound waves and works on the principle of sound attenuation [5]. As a result, the device looks like a clip, having two arms: one bearing the sound transmitter and the other the sound receiver (Fig. S1A). Now since the sound gets transmitted from the transmitter into air then to the leaf and back to the air then to the receiver, it required us to employ air-coupled ultrasound transducers [6]. Thus, in the heart of the transmitter/receiver is a CMOS compatible piezoelectric micromachined ultrasound transducer (PMUT) [7] which not only can work as an air-coupled transmitter/receiver but also is small, lightweight and low-powered. After several trials of optimization, we found the correct package in order to make the clip device (Fig. S1B). Once the device was made, it went through elaborate cycles of vibroacoustic characterization for its repeatability (Fig. S1C) and was successfully tested in different wet materials as a water content detector (Fig. S1D). Such as test revealed the initial capability of our device and the idea of using in-air ultrasound to work as RWC detector. Once the clip's functioning was established as a water content detector, we went ahead to test the clip's functioning in plant tissues, specifically in separated leaves from various plants (Fig. S1E). These leaves had different phenotypes and morphologies. The test revealed several important aspects pertaining to the water dynamics in separated leaves of different phenotypes. Now, till this point, all the dynamics and physical phenomena that were being observed had the device voltage as the indicating parameter without being strictly associated to the RWC of the leaf. Now, we realized that an attempt to connect the voltage readout to the RWC value needs some sort of machine intelligence since there is a natural diverseness of each leaf (leaves being highly nonlinear from the material constitution and functioning thereof) that exists on a plant and resulting in a broad diverseness in the voltage readout. To capture such a diverseness, there is a need to have leaf specific curves in the form of nonlinear polynomial functions that would connect the device voltage to the corresponding level of RWC. Such a curve can only be generated by using generative AI when trained for a large dataset of leaves. Thus, we used a conditional variational autoencoder [8] (structure shown in Fig. S1F) and trained it with a library of 200 curves, resulting in the concept of feature

specific RWC prediction (Fig. S1G). We then tested the functioning of our model with unknown datasets and achieved reasonably minimal error in RWC prediction. As a result, the AI-assisted device now becomes a full-fledged RWC detector. After creating such a platform, we exhibited the device's proficiency in RWC detection for two scenarios (Fig. 1SH): (a) One-shot RWC measurement from any unknown leaf with measured geometrical features. This scenario can be compared to the measurement of blood oxygen saturation in humans via one-shot pulsed oximetry, thereby reporting the value of oxygen content in the blood for a single time. Such kind of oximeters can be used for several different patients; (b) Long-term continuous monitoring of RWC by attaching the device onto a leaf. This scenario can be compared to the measurement of blood oxygen saturation continuously over time by attaching the sensor onto a finger.

The work's novelty can be summarized as follows (see Fig. S1I):

Novelty in the application of PMUTs on plants: To the best of our knowledge, this is for the first time we demonstrate the applicability of PMUTs in the domain of precision agriculture to measure leaf water content. Air coupled PMUTs, have previously been used for range finding [9]–[12], data over sound [13], haptics [14], [15] etc., but their application in the domain of agriculture is still un-heard of. The use of PMUTs comes with several advantages such as: CMOS compatibility and miniaturize-ability, low power consumption [16] and tailorable form factor, thereby enabling the making of advanced ultrasound-based plant wearables. CMOS compatibility enables the integration of CMOS circuits such as amplifiers, analogue to digital convertors, and wireless communication along with the PMUTs on the same chip, thereby decreasing the overall bulkiness of the device, and making it more plant wearable. Since PMUTs operate at lower voltages (< 1V) as compared to the bulk ultrasound transducers, and consume significantly lower power, it is possible to power the overall system using green energy like solar, making it further sustainable and energy efficient in outdoor on-plant deployment. Furthermore, with the advantage of advanced manufacturing following lithographic patterning, it is possible to make the PMUTs in any form factor and in any die geometries enabling placement of the devices in strategic locations on leaves for RWC determination. Thus, the successful use of PMUTs in our work as air-coupled transducers to detect deep-tissue parameters in plants opens up new opportunities for their use in similar precision agriculture applications.

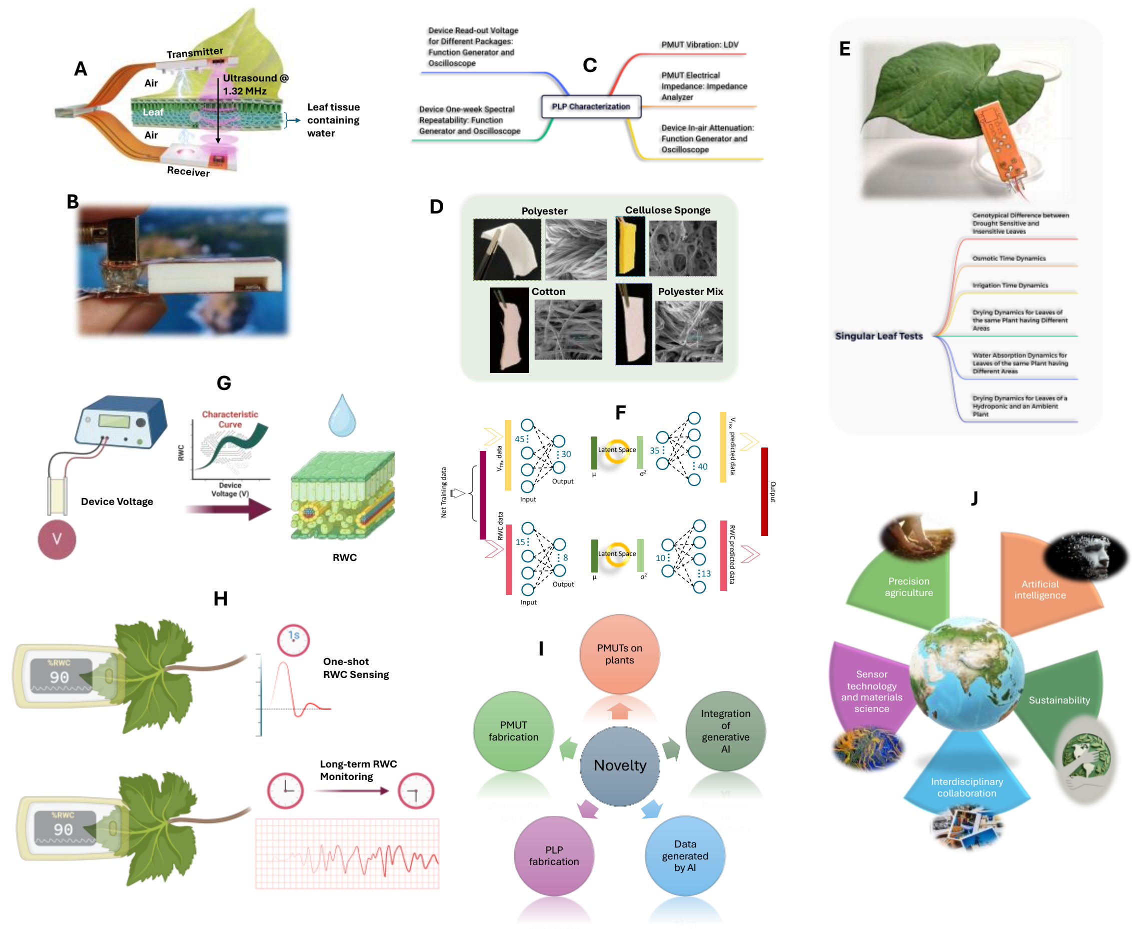

Supplementary Figure S1: Our work, novelty, and impact. A. Our proposed RWC measurement device PLP. B. PLP device in reality with optimized package bounding the PMUTs. C. Various PLP vibroacoustic characterizations. D. Wet materials for which the PLP could detect the water content. E. PLP on a detached plant leaf. F. The generative AI model which used CVAE as the algorithm for generating calibration curves. G. The concept of feature specific RWC prediction using PLP and AI. H. PLP's capability as a RWC sensor for two use cases. I. Novelty of our proposed work. J. Probable impact of our proposed work.

Novelty in the making of the PMUTs: To the best of our knowledge, this is for the first time we demonstrate the making of PMUTs following a novel process flow employing the silicon migration technique [17], [18] to create flexible vibroacoustic membranes. We name this process piezoelectric over silicon-on-nothing (pSON) [19], [20] and have recently showcased the technology to the world in conference pitches. The process enables the making of PMUTs which can be both air and water coupled. In addition, this technology offers the technical advantage of one-shot manufacturing of PMUTs having multiple different sizes on the same chip, thereby enabling the multi-frequency feature in a PMUT chip. Since the process can be realized by using surface micromachining approach (although we have used bulk micromachining for etching initial holes of 25 μm depth) the cost of manufacturing is

significantly lowered, thereby making it sustainable for mass manufacturing. In addition, the process is simple (without any top-bottom lithographic alignment which considerably increases the process complexity), and it leads to uniform characteristics all through the wafer, thereby making the technology more promising to adapt.

Novelty in the make of PMUT-Leaf-PMUT (PLP) device: Our final PLP device can: (a) get attached and detached to and from any leaf for indefinite number of cycles, (b) take one-shot RWC measurement, (c) be attached on any leaf for long time and monitor RWC. These capabilities in a single sensor package is unique to the best of our knowledge and has not been previously demonstrated. Previous reported re-attachable device also worked on ultrasound but is far bulkier than PLP, thereby limiting its plant wearability for long time RWC monitoring mounted on a leaf [21]. In addition, such a device is not capable of taking one-shot RWC measurements to our understanding. The second category of devices which are reported are fully plant wearable, generally made up of polymers with coated functional layers [22]. However, such devices come with the limitation of re-attachability, thereby limiting their usages to only one time. In other words, once those devices are attached to leaf surfaces, it is not possible to remove them and again affix on the same/other leaf for re-reading of RWC.

Novelty in the integration of generative AI with device readout: This is for the first time, we have reported on using generative AI model such as the CVAE to aid in leaf RWC determination, thereby re-demonstrating the world the capability of generative AI in the domain of precision agriculture. In general, generative models have been used in precision agriculture in the context of data augmentation and image analysis, but the mention of such a capability of AI to interpret sensor data and work as a calibration curve generator has been entirely missing. This demonstration is believed to help broadening the horizon of the applicability of AI in sensor data interpretation [23]–[25]. It is believed without the aid of machine learning it is way too complicated to accurately associate sensor voltage to leaf-RWC value owing to the natural diverseness of the leaves present in a plant in terms of tissue non-uniformity, material distribution, and material mechanoacoustic characteristics.

Novelty in terms of data generated by using the AI-assisted PLP: After successful integration of the PLP with the generative AI model, we could easily in a single shot assess any leaf's RWC, and we have demonstrated this for six different leaves from three different plant cultivars, subjected to varying environments of irrigation. In addition to this, we could also attach the PLP on leaves of 3 different whole plants at a time and continuously extract and plot

the RWC for the specific leaf the device was attached to, for almost 3 days of time. In such scenarios, we could controllably vary environmental parameters such as the temperature, relative humidity, and available light intensity along with intermittent irrigation and demonstrate the dynamicity involved in a plant's response to such changes. Although many different groups have tried to monitor plant's health via different routes, such an involved study has not been reported yet, and thus these data that we have obtained is believed to be novel.

We predict that our work may impact the domains of precision agriculture, sensor technology and material science, artificial intelligence, sustainability and interdisciplinary collaboration in the following ways (Fig. S1J):

1. Precision Agriculture

- Innovative Monitoring: The ability of the device to non-invasively measure leaf relative water content (RWC) in real-time enables precision monitoring of plant health, leading to more efficient water and resource management in agriculture.
- Data-Driven Farming: By integrating this device across crops, farmers can access a wealth of data to make informed decisions on irrigation and crop management, potentially increasing yield while reducing resource waste.

2. Sensor Technology and Materials Science

- PMUT Advancements: The novel application and manufacturing process of air-coupled PMUTs described expands the use of sensor technology in new domains, demonstrating its versatility and adaptability.
- Material Innovation: The creation of flexible vibroacoustic membranes using a silicon migration technique showcases advancements in materials science, particularly in the development of sensors and wearable technologies for plants.

3. Artificial Intelligence (AI)

- AI in Precision Agriculture: Employing a conditional variational autoencoder (CVAE) for leaf RWC prediction illustrates the powerful role AI can play in interpreting complex biological data. This approach could pave the way for more sophisticated AI applications in agriculture, from pest detection to nutrient management.

- Generative Models for Calibration: The use of generative AI to create calibration curves for sensor data interpretation could revolutionize sensor-based technologies in various fields, making them more adaptable and accurate.

4. Sustainability

- Energy Efficiency: The low power consumption of PMUTs and the potential for solar power integration highlight the device's sustainability aspect. Such innovations are crucial for developing eco-friendly technologies that reduce the environmental footprint of agricultural practices.
- Resource Optimization: Precise water content measurement can lead to more sustainable water use, addressing one of the most pressing challenges in agriculture—water scarcity.

5. Interdisciplinary Collaboration

The development of this device is a testament to the benefits of interdisciplinary research, combining insights from engineering, materials science, plant sciences, and computer science. This collaborative approach can inspire future projects that tackle complex problems by integrating diverse fields of study.

Supplementary Note II: Piezo-MEMS Ultrasound and the PMUT-Leaf-PMUT (PLP)

This section describes the various aspects of the piezo-MEMS [26]–[29] ultrasound device also known as the piezoelectric micromachined ultrasound transducer (PMUT) [7], [30], [31] used in the manuscript. In addition, it also defines the relative water content [32], [33] measuring device proposed in the manuscript called the PMUT-Leaf-PMUT (PLP) and discusses various engineering aspects of it.

A. PMUT Chip Nomenclature: Die, Element and Cell

Next, it is important to understand the PMUT chip nomenclature as used in the manuscript. The PMUTs were fabricated from the cleanroom in the form of mesoscopic chips. These chips are called dies. Now, each PMUT die consists of islands of interconnected devices. We call these islands elements. The PMUT die we use have 4 different elements. Elements can be

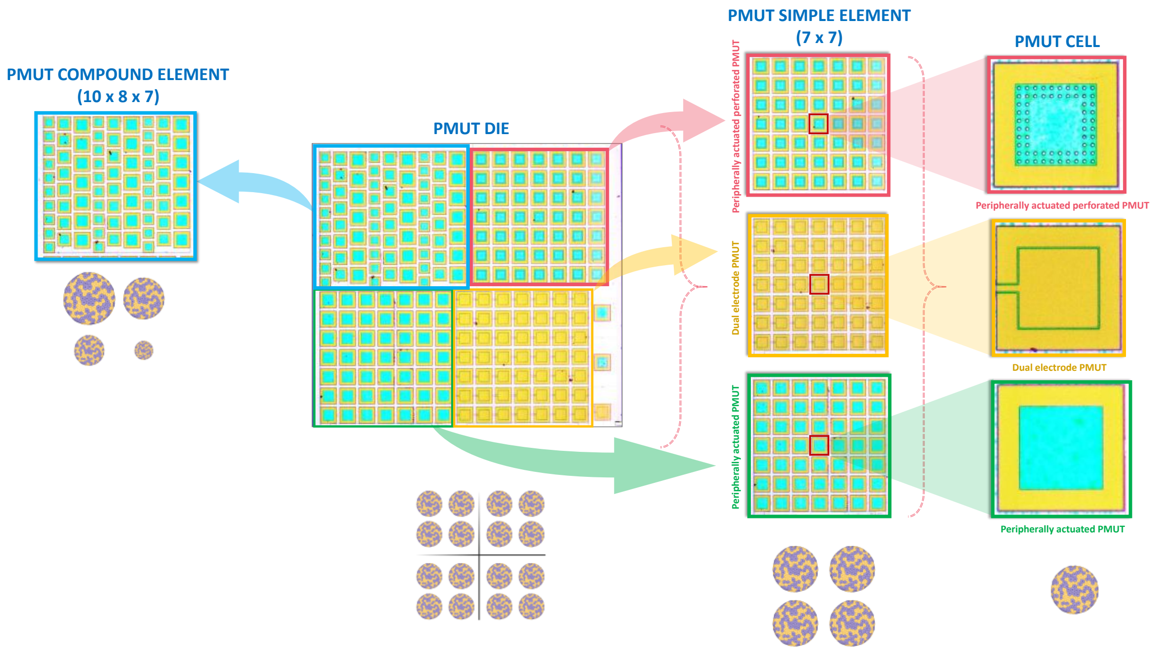

Supplementary Figure S2: PMUT chip nomenclature: definition of die, element and cell.

further divided into a singular unit called cells. Each cell is a PMUT device capable of transmitting and receiving ultrasound. There are a total of 3 different kinds of cells which constitute the elements. They are: peripherally actuated perforated PMUT [34], dual electrode PMUT [35]–[38] and the peripherally actuated PMUT respectively. Now the elements can be further classified into two subgroups: simple element and compound element. A simple element

comprises of similar type of PMUT cells, whereas the compound element consists of different types of cells connected together to form a sub-element. For example, the die depicted in the Fig. S2 contains 3 simple elements containing 7 x 7 cells all connected together. In other words, these cells vibrate together when excited using AC voltage to produce sound to produce acoustics, and vice versa. The PMUT die also contain 1 compound element having 3 sub-elements containing cells in 10 x 8 x 7 arrangement. So, as can be seen from the Fig. S2, each sub element will have 10 PMUT cells of one kind (100 µm in dimension), 8 PMUT cells of the other kind (150 µm in dimension) and 7 remaining PMUT cells (200 µm in dimension). In this case, the entire sub element can be accessed electrically, which means it is possible to excite each sub element individually. For our project, we have used the dual electrode PMUT element as described in Fig. S2 for both transmission and reception.

B. Laser Doppler Vibrometry of Different PMUT Cells & Acoustic Attenuation

Optical microscopy of the device is the rudimentary characterization which enables to observe any superficial structural/functional flaws in the device. Microscopy depicts the presence of 4 different kinds of device islands with 3 different kinds of PMUT cells such as: (1) the peripherally actuated PMUT having actuation electrode in the device periphery, (2) dual

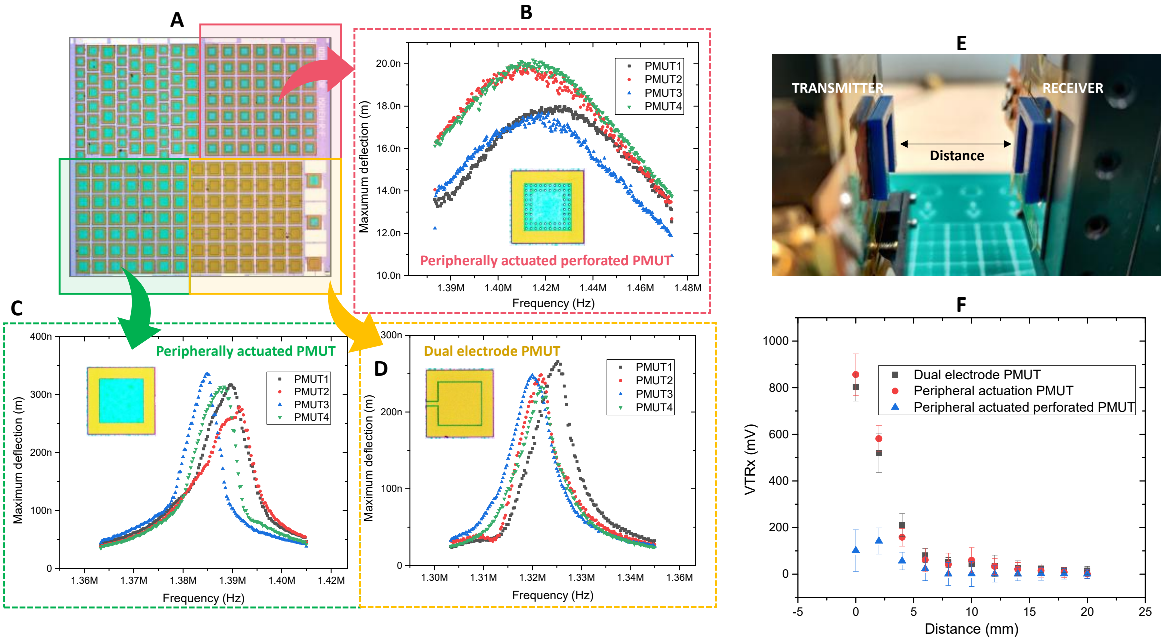

Supplementary Figure S3: Vibroacoustic characterization of different PMUT elements in PMUT die. A. Optical microscope of PMUT die. B. Displacement response of a peripheral actuated perforated PMUT showing broadband in air response, with lower maximum deflection. C. Displacement response of a dual electrode PMUT when driven by the central electrode. D. Displacement response of a peripheral actuated PMUT. E. Experimental setup showing the transmitter PMUT die and the receiver PMUT die kept facing each other mounted on two 3-axis stages F. In-air ultrasound attenuation from PMUTs of three different kinds showing the appropriateness of using the dual electrode variant for the experiments.

electrode PMUT having two separate electrodes: one central electrode for actuation and another peripheral electrode for sensing/actuation and the (3) peripherally actuated PMUT with peripheral perforations.

In order to understand the performance of the various PMUT cells, laser doppler vibrometer [39]–[41] was used. A vibrometer typically functions as a dual-beam laser interferometer, which assesses the frequency (or phase) variance between an internal benchmark beam and a beam for testing. Helium-neon lasers are the most prevalent laser source in an LDV, but laser diodes, fiber lasers, and Nd:YAG lasers are also utilized. The beam meant for testing is aimed

at the object of interest, and the light that bounces off the object is gathered and combined with the benchmark beam on a light-sensitive detector, often a photodiode. To operate in a heterodyne manner, most off-the-shelf vibrometers introduce a predetermined frequency alteration (usually around 30–40 MHz) to one of the beams, typically achieved through a Bragg cell or an acousto-optic modulator. Four different cells each from the three different device islands were tested using the LDV in the peak-hold mode of the Fast Fourier Transform (FFT) analysis. The peak hold mode of testing determines the actual deflection that a PMUT's membrane will exhibit. The devices can be characterized by knowing the deflection sensitivity which is the maximum peak-hold deflection per unit voltage. The peripherally actuated PMUTs showed the maximum deflection having values ~ 300 nm/V at a frequency of ~ 1.38 MHz. The dual electrode PMUT exhibited deflection sensitivity of ~ 250 nm/V at a frequency of 1.32 MHz. The peripherally actuated perforated PMUTs demonstrated a maximum deflection sensitivity of ~18 nm/V (which is an order of magnitude lesser than the other two PMUT cell varieties), at a frequency of 1.42 MHz. The peripheral perforations mostly serve the purpose of vibroacoustic dampers, by providing a free passage of air in and out the PMUT acoustic cavity and the ambient environment. This sliding of air through the perforations/holes provides a vibration damping route through air friction, thereby decreasing the Quality factor (Q factor) of the device. Q factor can be defined as:

$$Q\ factor = 2\pi \frac{Energy\ stored}{Energy\ lost\ per\ cycle}$$

Thus, from the equation if the energy lost per cycle is more which is the case of perforated PMUTs, then the Q factor decreases.

To finally select the desired device in order to construct the leaf compatible clip device, it is important to consider the acoustic response from a transmitter-receiver arrangement when the distance between the transmitter and the receiver is varied. The clip design required devices (the transmit-receive pair) which will perform the best at a distance greater than 3 mm. An experiment was conducted to vary the distance from 0 mm to 20 mm in steps of 2mm, and three sets of attenuation plots were obtained from three different kinds of PMUTs. The peripherally actuated PMUT as expected from the deflection response, produced a lower acoustic pressure (nearly 4 times lower than the other two PMUTs), and thus was rejected to be used in the clip. The next competitors were the dual electrode PMUT and the peripherally actuated PMUT. The peripherally actuated PMUT started off with a higher acoustic pressure

as indicated through the V_{TRx} transceive response but loses its way to the dual electrode PMUT after 2 mm of the gap distance. This can be attributed to the fact that the peripherally actuated PMUTs had a higher resonant frequency as compared to the dual electrode PMUT, which makes the sound fall down steeper in intensity as compared to its low frequency counterpart. Theoretically, the slope of the fall is determined by the acoustic attenuation coefficient (α), given by:

$$\alpha = \frac{2\omega^2\eta}{3\rho_0 c^3}$$

Where ω is the angular frequency in radians, η is the shear viscosity, ρ is the medium density and c is the speed of sound in the concerned media.

C. Piezoelectric over Silicon-on-Nothing (PSON) PMUT Fabrication Process Flow

The process flow for fabricating PSON PMUT [9], [19], [20] is described in the following steps and pictorially represented in the supplementary figure S2.

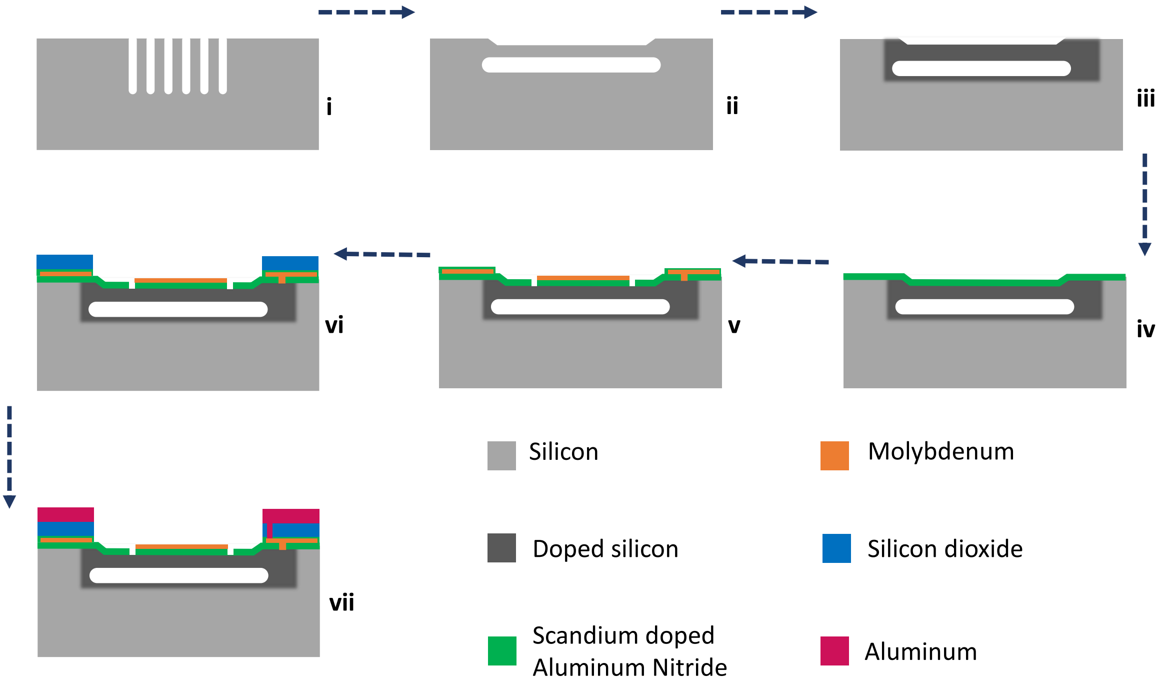

Supplementary Figure S4: Fabrication process flow for the PSON PMUT.

The steps are as followed:

i: Lithography patterning and deep etching using the Deep Reactive Ion Etching (DRIE) bulk silicon to form micro cavities.

ii: Annealing of the resulting wafer in a hydrogen ambient and high temperature, low pressure to form the silicon-on nothing (SON) structure with a 2 µm membrane over an approximately 1 µm deep cavity.

iii: Local diffusion (n++) using a silicon oxide mask to form the bottom electrode.

iv: Deposition of 300 nm of Sc0.15Al0.85N (ScAlN) by reactive sputtering.

v: Lithography patterning and reactive ion etching (RIE) of the ScAlN layer to define the future provisions for the actuation, sensing, and ground electrodes. This was followed by the deposition of 200 nm of Molybdenum as the functional electrode layer. This was followed by another round of deposition of ScAlN to insulate naked traces and subsequent oxidation of the Mo electrodes.

vi: Patterned deposition of 2 µm silicon oxide using plasma enhanced chemical vapour deposition (PECVD) for electrical isolation.

vii. Patterning of the silicon oxide thereby following a reactive ion etch to define vias to connect the Molybdenum layer. This was followed by patterned deposition of the Aluminium layer to define the bond pads.

D. Mechanism of PMUT's Vibration

PMUTs vibrate out-of-plane to transmit/receive sound [42], and they are generally fabricated in a circular geometry. At their fundamental frequency of vibration, the vibrating shape is best captured by a modified parabolic shape.

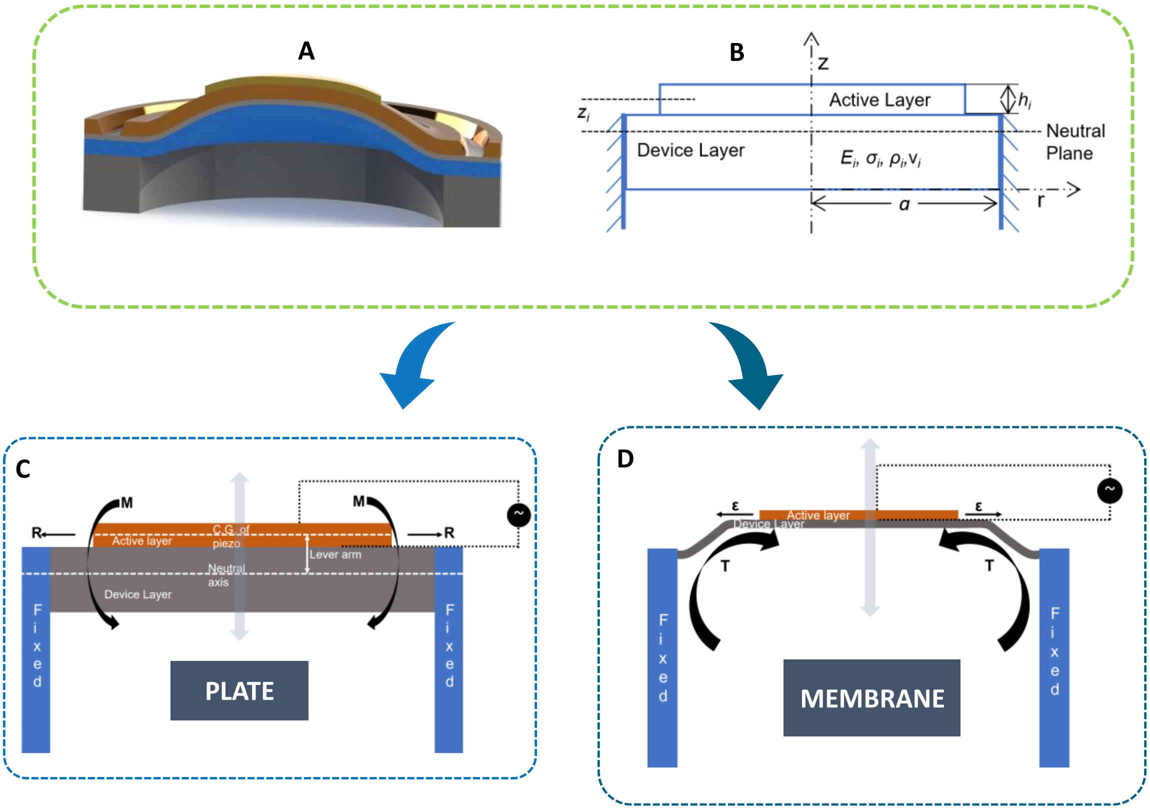

Supplementary Figure S5: How does a PMUT vibrate? A. A PMUT in its deformed shape when vibrating. B. Structurally important layers in a PMUT. C. Schematic representing the vibration mechanics of a plate PMUT. D. Schematic representing the vibration mechanics of a membrane PMUT.

Structurally, a PMUT comprises two important layers: the device layer and the piezoelectric-active layer. The device layer contains the neutral plane, with the centroid of each layer at a distance of z_i from the neutral plane. Each layer has a thickness, elasticity, prestress, density, and Poisson's ratio of h_i, E_i, σ_i, ρ_i and v_i, respectively. The radius of the device layer is a. The working principles of plate and membrane PMUTs are different.

For a plate PMUT, applying direct current (DC) voltage across the piezoelectric layer tends to strain it due to the d31 piezoelectric effect. This strain is then restricted by the underlying device layer, leading to in-plane normal stress resultant R working from the piezoelectric layer's centroid. Since plate PMUTs are thick, the neutral axis rests in the device layer. The difference (lever arm) between the piezoelectric layer's centroid and the neutral axis, along with R, results

in a bending moment M that bends the structure in an out-of-plane manner. Thus, applying an AC voltage makes the structure vibrate. Alternatively, for a membrane PMUT, the membrane is already stressed due to the presence of a net pretension in the structure, which, when coupled with structural inhomogeneity, causes the PMUT to bend without an electric field. A DC voltage induces a certain level of strain (ε) in the structure, which may occur due to the comparable thickness of the device and piezoelectric layer or due to a larger size-to-stack thickness ratio changing the level of tension (τ) in the structure, thereby changing the magnitude of bending. Applying an AC voltage makes the structure vibrate.

In this context it is important to be aware of the basic mathematical equations which govern the PMUT vibration mechanics:

Equation of motion of a flex tensional PMUT is given by:

$$D_e \nabla^4 w - T_e \nabla^2 w + \rho_h \frac{\partial^2 w}{\partial t^2} = f(r, \theta, t)$$

Mode shape of a flex tensional PMUT is given by:

$$\psi_{mn}(r, \theta) = \left[J_n \left(\frac{\alpha_{mn} r}{a} \right) - \frac{J_n(\alpha_{mn})}{I_n(\beta_{mn})} I_n \left(\frac{\beta_{mn} r}{a} \right) \right] \cos n\theta$$

Natural frequency of a flex tensional PMUT is given by:

$$\sqrt{\frac{\alpha_{mn}^2 D_e}{\rho_h a^4} \left(\alpha_{mn}^2 + \kappa^2 \right)}$$

Deflection of a flex tensional PMUT is given by:

$$w(r, t) = \left(\frac{2 M_p V_{\text{in}}}{\rho_h a^2 \omega_{mn}^2} \right) \frac{\Lambda'}{\Lambda_2} \frac{\psi_{mn}(r) e^{j(\omega t - \phi)}}{\sqrt{(1 - \gamma^2)^2 + (2\zeta\gamma)^2}}$$

Non-dimensional parameters used to define the deflection equation is given by:

$$\Lambda' = r_p \left[\left. \frac{\partial \psi_{mn}}{\partial r} \right|_{r = r_p} \right]$$

$$\Lambda_2 = \frac{\int_0^a \psi_{mn}^2 r dr}{\int_0^a r dr}$$

Meaning of various symbols:

w – displacement response of the PMUT in the z-axis, ρ_h – mass per unit area, f – forcing function, ψ_{mn} – mode shape for (m,n) nodal parameters, α_{mn}, β_{mn} – mode dependent constants, m – number of nodal circles, n – number of nodal diameters, J_n – Bessel function of the first kind, I_n – modified Bessel function of the first kind, ω_{mn} – natural frequency of vibration, M_p –

moment load per unit length per unit voltage, V_{in} – input AC voltage, γ – ratio of forced frequency to natural frequency, ζ – damping ratio, r_p – top electrode radius.

E. PMUT Electrical Impedance

The electrical impedance of an acoustic resonator refers to the measure of opposition that the resonator presents to the flow of electric current when it is excited by an acoustic wave. This impedance is crucial because it characterizes how the resonator converts acoustic energy into electrical signals and vice versa, which is vital for designing and optimizing acoustic devices like microphones, speakers, and sensors. The impedance (*Z*) is a complex quantity that combines resistance (*R*), which dissipates energy, and reactance (*X*), which stores and releases

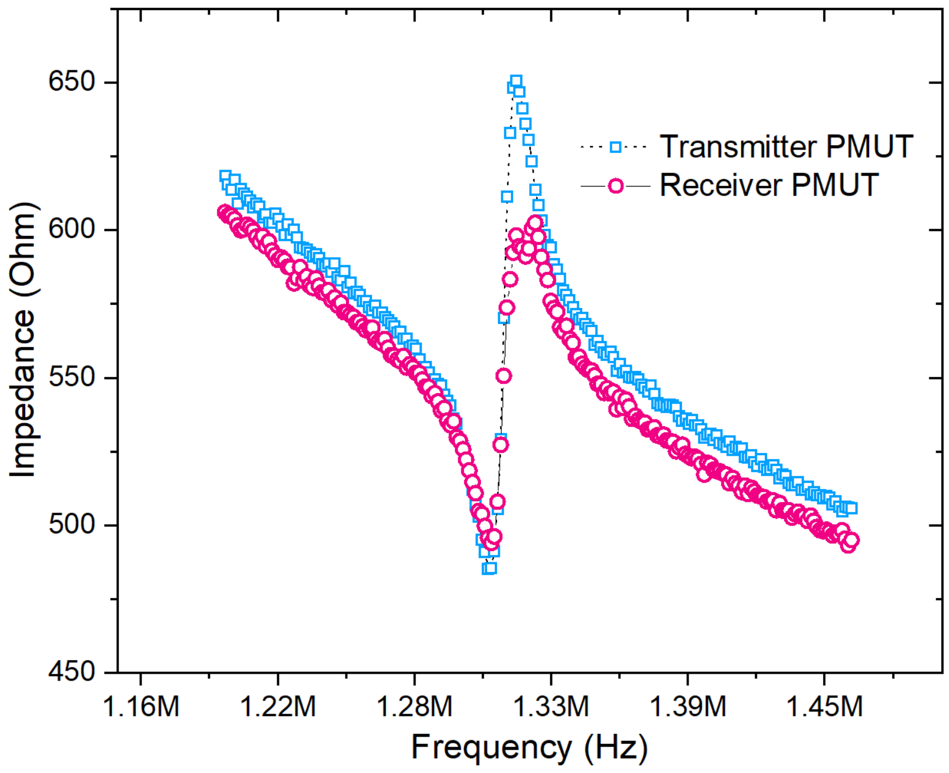

Supplementary Figure S6: Electrical impedance spectra of a typical transmitter and receiver PMUT used in the manuscript.

energy, represented as $Z=R+jX$ (where j is the imaginary unit). For acoustic resonators, the impedance can significantly vary with frequency, reaching a minimum at resonance where the device is most efficient at energy conversion. The resonance frequency (f_0) of an acoustic resonator, where it exhibits maximum energy storage relative to energy loss, is given by:

$$f_0 = \frac{1}{2\pi\sqrt{LC}}$$

With *L* representing the inductance and *C* the capacitance of the resonator. Understanding the electrical impedance of an acoustic resonator is essential for predicting how it interacts with

electrical circuits and ensuring optimal performance in its application. Additionally, the impedance spectra can also be used to characterize the effective electromechanical coupling coefficient (k_{eff}^2) which quantifies the efficiency of energy conversion between electrical and mechanical forms in piezoelectric devices, including acoustic resonators [43]–[45]. It is closely related to the resonant (f_r) and antiresonant (f_a) frequencies of the resonator. The resonant frequency is where the device shows maximum mechanical vibration and minimum electrical impedance, while the antiresonant frequency is where the mechanical vibration is suppressed, and the electrical impedance peaks. The relationship between k_{eff}^2, fr, and fa can be described by the following equation:

$$k_{eff}^2 = \frac{f_a^2 - f_r^2}{f_a^2}$$

This equation shows that k_{eff}^2 is directly proportional to the difference in the squares of the antiresonant and resonant frequencies, normalized by the square of the antiresonant frequency. This relationship is crucial for designing and optimizing piezoelectric devices, as it allows engineers to estimate the electromechanical coupling efficiency from the resonant and antiresonant frequencies, which are relatively easy to measure. High k_{eff}^2 values indicate efficient energy conversion, which is desirable for many applications, including sensors, actuators, and transducers.

F. PMUT Element Response Characterization

Before proceeding with making the clip device for the RWC measurement, it is important to know the functioning of all the cells in the PMUT element as described in Fig. S1. There are two sets of characterization which is being reported. The first set is the distribution of the resonant frequency of each of 49 (7 x 7) PMUT cells, and the second characterization is the deflection sensitivity at the resonance of each of 49 (7 x 7) PMUT cells. The LDV results

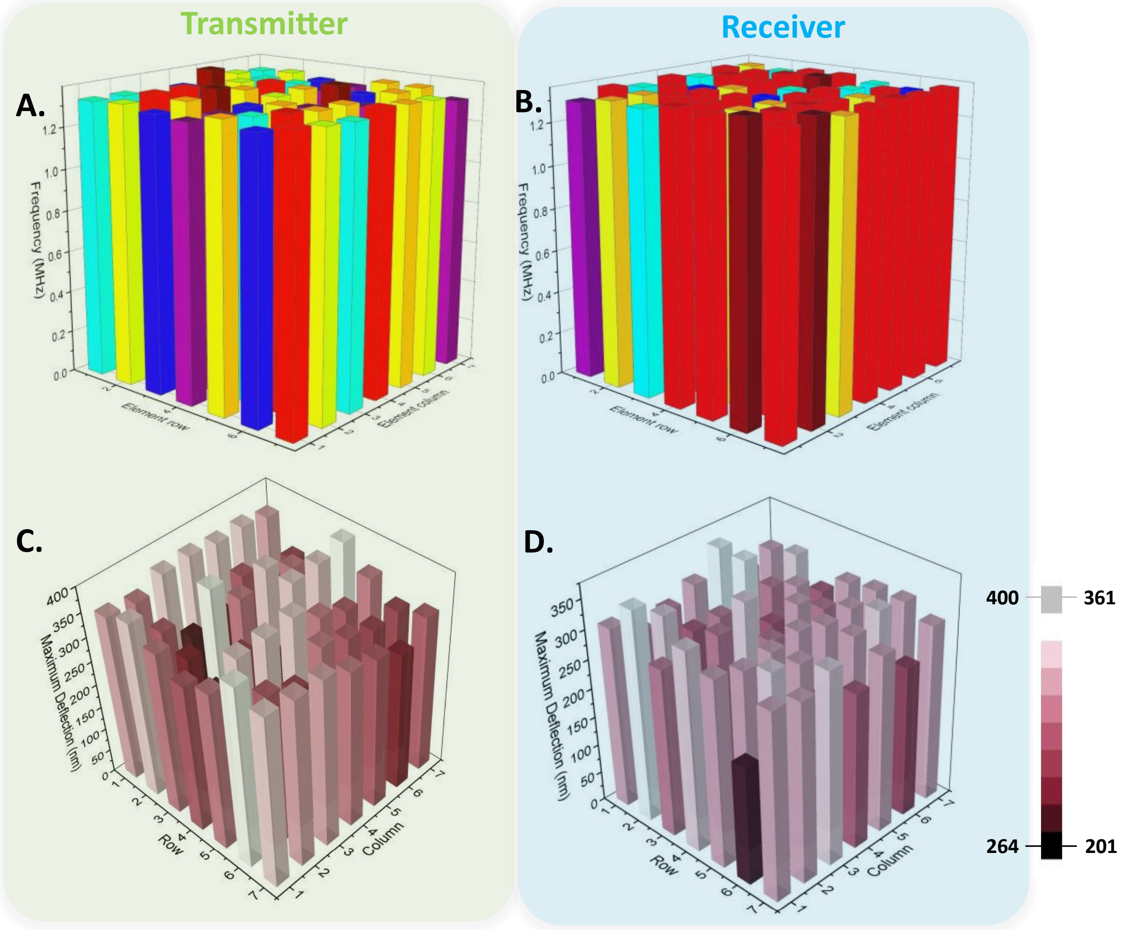

Supplementary Figure S7: PMUT element vibration response characterization: A. Frequency response of the transmitter element. B. Frequency response of the receiver element. C. Deflection sensitivity of the transmitter element. D. Deflection sensitivity of the receiver element.

suggest the variation in resonant frequency in the either transmitter or the receiver is from 1.3 to 1.39 MHz, which is 6.9%. Variation in the resonant frequency can be mainly due to four factors: (a) variation in the device dimension due to the etching/releasing process non-uniformity, (b) variation in the boundary condition at the individual device level due to formation of variable undercuts, (c) variation in membrane thickness due to non-uniform deposition of thin film layers in particular the piezoelectric thin film layer while sputtering and

(d) variation in the interlayer stresses developed as a result of high temperature processes non uniformly across the membrane. The elements investigated in this work show slight variation in the resonant frequency which is mostly due to the slight variations in the fabrication factors as mentioned previously. In the second set of characterization for the deflection sensitivity, the transmitter element presents the deflection of its cells in the range of 264-400 nm/V with a variation of 51.5% and the receiver element presents the deflection of its cells in the range of 201-361 nm/V with a variation of 79.6%. If the resonant frequencies do not vary much, the variation in the deflection sensitivities mostly arise due to the differences in the energy loss mechanism in the various cells, which inherently might be related to the variation in the acoustic cavity created by the silicon migration process. Further fabrication tape outs and studies are required to understand the problem better and is currently out of the scope of work. Further optimization of the silicon migration process is required in order to have consistent deflection sensitivities.

G. Process Flow for Making the PMUT-Leaf-PMUT Clip

Next, we describe the process steps for building the PMUT-Leaf-PMUT (PLP) clip architecture which can measure the RWC noninvasively from a leaf.

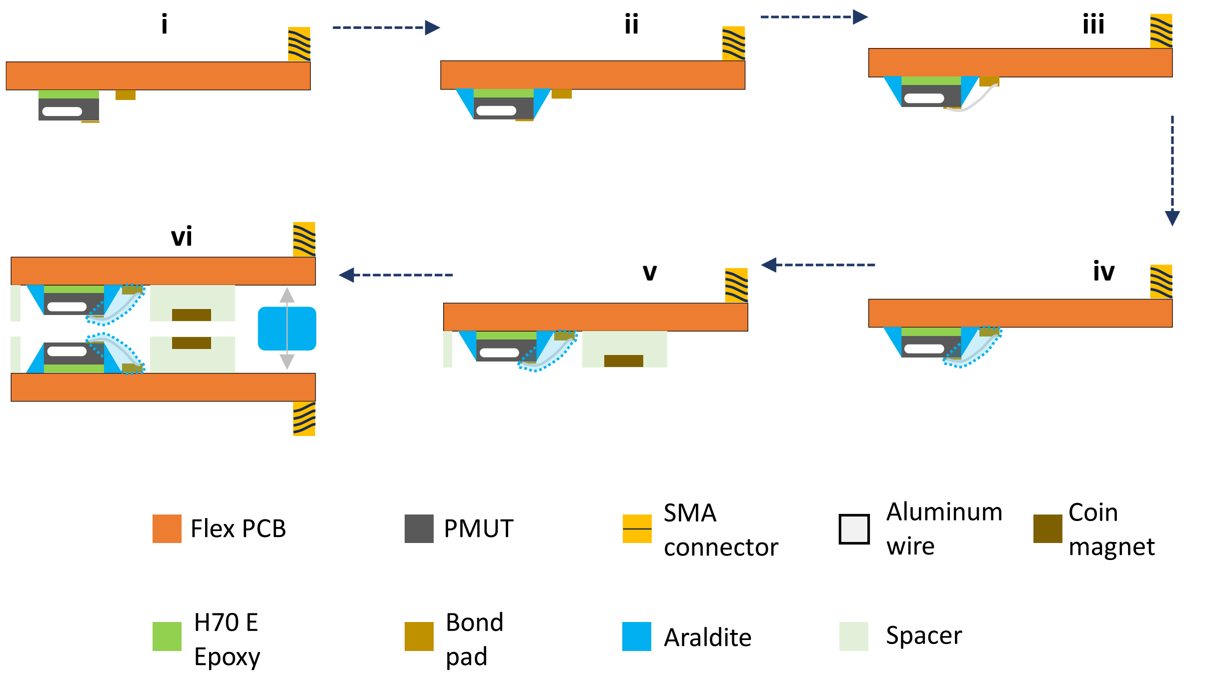

Supplementary Figure S8: Fabrication process flow for the PMUT-Leaf-PMUT clip

i. Designing and manufacturing of the flexible PCB. SMA connectors are soldered to the fabricated PCB. PMUT is then die bonded using the H70 E two-part epoxy from EpoTek Inc.

ii: PMUT's perimeter is secured and levelled with Crystal 2K (two-part epoxy) from Araldite Inc. This is done in order to provide a support for the future wirebond which needs to rest on the epoxy's surface. Also, the epoxy used needs to be a non-conductor of electricity so as to serve as an electrical isolation.

iii: Wire bonding of PMUT to the PCB pad. Wedge bonding is carried out for the process. Parameters such as bonding time of 10 ms, bonding force of 26 gf, and ultrasonic power of 256 mW is used to achieve successful bonding.

iv: Bond wire reinforcement using the Crystal 2K epoxy. Manual reinforcement of the wirebond is carried on by using a custom-made needle probe.

v: Bonding of the 3D printed spacer to the flex PCBs containing the coin magnet in designated position.

vi: Alignment of the PCBs and bonding at the distal end using the Crystal 2K epoxy. A constant pressure is applied at the bonding edge for 10 minutes, followed by release.

H. Spacer Optimization

The spacer forms one of the most crucial components of the PLP device architecture and had

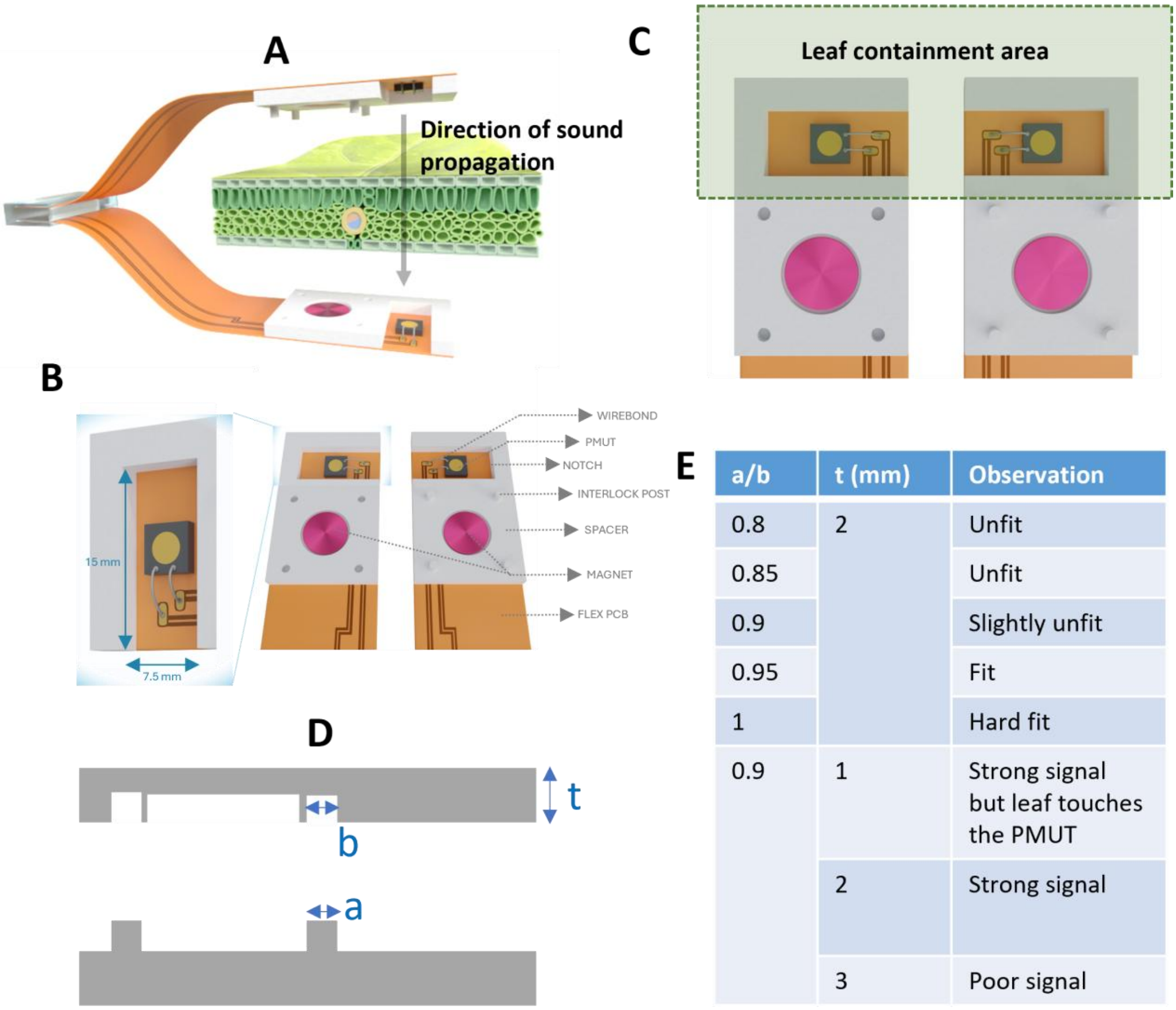

a/b	t (mm)	Observation
0.8	2	Unfit
0.85		Unfit
0.9		Slightly unfit
0.95		Fit
1		Hard fit
0.9	1	Strong signal but leaf touches the PMUT
	2	Strong signal
	3	Poor signal

Supplementary Figure S9: Spacer optimization for efficient performance of the PLP. A. The picture depicting the PLP architecture used in the contribution. B. Zoomed in spacer package constituting the PLP showing all the important parts, with a zoomed-in inset of the notched part containing the PMUTs C. Leaf containment area: the portion where the leaf blade must rest while taking the measurements. D. Cross section of the spacers showing the elements to which dimensions needed to be optimized: a – the diameter of the interlock's post, b- the diameter of the interlock's cavity, and c – thickness of the spacer. Interlocks are used to prevent the clip ends from sliding against each other. E. The optimized values for the post, cavity, and the thickness. It is observed when the a/b ratio is 0.95, the fit is relatively snug, thereby preventing any unnecessary sliding. For the thickness of the spacers, 1 mm thickness produces strong receive signal, but the leaf surfaces might touch the PMUTs creating undesirable non repeatable results, 3 mm of thickness results in weaker received signal. The optimized thickness was then found to be 2 mm.

to be optimized thoroughly for efficient functioning of the device. The spacer as depicted in

the Fig. S9B. contains two important parts: (a) the coin magnet as the central occupant and (b) the interlocks having 4 male plugs on the transmitter side and respective 4 female sockets on the receiver side evenly arranged surrounding the coin magnet. Other mentionable parts are the flex PCB, notch containing the PMUT dies, and the wire bonds connecting the die to the Flex PCB connections. A zoomed-in inset of the notched region is being provided. The opening measures as 7.5 mm x 15 mm. The function of the coin magnets is to assist in the leaf attachment and then hold the device in place in the direction of sound propagation. This helps in the achievement of level one attachment. The next level of attachment is achieved by the male-female interlocks which prevent any further motion in the direction normal to the sound propagation thereby resisting any external torque and differential sliding. Now, it is important to note that the leaf needs to be contained in the area specified in the Fig. S9C. and must not bypass the interlocks into centre of the device. In case the leaf blade bypasses inside, there is a significant chance of the damage to the leaf tissues. In addition, the tissue material might seep into the female interlock and change the dimension of the fit, thereby causing malfunction of the PLP. Thus, care must be taken every time the PLP is attached onto the leaf thereby ensuring accurate placement of the leaf blade into the clip assembly into the designated space. To develop interlocks with accurate fit with best device voltage readout, the male and female plug and socket dimensions and the overall thickness of the spacer had to be optimized (Fig. S9D). Several 3D printed trials to achieve designated male to female dimension ratios were tried, along with several combinations of the spacer thickness. The optimized ratio value was found to be 0.95 as reported in the table. Varying the spacer thickness led to certain conclusions. If the spacer was too thin, with thickness being 1 mm, the output signal strength was high, but the leaf touched the PMUT's surface. This is not acceptable since if the leaf blade encounters the PMUT's surface that could: (a) make the PMUT's surface dirty by leaving unwanted residues, (b) may interfere with the wirebonds thereby weakening the bond strength, and (c) fall directly on the PMUT's membrane and interfere with the generation of acoustics. Moving to higher spacer thickness such as 3 mm, weakens the signal too much to be visible. Thus, 2mm of thickness was chose, which could give a considerably strong signal along with ensuring a safe working distance between the leaf and the PMUT surface.

I. PLP's Time Domain Acoustic Response

To evaluate the PLP's functionality, we conducted experiments focused on sound-voltage transmission. It was important to observe the clip's time domain acoustic signature, which is in essence the burst response of the receiver PMUT element when the transmitter transmits a burst of ultrasound at its resonance. To achieve such result, a prior experiment had to be conducted to understand the clip's overall acoustic response in the frequency domain and thereby find the resonant frequency of the quasi-closed system comprising PMUT dies, flex PCB, and 3D printed spacers.

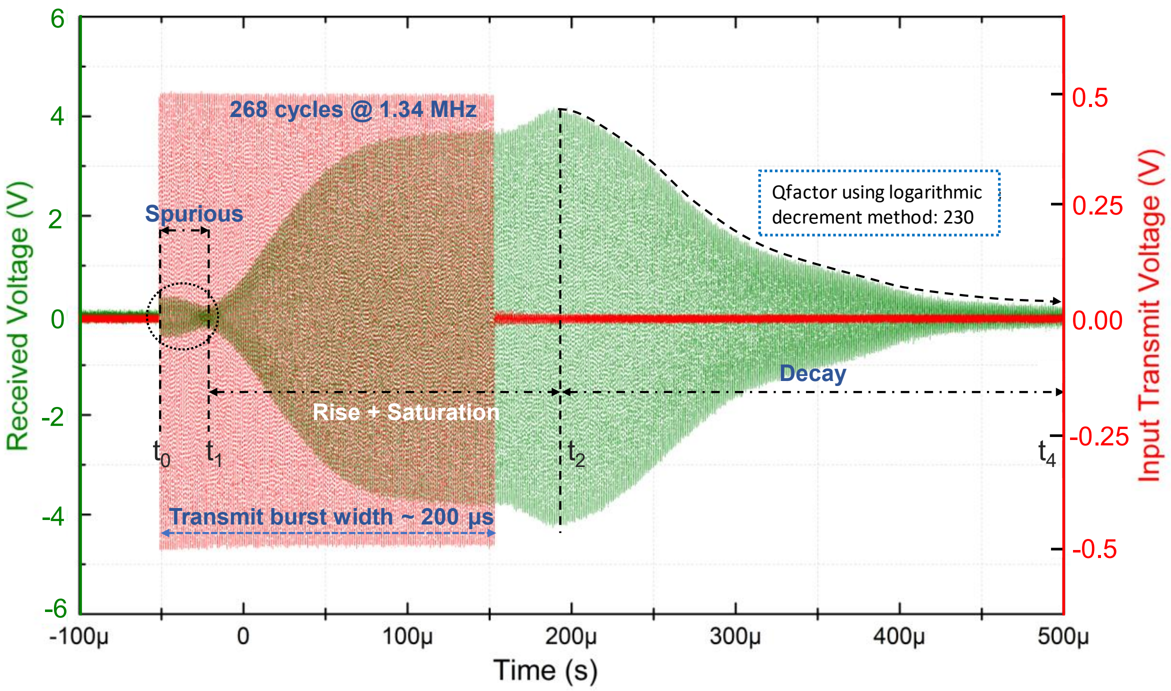

Supplementary Figure S10: Time domain acoustic response of the PLP transceiver device.

In the first experiment the frequency of the transmitter was swept from 1.25 MHz to 1.4 MHz, operated at 1 V AC sine, and the peak-to-peak sine amplitude as observed from the receiver was recorded after charge amplification (10 V/pC, HQA-15M-10T, Femto Inc.) The resonant plot revealed dual peaks at 1.32 MHz and 1.34 MHz, the latter being higher amplitude, due to superposed ultrasound waves and multiple reflections. Figure 2E. in the manuscript depicts such a plot.

After knowing the resonant peaks of the acoustic system, in the second experiment, we operated the transmitter at the sine burst of 1.34 MHz (746 ns cycle period); we determined the necessary cycles to reach resonance at 268, corresponding to a 200 µs transmit burst. The time-

domain response as can be seen from the figure can be divided into three parts: (a) t0-t1 shows spurious ultrasound transmission through the spacer, since solid objects transmit sound faster than air; (b) t1-t2 (205 μs) is the system's resonance attainment time in combination with saturation and the onset of the second peak; t2-t4 (309 μs) represents signal decay, which follows a logarithmic decay pattern. The logarithmic decrement method is a widely used technique for determining the quality factor (Q-factor) of a resonator, which is a measure of the resonator's efficiency in storing energy. The Q-factor is particularly important in the context of oscillatory systems, such as mechanical pendulums or electrical circuits, where it quantifies the damping of oscillations. Mathematically, the logarithmic decrement, δ, is defined as the natural logarithm of the ratio of successive amplitudes of a damped oscillator. Given two successive peak amplitudes, A_n and A_{n+1}, in the same direction, can be expressed as:

$$\delta = ln\left(\frac{A_n}{A_{n+1}}\right)$$

The quality factor Q of the resonator is inversely proportional to the logarithmic decrement and is given by the formula:

$$Q = \frac{\pi}{\delta\sqrt{1 - \frac{\delta^2}{4\pi^2}}}$$

For small values of δ, where the underdamped condition is assumed, and the term $\frac{\delta^2}{4\pi^2}$ becomes negligible, simplifying the expression for Q to:

$$Q \approx \frac{\pi}{\delta}$$

This method of determining the Q-factor is particularly useful for its simplicity and the minimal equipment required. By measuring the amplitudes of successive oscillations, one can easily compute the logarithmic decrement and, subsequently, the Q-factor. The system's quality factor, estimated using the logarithmic decrement method, is 230, indicating a low loss, sharp frequency response.

J. Finite Element Simulation (Part I): PMUT's Vibroacoustic Response

In order to understand the device physics better and to verify the accurate functioning of the PMUT, it is important to visualize through finite element simulations the vibroacoustic

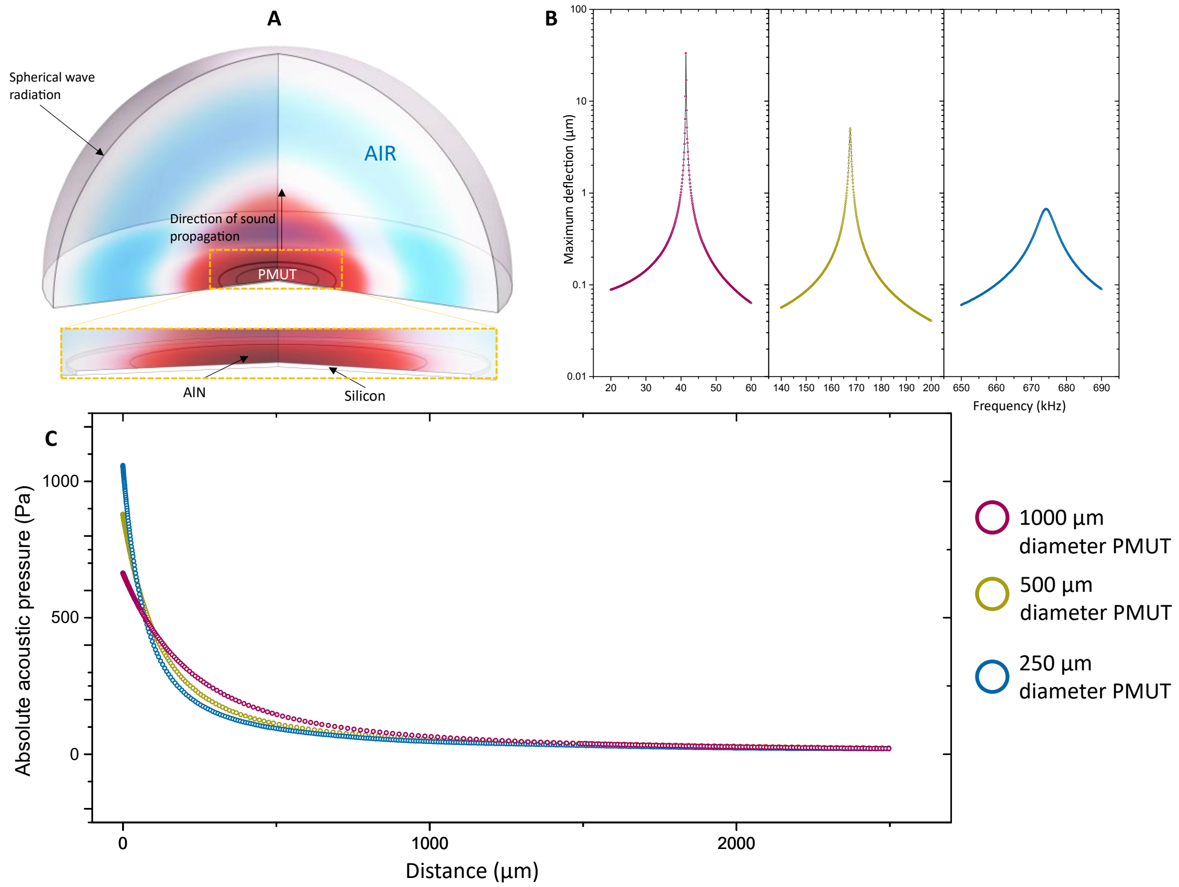

Supplementary Figure S11: COMSOL Multiphysics simulation of PMUT's response. A. 2D axis-symmetric half-space model showing PMUT vibrating in air. A spherical wave radiation acoustic boundary condition has been used to describe the acoustic boundary, which prevents the formation of acoustic reflections. B. Deflection frequency response function as obtained from PMUTs of 3 different diameters corresponding to 3 different frequencies. A frequency of 40 kHz, 170 kHz and 675 kHz is obtained for PMUTs having diameters 1000 µm, 500 µm and 250 µm respectively. C. Acoustic attenuation from PMUTs of three different sizes with the 250 µm PMUT having the highest value of acoustic pressure initially, while falling off fast as compared to the 1000 µm PMUT. After 1 mm of distance, either of them is observed to have similar pressure values.

response of a single PMUT cell at various dimensions [46], [47]. For simplification, 2 D axis-symmetric model was used to capture the functioning of the PMUT used in this project. Although the PMUT cells used in the project are square in dimension, and accepting the fact that this difference would create differences in the values obtained as results, it is strongly believed that the physics and the physical observations will still be the same, thereby generating

consistent patterns in the data. The model geometry as selected was an infinite half-space with the infinity condition enforced through the spherical wave radiation boundary condition. Air was used as the acoustic medium, and the PMUT cell investigated had Aluminium Nitride (AlN) instead of ScAlN. The simulation was performed for three different dimensions: 1000 µm, 500 µm, and 250 µm respectively. The deflection frequency response as obtained from the simulation is plotted in Fig. S11B, and a frequency of 40 kHz, 170 kHz and 675 kHz was observed for PMUTs having diameters of 1000 µm, 500 µm, and 250 µm respectively. Next, the absolute acoustic pressure as obtained from the three different PMUTs was plotted with respect to the distance and the plot so obtained in shown in Fig. S11C. The 250 µm PMUT had the highest value of acoustic pressure initially, however the sound fell off faster as compared to the 1000 µm PMUT. After 1 mm of distance, either of them is observed to have similar pressure values.

K. Finite Element Simulation (Part II): PMUT-Air-PMUT

In order to understand the working of the final PMUT-Leaf-PMUT (PLP) device, it is first important to understand the behaviour of the device without the leaf inside. Such device will be having air in between the transmitter and the receiver PMUT dies and has been named PMUT-Air-PMUT. The acoustic space in this simulation is a complete infinite sphere containing air all through. The infinity of the sphere is idealized by the spherical wave radiation boundary condition. The gap between the transmitter and the receiver is 'g' and the sound propagates fom the transmitter to the receiver as depicted in the Fig. S12A. The frequency of the PMUTs is adjusted by adjusting the diameter and by keeping other factors such as the stack thickness and the stress distribution constant. The deflection frequency response for both the transmitter and the receiver is depicted in Fig. S12B, when the separation gap between the cells is kept at 2mm. The transmitter cell shows a higher deflection than the receiver PMUT since it is directly actuated using the AC sine voltage, whereas the receiver vibrates and deflects indirectly from the sound generated by the transmitter PMUT, which reaches the receiver after a significant amount of loss in the passage air. An acoustic contour map is also plotted which shows the pressure distribution in the air space as depicted in Fig. S12C. The plot shows acoustic pressure concentration on the transmitter side and creation of soft standing waves due to the formation of the partial rigid acoustic boundary condition arising due to the rigidness of the PMUT device, thereby generating sound reflections. We then compare the deflection as felt by the receiver PMUT with the voltage received when the transmitter is actuated at 1V sine at 1.3 MHz. We vary g from 100 μm to 2 mm and observe the maximum deflection and the V_{TRx} to fall off following exponential decay as expected from the theory. After g = 1000 μm, the V_{TRx} value saturates in the receiver cell without much further variation. As a concluding part of the simulation, we contour-plot the pressure distribution in the air-space as the transmitter cell is moved away from the receiver cell (see Fig. S12E). The standing waves start to appear in the space from g = 600 μm, before which the wave in mostly constricted between the two PMUT cells. At g = 600 μm, the waves find a leaky path to spread in the air-space mostly due to acoustic diffraction.

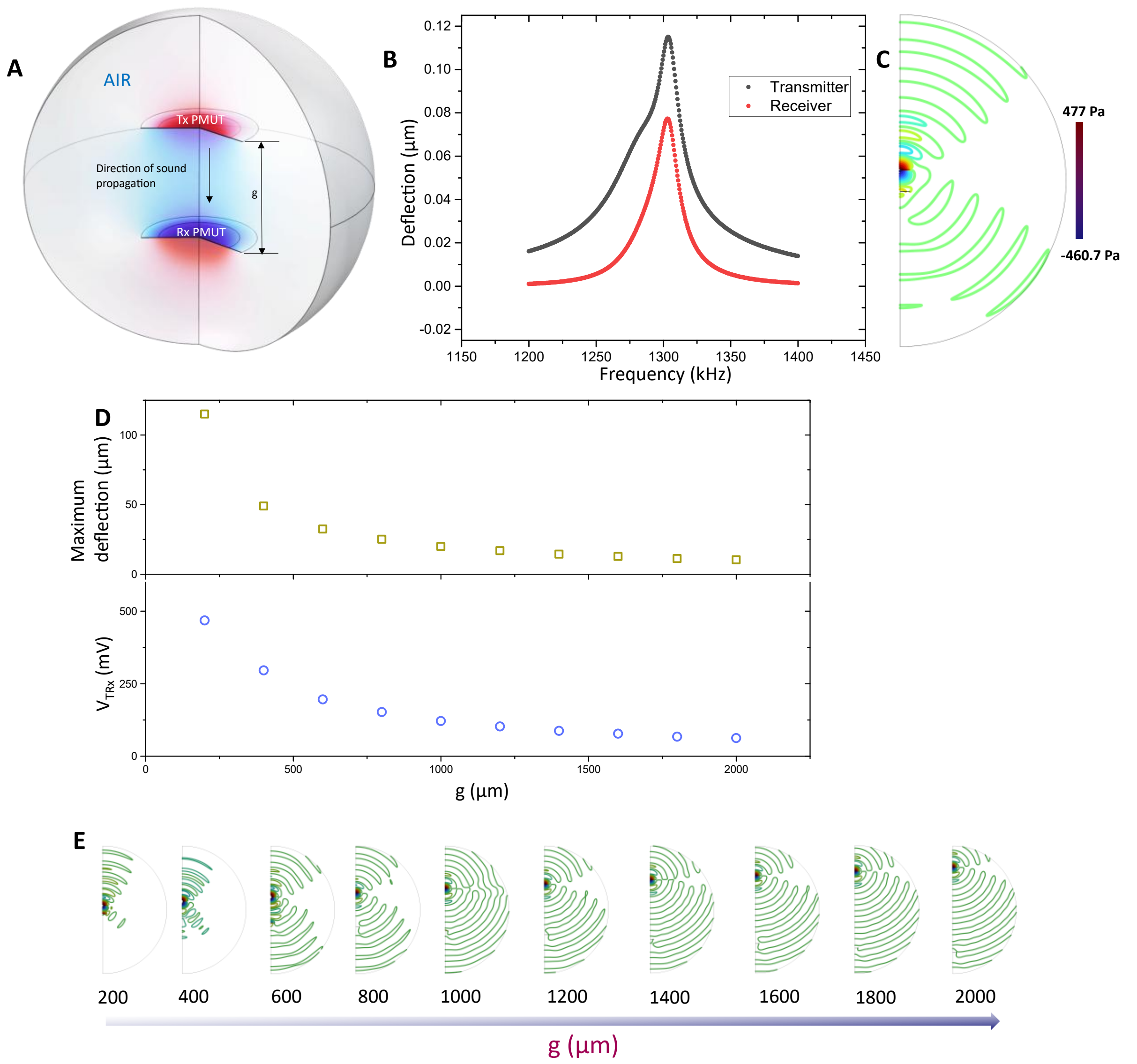

Supplementary Figure S12: COMSOL Multiphysics simulation of a PMUT-space-PMUT, wherein the space is air for this particular simulation. A. 2D axis-symmetric full-space spherical model showing PMUTs vibrating in air. The transmit PMUT is in top, whereas the bottom PMUT is in bottom. The gap between the two PMUTs is marked as *g*. A spherical wave radiation boundary condition is maintained at the boundary of the space to prevent unwanted reflections. B. Frequency response in terms of PMUT deflection showing both the transmitter and receiver's behaviour. The resonant frequency is noted at 1.3 MHz which is also the frequency of the PMUTs used for the experimental purposes. The transmitter peak is a little distorted following the creation of partial standing waves in the PMUT-space-PMUT architecture. C. Acoustic field contour map of the PMUT-space-PMUT at the resonance. D. Maximum deflection and transceived voltage as observed from the receiver PMUT as the g is varied from 100 µm to 2 mm. An un-realistic deflection indicates the linearity of the model which does not include the non-linear response of the PMUT after a certain membrane deflection. E. Acoustic contour map of the space as the gap is increased from 200 µm to 2 mm.

L. Finite Element Simulation (Part III): PMUT-Leaf-PMUT

In the final part of the simulation, a complete architecture containing two PMUT cells, with leaf in between was created, in an infinite airspace (Fig. S13A). This part of the simulation gives is the nearest to the actual PLP experiments and gives a feel of the sound propagation physics from the air to the leaf to the air, and proposes several important observations as described in the text below. The maximum acoustic impedance as reported by for a leaf [47], [48] can be 0.5 MRayl and was adopted to define the leaf acoustically. The ultrasound attenuation coefficient was fixed at 350 Np/m following the work of Farinas et al. [48]. The simulation can be further divided into three parts: (a) effect of the variation of leaf area to the airspace acoustics, when the leaf thickness was held constant (b) effect of the variation of leaf area to the airspace acoustics, when the leaf thickness was held constant, and (c) effect of the variation of leaf impedance to the airspace acoustics, when the leaf are and thickness were held constant. In the first subpart, (Fig. S13B) three different leaf section dimensions were considered: 200 µm, 700 µm and 1400 µm respectively, as shown in Fig. S13. The 3D acoustic field, the 2D field contour and the V_{TRx} frequency response were plotted to understand the effect. The acoustic field developed for each dimension case was significantly different. When the leaf dimension is small, acoustics reaching to the receiver is observed to be strong. This acoustics can be mostly divided into two parts: the direct acoustics from the transmitter which penetrates the leaf and reaches the receiver, and the part which diffracts around the leaf structure to reach the receiver. As the leaf dimension gets bigger, the direct acoustic intensity coming out from the leaf decreases since the acoustic pressure strength gets distributed in a bigger area. The diffracted acoustics strength decreases as well, following the wrap around against a larger geometry. The receiver PMUT cell being of constant size, feels lesser sound pressure owing to the areal spread of sound in the leaf. This observation is quantified by the V_{TRx} frequency response showing a maximum of 950 mV for the 200 µm and decaying down to 5.5 mV for the 1400 µm diameter leaf, thereby decreasing nearly 170 times. This concludes the fact that leaf dimension to the transmitter/receiver PMUT ratio plays a strong role in deciding the acoustic response of the device. In the second subpart, the thickness of the leaf section was allowed to vary from 10 µm to 200 µm, by keeping the diameter constant at 1000 µm. The 3D acoustic field pattern along with the 2D pressure contour was almost the same all the leaf thicknesses with the only difference in the value of the pressure as felt by the receiver PMUT cell. The decrease in the felt pressure is mostly due to the attenuation of the sound wave inside the leaf cross-section owing to the thickness factor involved in the exponential material

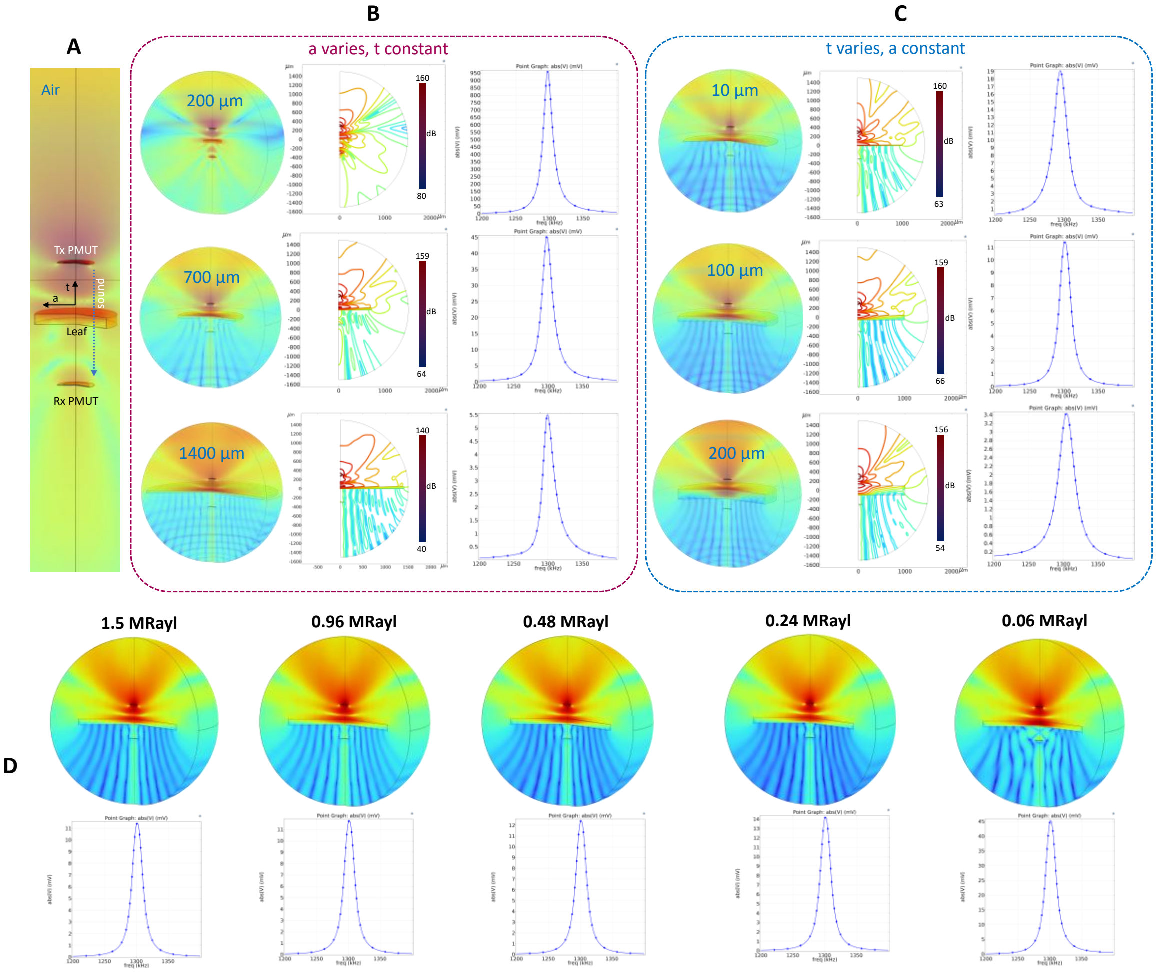

Supplementary Figure S13: COMSOL Multiphysics simulation of a PMUT-space-PMUT, wherein the space is air along with a piece of leaf. The leaf is being modelled as a unified material having only one layer for the sake of simplicity. The change in the water content of the leaf is mimicked by varying the acoustic impedance of the material from that of water to that of air. A. A snapshot of the 2D axis-symmetric full-space spherical model showing the PMUTs, the leaf and the air. The leaf section is assumed to be cylindrical with a radius of *a* and a thickness *t*. B. Acoustic response as a is varied, keeping the t constant, with a assuming values: 200 µm, 700 µm, 1400 µm. Maximum voltage as seen from the receiver is also seen to decrease from 950 mV to 5.5 mV, with the trend being nonlinear. C. Acoustic response as t is varied, keeping the *a* constant, with t assuming values: 10 µm, 100 µm, 200 µm. Maximum voltage as seen from the receiver is also seen to decrease from 19 mV to 3.4 mV. Acoustic response as the impedance is varied from 1.5 MRayl to 0.06 MRayl, with substantial amount of sound passing at 0.06 MRayl.

absorption decay. This observation is quantified by the V_{TRx} frequency response showing a maximum of 19 mV for the 10 µm and decaying down to 3.4 mV for the 200 µm thick leaf, thereby decreasing nearly 6 times. This concludes that the effect of thickness in influencing the acoustics inside the device is weaker as compared to the leaf dimension. In the last part of the simulation, impedance of the leaf was changed from 1.5 MRayl to 0.06 MRayl, in order to

observe the effects of change in the tissue properties due to water loss. From 1.5 MRayl to 0.24 MRayl, there was no variation in the acoustic field or the maximum receiver deflection frequency response, while from 0.24 MRayl to 0.06 Mayl, substantial variation in the maximum deflection values, thereby increasing nearly 3 times (see Fig. S13D).

M. Setup Used to Detect RWC using PLP

The instrumentation for RWC detection using PLP comprised of a function generator from Agilent Inc., a charge amplifier from Femto Inc., an oscilloscope from Tektronix Inc., and a

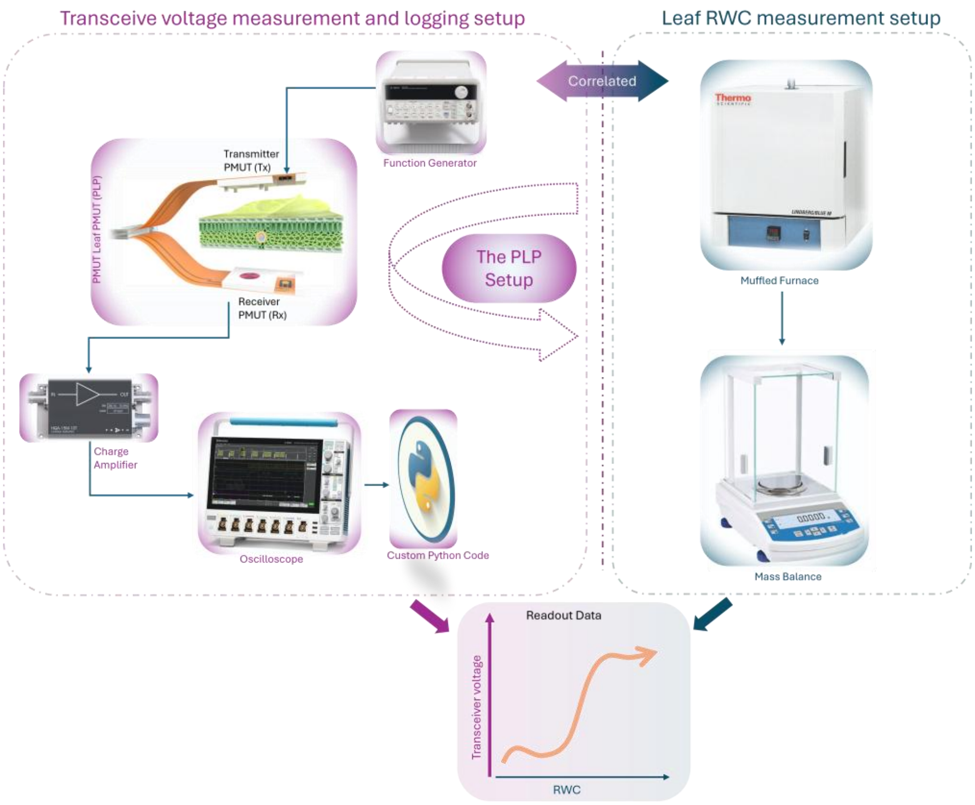

Supplementary Figure S14: Setup used to detect RWC using PLP

custom-made python code to interface the receiving end such as the charge amplifier and oscilloscope for data logging and storage. The function generator generates 1 V AC at the fundamental resonance of the transceiver, which creates ultrasound. The leaf/material of interest consequently interacts with the sound and through-transmits the wave accordingly, which is then received by the receiver. The piezoelectric layer in the PMUT generates time variant charge which is then amplified using a charge amplifier and fed into the oscilloscope. Next, a custom code is developed in python to record the peak-to-peak amplitude levels of the amplified signal and store it. A parallel correlated setup is created using the muffled furnace and mass balance to determine the RWC. The leaf of interest is heated for a fixed span of time and the mass measured via the mass balance and the voltage measured via the PLP setup.

Supplementary Note III: PLP Device on Leaf

This section describes the various additional aspects of the piezo-MEMS ultrasound device: the PMUT-Leaf-PMUT proposed in this work to sense leaf relative water content (RWC) [32], [33] when applied on detached leaves and on whole plant leaves. In particular, this section starts off by informing the readers about the various water content metrics used to quantify a leaf's health. Subsequently, the discussion compares the existing conventional RWC measuring technique most popularly and majorly used globally with our invented technique, thereby explaining clear advantages our technique offers to plant biologists and agriculturists. Next, we delve deeper into explaining the mechanism based on which PLP works, and finally conclude the section with one detached leaf experiment and another whole plant experiment performed using the PLP.

A. Leaf Water Content Metrics

Relative Water Content (RWC) is a widely used metric in plant physiology to quantify the water status of a leaf or plant tissue. It provides a measure of the water level in a sample relative to its fully hydrated and turgid state, offering insights into the plant's water stress status, hydration efficiency, and overall health. RWC is expressed mathematically as:

$$RWC = \frac{FW-DW}{TW-DW}\text{x}100$$

where:

FW is the Fresh Weight of the leaf or plant tissue, representing its weight immediately after being harvested.

DW is the Dry Weight, obtained after drying the tissue in an oven at a high temperature (usually around 60°C) for a period long enough to remove all water content, ensuring that only the dry matter remains.

TW is the Turgid Weight, which is measured after submerging the plant tissue in water for a sufficient period (often overnight), allowing it to absorb water until it reaches a fully turgid state, where no more water can be absorbed.

Other associated quantities used to express water levels in a leaf include:

Water Potential (Ψ): This is a comprehensive measure that represents the potential energy of water in a plant tissue relative to pure water under the same environmental conditions. Water

potential integrates various components, including osmotic potential (Ψ_s) and pressure potential (Ψ_p), to describe the driving force for water movement into or out of plant cells.

Osmotic Potential (Ψ_s): This quantifies the effect of solutes in the plant sap on the potential energy of water. A solution's osmotic potential is always negative, indicating that the presence of solutes decreases the water's potential energy compared to pure water.

Pressure Potential (Ψ_p): This represents the physical pressure on water within a plant cell, which can be either positive, in the case of turgor pressure in living cells, or negative, in the case of tension in xylem vessels. It is a critical factor in maintaining plant rigidity and driving water movement.

Water Saturation Deficit (WSD): This is another way to express water deficiency in plant tissue. It represents the difference between the amount of water a tissue can hold at full turgidity and its current water content. It is usually expressed as a percentage of the total water capacity:

$$WSD = \left(1 - \frac{FW-DW}{TW-DW}\right) x100$$

Understanding these quantities and their interactions is vital for studying plant-water relations, which can influence plant physiology, crop yield, and responses to environmental stresses.

<u>Why monitoring RWC is important?</u>

Water availability above ground in vegetative and seed forming stages must be tightly regulated, and having plant wearables can provide more management handles. Monitoring plant relative water content (RWC) is a key yet simple method for stress phenotyping, informing ideal irrigation practices and minimizing water wastage. RWC offers insights into plants' watering needs, growth stages, and overall health, responding to various environmental stresses like poor irrigation, saline soil, or extreme atmospheric conditions. However, current methods for RWC measurement mainly involve destructive sampling, hindering real-time plant water response studies. Therefore, developing instantaneous, non-invasive, low-cost, lightweight, plant wearable for RWC determination is crucial for both researchers in plant water physiology and agronomists evaluating water-related traits.

B. RWC Determination Techniques: Our Work's Novelty

In this section we compare the conventional method of finding the RWC for a particular leaf to the proposed method invented in this work: the generative-AI assisted PLP.

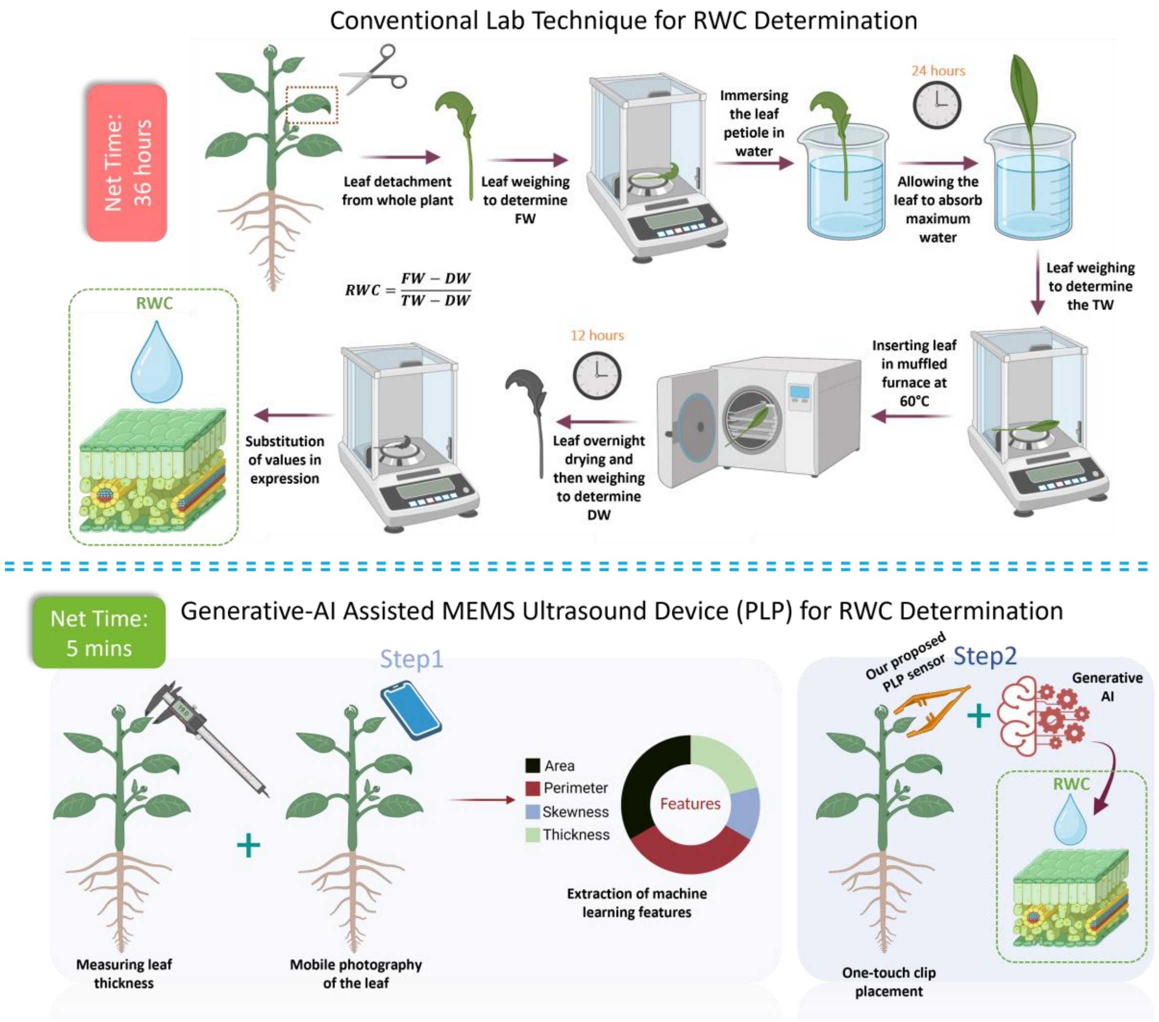

Supplementary Figure S15: Comparison of the traditional technique used to measure a leaf's RWC after detachment with the current invented generative-AI plus PLP technique to measure a leaf's RWC without the necessity of leaf detachment from whole plant.

Determining a leaf's RWC using the conventional method is a 36-hour process involving seven different steps. First, it starts with detaching the desired leaf having at least 2 inches of petiole off the plant. Second, the detached leaf is weighed in the mass balance to determine the fresh weight (FW) or the current weight, which contains information on the amount of water present in the leaf at the current state. Third, the leaf which might be a little wilted (assuming it is not at its 100% RWC or in other words not completely turgid) is then immersed in water. Care should be taken to immerse just the petiole in the water else it might suffocate the leaf. Fourth,

the leaf is allowed to absorb water from the petiole for the next 24 hours of time to make it fully turgid. Extra water is subsequently removed from the petiole by using air-jet and it is ensured the leaf plus petiole surface is completely dry. Any extra weight will add error to the

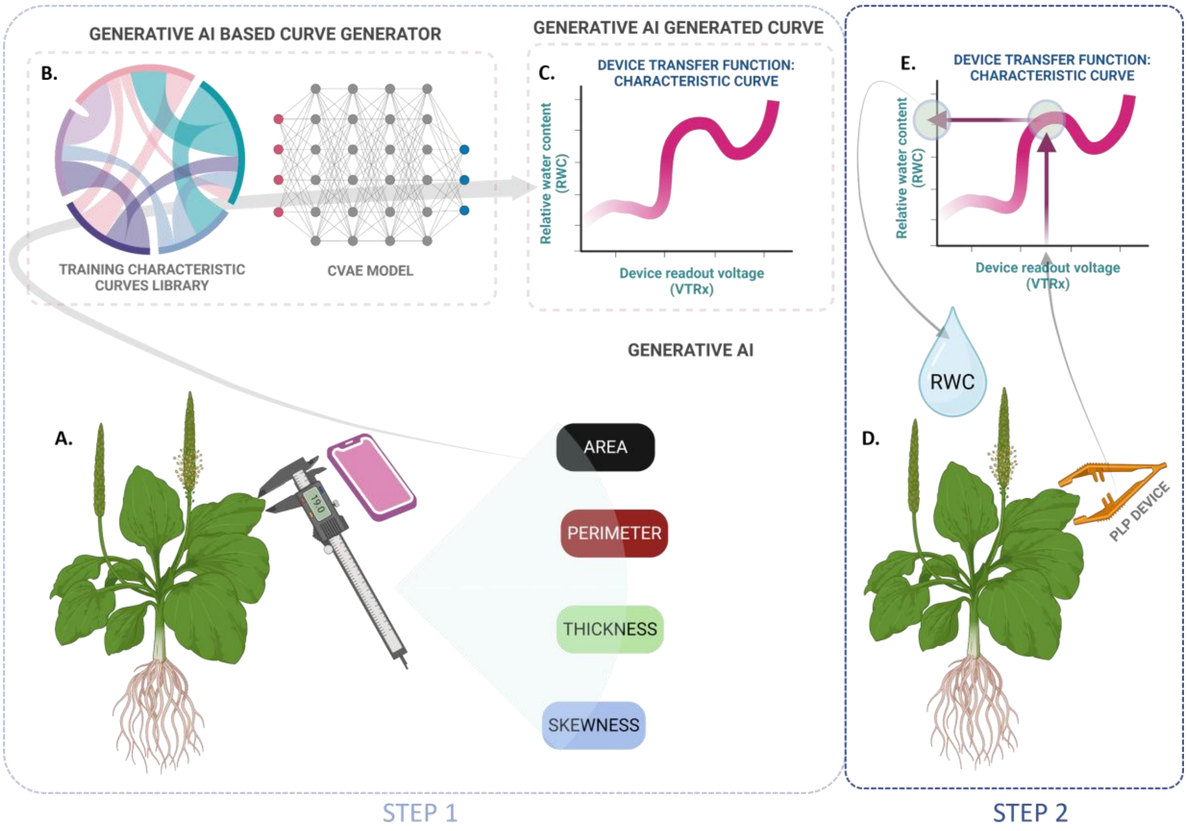

Supplementary Figure S16: Working of the generative-AI plus PLP device to detect a leaf's RWC in a shot. A. Step 1: Determination of the geometrical parameters of the desired leaf, which serves as the machine learning features. B. Step1: The parameter values are fed to the generative AI based curve generator. C. Step1: The curve generator then generated/predicts the characteristic curve based on its training. D. Step 2: One shot PLP attachment to the desired leaf to determine the received voltage V_{TRx}. E. Step 2: Referring to the characteristic curve to determine the value of the RWC.

accurate determination of the RWC. Fifth, the leaf is re-measured for its weight, thereby noting the turgid weight (TW), or in other words the weight when the leaf contains water to its fullest capacity. Sixth, the leaf is then inserted in a muffled furnace and heated overnight at 60°C for 12 hours. This ensures removal of all the water that would have been present in the leaf, thereby ensuring the leaf is dry. Seven, the leaf is remeasured for its weight, thereby noting the dry weight (DW), which is the weight of just the tissue component of the leaf without any inherent leaf water. Finally, the noted values are substituted in the RWC expression to determine the RWC of the leaf.

On the other hand, determining the leaf's RWC using the generative-AI assisted PLP device as shown in Fig. S14 is a 5-minute procedure involving two different steps. First the geometrical parameters of the desired leaf such as the area, perimeter, skewness, and thickness have to be determined. Determining the thickness requires usage of a vernier calliper while the other parameters can easily be determined using mobile photography. There are application already available in the market which can then directly conclude the parameter, or, even a dedicated simple software application can be developed. The second step is to attach the clip on the leaf blade, and the PLP will accurately determine the RWC, given the value of the available parameters.

In order to delve deeper into the working of the generative-AI assisted PLP device, the reader may refer Fig. S15, which is divided into two steps as previously described in Fig. S14. In step1, first the leaf of interest is targeted and various geometrical features such as the area, perimeter, skewness, and thickness are collected by using mobile photography and by using the vernier calliper (Fig. S15A). The parameter values so measured are then fed to the trained generative AI model: the convolution variational autoencoder (CVAE). The model is trained using a library of characteristic curves which are in essence the device transceived voltage (V_{TRx}) vs. the RWC, as obtained by investigating 200 leaves all having different collection of the geometrical parameters or features (Fig. S15B). Thus, the trained CVAE model works as the generative AI based curve generator (by curve we mean the nonlinear graph describing the relationship between RWC and V_{TRx}. Thus, the trained generative AI based curve generator generates the desired curve which is specific to the geometrical features of the targeted leaf and strongly depends on the parameter values provided by the user (as measured using the mobile photography and vernier calliper) as shown in Fig. S15C. This concludes the step 1, wherein the prime motto is to determine the characteristic curve for a particular leaf under consideration by using the user fed geometrical leaf parameters in conjunction to the generative AI based characteristic curve generator. In step 2, the PLP device is first used by one-shot attachment to the leaf of interest to determine the device readout voltage (V_{TRx}) as shown in Fig. S15D. Next, the characteristic curve generated by the generative AI curve generator is referred to determine the RWC value corresponding to the V_{TRx} value obtained using the device. This concludes the step 2, thereby reporting the user the value of the RWC for the leaf of interest.

The advantages the AI-assisted PLP sensor offer towards measuring RWC over the conventional lab technique are as follows: (a) The number of steps required to evaluate RWC

of a particular leaf is reduced from 7 to 2; (b) the time required to evaluate RWC of a particular leaf is reduced from 36 hours (2160 minutes) to 5 minutes thereby decreasing the time by 99.8%; (c) leaves need not be detached from the plants in order to measure RWC, in other words using PLP the user can directly attach the device on any leaf in the whole plant and in a shot get the value of the RWC of the particular leaf; (d) the plant wearable nature of the PLP helps in determining the RWC dynamically, continuously online, thereby making it possible to track a plant's health through a leaf without requiring any external human engagement; (e) it is possible even miniaturize the overall device dimension owing to the CMOS compatibility nature of the device making process, thereby having the entire device with the readout electronics sitting on a leaf; (f) CMOS compatibility also ensures wireless connectivity thereby making AIOT (Artificial Internet of things) empowerment possible, and even realizing sensor networks on several leaves and several plants, thereby making water stress management in-field more easy and efficient.

C. PMUT-Leaf-PMUT Working Principle

In order to understand the working principle of the PMUT-Leaf-PMUT (PLP) device, it is important to first understand the important leaf layer components in a leaf cross section, since the PLP device works on the through transmission of sound through various leaf layers. In other words, sound will interact with each one of these layers and be absorbed by the components. Presence of water inside these components highly change the acoustic properties such as the impedance and attenuation which will have a strong effect on the amplitude of the final wave emanating from the leaf after through transmission.

The paragraph below portrays the seven important components of a leaf as visualized from the cross section. This information is relevant since it is believed that each of the biological layers the leaf is comprised of will be having different acoustic properties such as the density, speed of sound, ultrasound attenuation coefficient, and elastic modulus as discussed by Farinas et al. [48]. (a) Cuticle: The leaf cuticle is a waxy layer that conserves water by reducing transpiration, offers protection against environmental damage, and aids in regulating gas exchange. It also shields the plant from UV radiation and, with its reflective surface, helps to prevent overheating. (b) Epidermis: The plant epidermis serves as a protective barrier against water loss, pathogens, and mechanical injury. It facilitates gas exchange via stomata and sometimes contains trichomes for added defence and to reduce transpiration, playing a crucial role in overall plant health and homeostasis. (c) Palisade mesophyll: The palisade mesophyll, located beneath the epidermis in leaves, consists of densely packed, columnar cells rich in chloroplasts, making it the primary site for photosynthesis. These cells absorb light efficiently due to their arrangement and high chlorophyll content, driving the conversion of carbon dioxide and water into glucose and oxygen. The mesophyll's structure also facilitates gas exchange, vital for photosynthetic activity and plant respiration. (d) Air void: Air voids in leaves, found within the spongy mesophyll, facilitate efficient gas exchange. They provide spaces for oxygen and carbon dioxide to diffuse directly to and from the photosynthetic cells and the stomata, playing a critical role in the plant's respiration and photosynthesis processes. (e) Spongy mesophyll: The spongy mesophyll, located beneath the palisade layer in a leaf, is composed of loosely arranged cells with intercellular spaces or air voids. These spaces allow for the circulation and exchange of gases (carbon dioxide, oxygen, and water vapor) necessary for photosynthesis and

respiration. Its structure aids in the diffusion of gases to and from the stomata and the

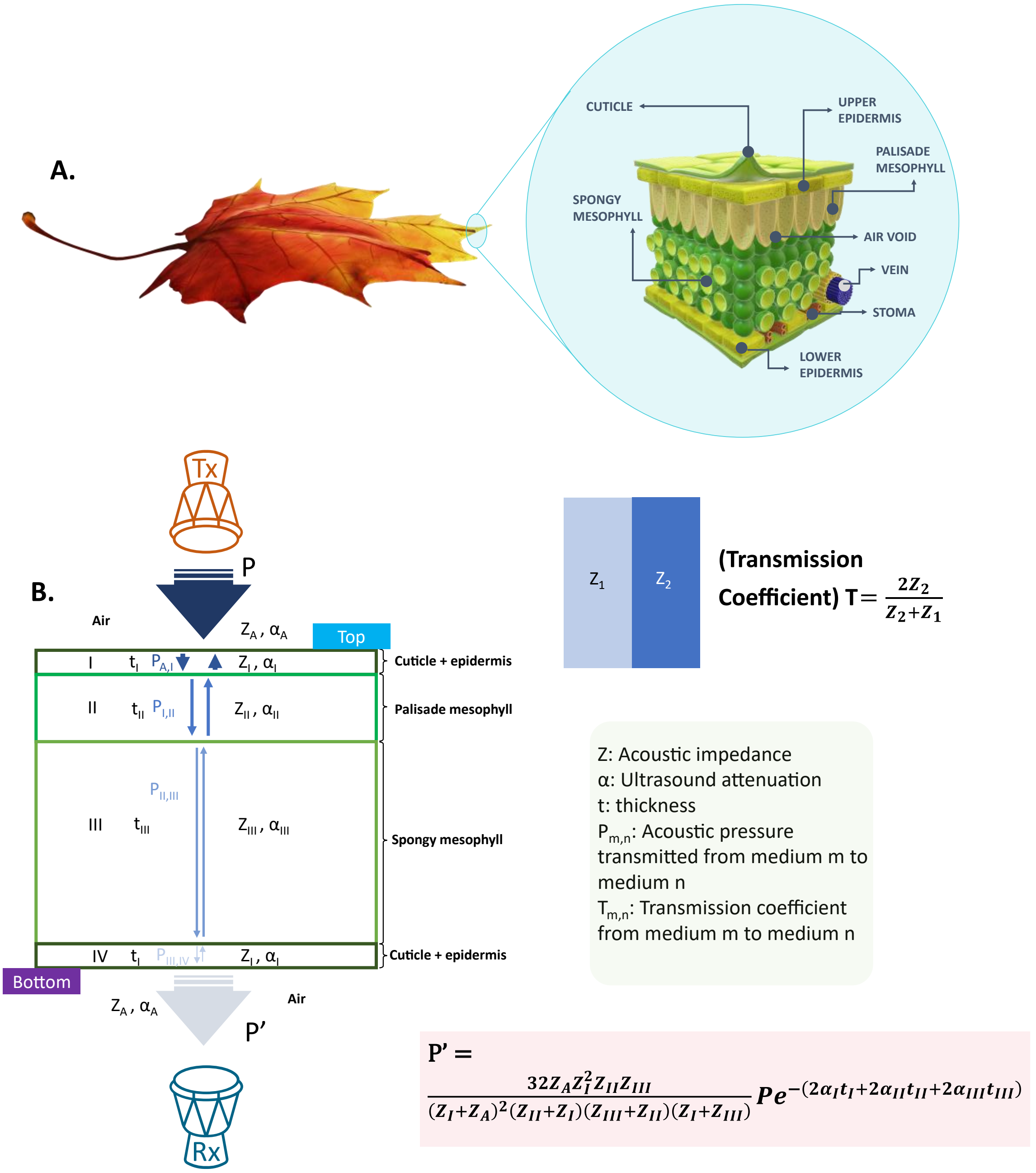

Supplementary Figure S17: PLP Working principle. A. Components of a leaf cross section. B. Mechanics of sound transport from the transmitter PMUT to the receiver PMUT through the leaf cross section

photosynthetic cells and helps in regulating the leaf's internal humidity and temperature. (f) Vein: Veins in a leaf, part of the vascular system, are essential for transporting water and minerals from the roots to the photosynthetic tissues via the xylem, and for distributing the synthesized sugars from the leaves to other parts of the plant through the phloem. They also provide structural support, maintaining leaf shape, and facilitate the quick distribution of

nutrients and water within the leaf, which is critical for efficient photosynthesis and overall plant health. (g) Stomata: Stomata are microscopic pores on the leaf surface that regulate gas exchange and transpiration. They open to allow the intake of carbon dioxide necessary for photosynthesis and release oxygen as a byproduct. Concurrently, they facilitate the loss of water vapor in a process called transpiration, which drives the uptake of water and nutrients from the roots. Stomata also play a role in cooling the plant through evaporative cooling and in maintaining homeostasis by balancing water and gas levels.

Next, we delve deeper into deriving the output acoustic pressure emitted from the leaf as a function of the input acoustic pressure and various leaf material constants such as the acoustic impedance and the ultrasound attenuation in each leaf layers. The leaf is approximated to consist of several distinct layers out of which three may have greater vibroacoustic significance [48]: the cuticle + epidermis, palisade mesophyll, and spongy mesophyll. Each of these layers has unique physical properties that affect how sound waves pass through them, specifically in terms of acoustic impedance, ultrasound attenuation, and layer thickness. Here's a breakdown of the key concepts and processes described:

Acoustic impedance (Z) of a medium [49] is a measure of how much resistance an acoustic wave faces as it travels through that medium. It is defined by the product of the medium's density (ρ) and the speed of sound (c) within it, $Z = \rho c$. Each layer in the leaf has a different acoustic impedance due to variations in density and composition, affecting how sound waves are transmitted and reflected at interfaces between layers.

Ultrasound attenuation refers to the decrease in the intensity of the ultrasound wave as it propagates through a medium. This attenuation can be due to absorption, scattering, and reflection within the medium. It is influenced by the medium's properties and the frequency of the ultrasound wave. The palisade mesophyll and spongy mesophyll, with their distinct structures and water content, will attenuate the sound wave differently.

Transmission and Reflection As the incident sound wave (with magnitude P) encounters interfaces between different layers, part of the wave is transmitted into the next layer, while part is reflected back. The amount of transmission and reflection depends on the acoustic impedances of the adjoining layers. The greater the difference in impedance, the more reflection occurs. This process of transmission and reflection continues as the wave passes through each layer of the leaf.

The final output pressure (*P'*) emitted from the bottom surface of the leaf is a function of the acoustic impedances, ultrasound attenuation of the respective layers, their thicknesses, and the initial pressure (*P*). This means that *P'* is influenced by how much the sound wave is attenuated and reflected within the leaf's structure. The water content of the leaf affects its acoustic impedance and ultrasound attenuation. As the water content changes, so do these properties, altering how the ultrasound wave is transmitted and reflected. Since water has a significant impact on the leaf's density and the speed of sound within it, variations in water content can lead to noticeable changes in the measured *P'*. The final output pressure (*P'*) exerts a force on the surface of a receive Piezoelectric Micromachined Ultrasonic Transducer (PMUT), which converts this pressure into a voltage readout (V_{TRx}). This voltage readout contains indirect information about the leaf's relative water content because the changes in *P'* reflect changes in the leaf's acoustic properties due to variations in water content.

Derivation of the output pressure P' which emanates from the bottom surface of the leaf when the top surface is exerted by an initial acoustic pressure P:

Let us assume P falls on the top surface of the leaf at a normal incidence. It then gets divided into two parts: the transmitted part and the reflected part. The part which enters into the 1st layer of the leaf is $P_{A,I}$ and the corresponding transmission coefficient is $T_{A,I}$

$$P_{A,I} = T_{A,I}P$$

$$T_{A,I} = \frac{2Z_I}{Z_I+Z_A}$$

Next, the resulting sound from the layer I enters layer II with a pressure value of $P_{I,II}$ and corresponding transmission coefficient of $T_{I,II}$

$$P_{I,II} = T_{I,II}P_{A,I}e^{-\alpha_I t_I}$$

$$T_{I,II} = \frac{2Z_{II}}{Z_{II}+Z_I}$$

Next, the resulting sound from the layer II enters layer III with a pressure value of $P_{II,III}$ and corresponding transmission coefficient of $T_{II,III}$

$$P_{II,III} = T_{II,III}P_{I,II}e^{-\alpha_{II} t_{II}}$$

$$T_{II,III} = \frac{2Z_{III}}{Z_{III}+Z_{II}}$$

Next, the resulting sound from the layer III enters layer IV with a pressure value of $P_{III,IV}$ and corresponding transmission coefficient of $T_{III,IV}$

$$P_{III,IV} = T_{III,IV} P_{II,III} e^{-\alpha_{III} t_{III}}$$

$$T_{III,IV} = \frac{2Z_I}{Z_I + Z_{III}}$$

Next, the resulting sound from the layer IV leaves out in the air with a pressure value of *P'* and corresponding transmission coefficient of $T_{IV,A}$

$$P' = T_{IV,A} P_{III,IV} e^{-\alpha_I t_I}$$

$$T_{IV,A} = \frac{2Z_A}{Z_A + Z_I}$$

Making the necessary substitutions, yields the final expression for the *P'* in terms of *P* as:

$$\frac{32 Z_A Z_I^2 Z_{II} Z_{III}}{(Z_I + Z_A)^2 (Z_{II} + Z_I)(Z_{III} + Z_{II})(Z_I + Z_{III})} P e^{-(2\alpha_I t_I + 2\alpha_{II} t_{II} + 2\alpha_{III} t_{III})}$$

D. Detached Leaf Experiment Using PLP

In order to demonstrate the capability of the generative AI-assisted PLP device as a standalone sensor capable of determining leaf RWC through the device voltage readout, we attached our device in detached leaves having 3 different areas. Before beginning the experiment, each one of the leaves after detachment was pre-processed by dipping the attached petiole in water for 24 hours to gain complete turgidity. After the 24 hours dip, the leaves were uniformly dried. Then, each leaf was then heated at intervals of 1.5 minutes for 60°C in a muffled furnace for

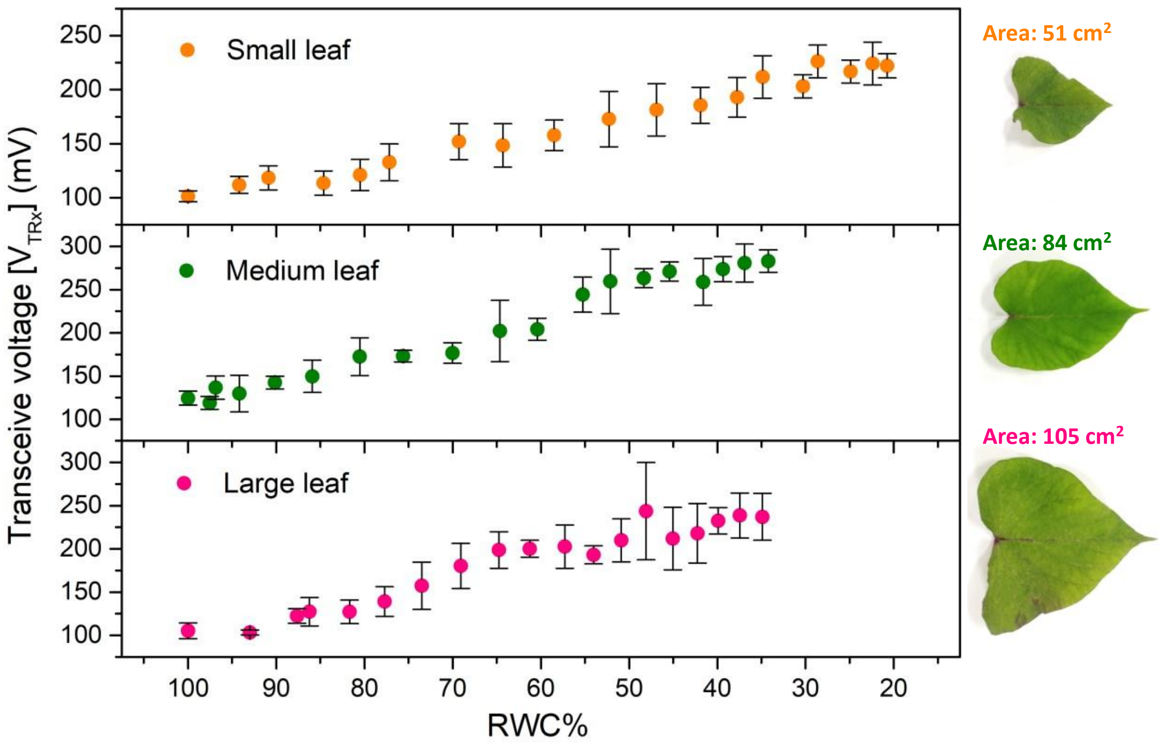

Supplementary Figure S18: Detached leaf experiment using the PLP device depicting three different readout voltage (V_{TRx})-RWC relationship for three different sizes of Japanese sweet potato leaves.

19 times and readings such as the mass and the readout voltage from the PLP device were taken for 19 times in between the heating intervals. As can be observed from the Fig. S16, the device readout voltage V_{TRx} followed an incremental trend with the RWC values, which implied that V_{TRx} and RWC holds a certain kind of nonlinear relationship. With increase in the RWC value, V_{TRx} seemed to increase nonlinearly. This observation is accompanied with another observation: each of the curve pattern for the 3 leaves is significantly different from one another. This implies the water loss mechanics in terms of the V_{TRx} -RWC relationship is unique to leaves of different sizes. For example, the medium and big leaves lost water slowly as compared to the smaller leaf as captured by the V_{TRx} -RWC relation. This paves a clear path of

using the machine learning in order to understand and learn the various intricate patterns connecting the V_{TRx}-RWC relation and the leaf geometrical features such as the area, perimeter, thickness and skewness.

E. Whole Plant Experiment Using PLP

An experiment was conducted for 24 hours with PLP mounted on five different plants such as: Begonia, Peace Lily, Polka Dot, Sweet Potato and Coleus, to observe the difference in obtained

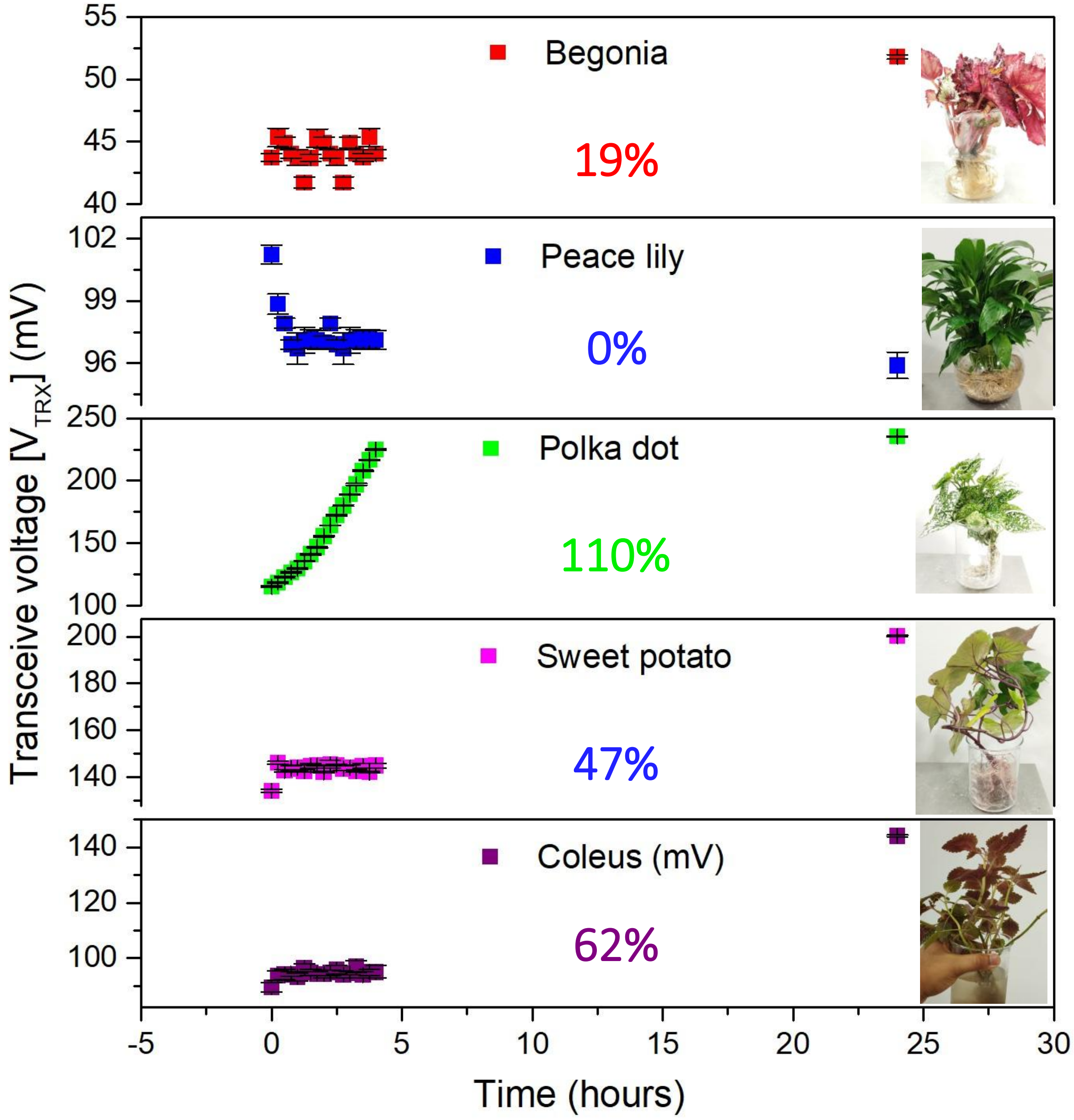

Supplementary Figure S19: Whole plant experiment using the PLP device, depicting different water loss curves for different plants having different drought sensitivity.

PLP clip readings when the device is mounted on plants having different phenotypes. To start with, all the plants were detached from the soil and the roots washed to remove any excess particles of soil. Then the plants were hydrated by directly placing the roots in well-watered

containers as shown in the Figure. After 12 hours of water absorption, when the plants were in their fully turgid condition, they were taken out of the water containers, and dried for 15 minutes using air shower gun. The plants were then kept in dry containers. PLP was attached to one of the healthy leaves and the respective plant was allowed to lose water. Ambient air conditioning was held constant to ensure non-variability in the experimental environment. The V_{TRx} values were manually recorded for the first 4 hours of the onset of experiment and then again at the 24th hour.

There are some observations from the data so recorded: Firstly, this experiment demonstrates the capability of the PLP device to remain attached to a particular leaf for a long period of time (24 hours) and continuously measure the through transmission of sound through the voltage readout, making it suitable for longer term plant health monitoring through leaves. Secondly, leaves of all plants demonstrated different ranges of the V_{TRx} values, with Begonia starting off from 40 mV to 50 mV (~19%), Peace Lily 102 mV to 96 mV (0%), Polka Dot starting from 100 mV to 250 mV (~110%), Sweet Potato starting from 140 mV to 200 mV (~47%) and Coleus starting from 90 mV to 150 mV (~62%). This demonstrates the specificity of the PLP to a plant's RWC. In other words, this shows the capability of the PLP device to understand the difference between a drought sensitive plant (polka dot) as compared to a drought in-sensitive plant (peace lily). This capability in future can farther be tapped to detect and classify drought sensitive and insensitive plants as well. Thirdly, it demonstrates the difference in water loss pattern for different plants as recorded by the PLP. Peace Lily showed almost 0% water loss while Polka Dot showed 110% loss. This indeed confirms the underlying difference between the plants having different genotypes, which can successfully be captured by using the PLP device.

F. Growth Chamber Used for Experiment

Growth chamber [50], [51] from Jiupo Inc. used for the controlled environment experiments.

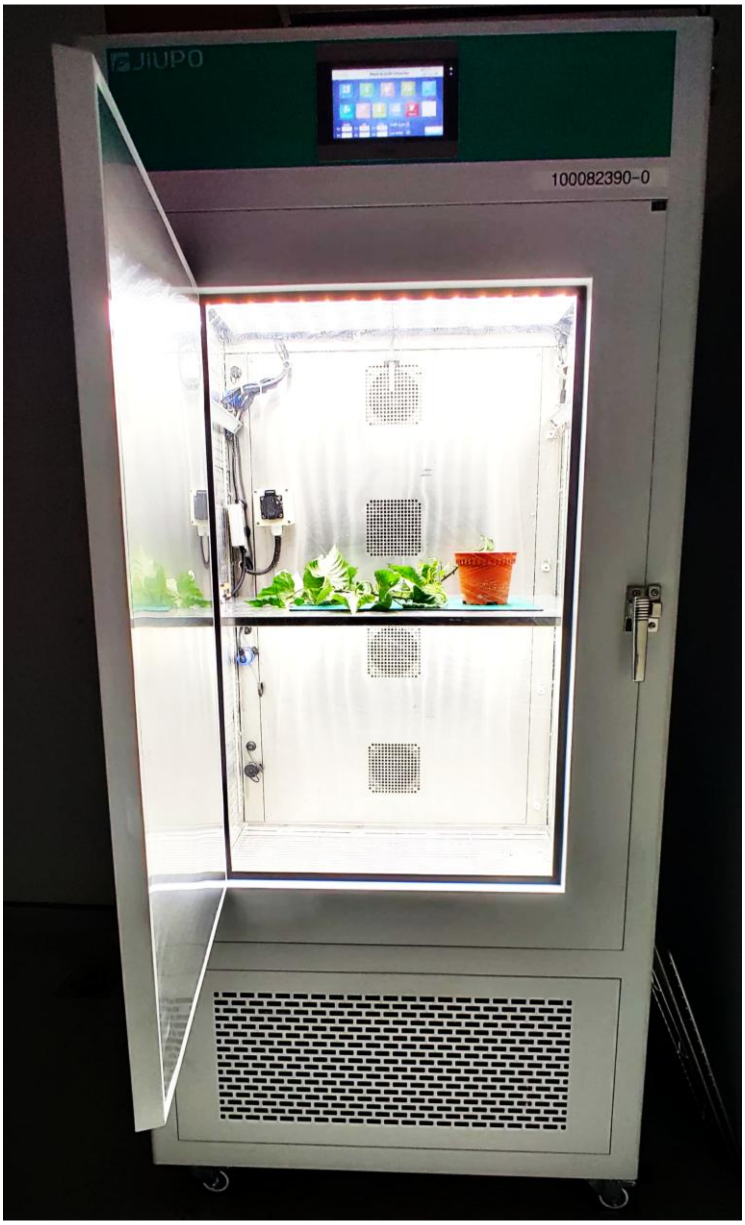

Supplementary Figure S20: Growth chamber from Jiupo Inc. used for the controlled environment experiments.

Environmental variables such as temperature, humidity levels and light intensity can be accurately controlled in the chamber. Any temperature levels from 20C to 45C can be selected, any humidity levels from 30% to 100% can be selected, and any light intensity levels from 0 A.U. to 1000 A.U. can be selected. This is added on by precise irrigation facility. The use of the growth chamber allows to maintain a strict vigil on the environmental variables, which if changed changes experimental outcomes drastically.

Supplementary Note IV: Machine Learning with PLP on Plants

This section describes the various additional aspects of the machine learning used in conjunction with the PLP device, in particular it educates the readers on the need of using the generative AI in order to solve the problem of the RWC determination using the PLP through transmission of sound device.

A. Cultivars of sweet potato plant used in the project

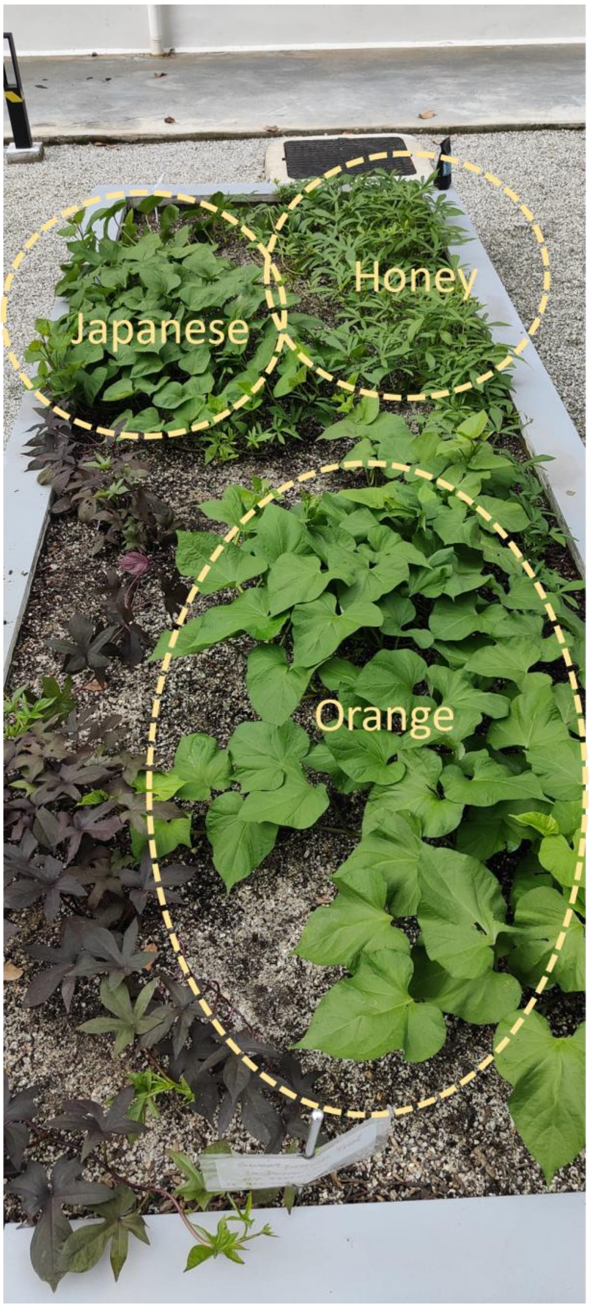

Supplementary Figure S21: Three different cultivars of sweet potato plant: Honey, Japanese and the Orange growing in the sun.

We have used sweet potato as our target plant in this project to prove the capability of the generative AI-assisted PLP device to determine leaf RWC. One of the reasons to use the sweet potato plant is its immense agricultural relevance in terms of the parts (leaves and tubers) of sweet potato consumed as a crop. Sweet potatoes are a nutritional powerhouse, celebrated for their myriad health benefits. Rich in beta-carotene, which the body converts into vitamin A, they support vision, immune function, and skin health. Their high fiber content aids in digestion and promotes a feeling of fullness, which can be beneficial for weight management. Sweet potatoes are also a good source of vitamins C and B6, potassium, and manganese, contributing to heart health and antioxidant protection. Moreover, the presence of anthocyanins, particularly in purple sweet potatoes, adds to their antioxidant properties, potentially reducing inflammation and lowering the risk of chronic diseases such as heart disease and cancer. With a low glycemic index, sweet potatoes provide a steady source of energy, making them an excellent choice for blood sugar regulation. This versatile plant, with its sweet flavor and nutritional benefits, is a valuable addition to a healthy diet. The second reason to use the sweet potato plant is its relatively moderate sensitivity to environmental stimulus such as heat, humidity and light illumination, which is particularly important to us since plants sensitive to such conditions help us demonstrate the applicability of using the PLP device as a continuous wearable plant health monitor within a relatively short span of time. Fig. S20 depicts three different cultivars of sweet potato plant: Honey, Japanese and the Orange growing in the sun. As is visible from the picture all the three varieties are different in terms of their phenotypes. For example, the leaves of the cultivars differ greatly in their geometry and sizes. There are also differences in the total number leaves each plant contain. An adult plant belonging to the Honey cultivar may contain nearly twice the number of leaves as compared to the other two cultivars.

B. Reason for using generative model such as CVAE as the deep learning algorithm

The general use cases of all the learning algorithms are portrayed in the figure. As seen artificial intelligence [52]–[55] can be divided into three sub-branches such as machine learning, which is further comprised of a subset called deep learning [56]–[58] which furthermore can be branched out to the generative AI [59]–[61].

Non-generative deep learning models focus on recognizing patterns, classifying data, and

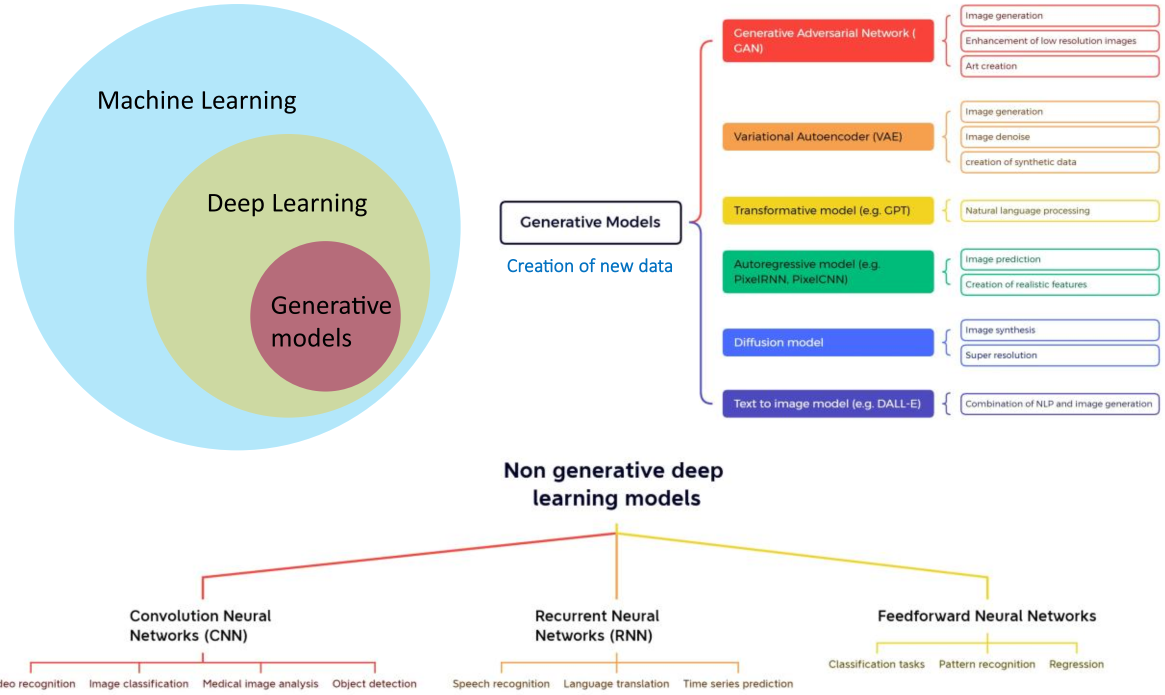

Supplementary Figure S22: Primary classification of machine learning into various comprising categories such as deep learning and generative learning (which is a subset of deep learning), and the reason behind using the generative model for project.

making predictions based on input data, rather than generating new data instances. Here's a brief description of three primary non-generative models: Convolutional Neural Networks (CNNs) [62], Recurrent Neural Networks (RNNs) [63], and Feedforward Neural Networks (FFNNs) [64], [65]:

Convolutional Neural Networks (CNNs): CNNs are specialized in processing data with a grid-like topology, such as images. A CNN automatically and adaptively learns spatial hierarchies of features from input images. The architecture of a CNN typically consists of a series of convolutional layers for feature extraction, followed by pooling layers for dimensionality reduction, and fully connected layers for classification. CNNs are widely used in image and

video recognition, image classification, medical image analysis, and other auto-vision tasks due to their ability to capture spatial relationships in data.

Recurrent Neural Networks (RNNs): RNNs are designed to recognize sequences of data, such as time series, speech, or text. Unlike feedforward neural networks, RNNs have connections that form directed cycles, allowing information to persist by looping back into the network. This makes RNNs capable of exhibiting temporal dynamic behaviour and processing inputs of variable length. RNNs are particularly useful for applications such as speech recognition, language modelling, and translation, where the sequence and context of the input data are crucial for accurate predictions. However, RNNs can struggle with long-term dependencies due to issues like vanishing and exploding gradients, leading to the development of more advanced variants like LSTM (Long Short-Term Memory) and GRU (Gated Recurrent Unit) networks.

Feedforward Neural Networks (FFNNs): FFNNs, also known as Multilayer Perceptrons (MLPs), are the simplest type of artificial neural network architecture. In a FFNN, information moves in only one direction—forward—from the input nodes, through the hidden nodes (if any), and finally to the output nodes. There are no cycles or loops in the network. FFNNs are well-suited for a wide range of applications, from simple binary classification to complex pattern recognition tasks. They are particularly effective for problems where the input features are independent of each other, but they lack the ability to handle sequential data or capture temporal/spatial relationships as effectively as CNNs or RNNs.

On the other hand, generative AI encompasses models and techniques designed to generate new data instances that resemble the training data, enabling the creation of content like images, text, and music that can be indistinguishable from real, human-generated content. Here's an overview of various generative AI models:

Generative Adversarial Networks (GANs): GANs [66], [67] consist of two neural networks, a generator and a discriminator, which are trained simultaneously through adversarial processes. The generator creates data instances (e.g., images), while the discriminator evaluates them against real data, trying to distinguish genuine instances from the fakes. Over time, the generator improves, producing increasingly realistic data. GANs are widely used for image generation, style transfer, and more.

Variational Autoencoders (VAEs): VAEs [8] are a type of autoencoder that generates high-dimensional data by learning a latent space representation of the input data. They work by

encoding input data into a latent (hidden) space representation and then decoding this representation back to the original data space. VAEs are particularly good at generating new instances of data in a controlled manner, as they allow for the manipulation of the latent space to vary the outputs. They're used in image generation, facial reconstruction, and more.

Generative Pre-trained Transformer (GPT): GPT models, developed by OpenAI, are autoregressive language models used for generating human-like text. They predict the probability of a sequence of words and generate text one word at a time, based on the words that have come before. GPT models have been utilized for a variety of applications, including chatbots, content creation, and even code generation, showcasing remarkable versatility and capacity for understanding and generating human language.

Autoregressive Models: Autoregressive models predict future values in a sequence by learning from past values. They are a foundational approach in time series forecasting and have been adapted for use in generative models for text, audio, and image data. In generative applications, they sequentially produce pixels, words, or audio samples, conditioning each new piece of content on the previously generated content.

Diffusion Models: Diffusion models generate data by gradually transforming a random noise distribution into a data distribution through a process that involves progressively denoising the content. This approach has been especially successful in generating high-quality images and has been extended to audio and video generation. The process involves a forward phase that adds noise to real data until it becomes pure noise, and a reverse phase that learns to reconstruct the original data from the noise.

Text-to-Image Models: Text-to-image models, such as DALL-E by OpenAI, generate images from textual descriptions, leveraging advanced understanding of both language and visual content. These models typically combine aspects of autoregressive models, transformers, and sometimes GANs or diffusion models to create detailed and contextually relevant images based on text inputs. They exemplify the intersection of natural language processing and computer vision, enabling creative and practical applications like art generation, design, and more.

Now, variational autoencoders are generally popularly used to create synthetic data, by understanding the underlying probability distribution the data comes from. This involves not only recognizing patterns and features in the data but also understanding the variability and randomness inherent in it. For example, a generative model trained on images of faces learns

not just common features of faces but also the variations (like different expressions, angles, lighting conditions). Once a generative model has learned the distribution, it can generate new data samples that are statistically like the original data. These new samples are not mere copies but are new creations that reflect the learned distribution. The ability to generate new samples is crucial. It means the model can produce a diverse range of outputs, each reflecting different aspects of the learned distribution. This is particularly useful in fields like art generation, drug discovery, and any domain where novel, yet plausible, data creation is valuable. Non-generative models, such as standard regression or classification models, focus on predicting specific outcomes based on input data. They are typically used when the goal is to make a prediction (like price prediction) or to classify data into predefined categories (like spam detection). These models are generally limited to the scope of their training data and labels. They excel in tasks where the goal is to find a direct mapping from inputs to outputs but don't inherently learn the full distribution of the data.

For our project, our aim is to use machine learning as a transfer function generator. This transfer function is what we call the characteristic curve maps the relation between the leaf RWC and the output voltage from the PLP device: V_{TRx}. Now since every leaf that occurs in the nature is significantly different from both phenotypic and genotypic standpoint, it is imperative to be able to understand and learn as how these differences create variations in the relationship between the RWC in a particular leaf and the sensor readout voltage. This includes not only learning the complex non-linear relationship connecting the machine learning features (leaf geometry and cultivar for our case) to the labels such as the V_{TRx} vs. the RWC curve, but also understanding how minute variations in features due to the inherent nature's randomness leads to totally different V_{TRx} – RWC curves. This calls for the need to use a model which can map the stochasticity present in the data into a probabilistic space and thereby able to learn the underlying probability distribution of data thereby accounting for all the miniscule nature's variations. Such a model will have to be generative in nature and will predict similar but diverse VTRx – RWC curves for each iteration and will be able to match the ground-truth curve with the least error possible. No non-generative model will be able to do this since the inability of such models to learn the underlying probability distribution of the data.

C. Steps to find leaf features such as the area, perimeter and skewness

In order to find the geometric features such as the area, perimeter and skewness of a particular leaf, Image J was used, and the following steps were followed:

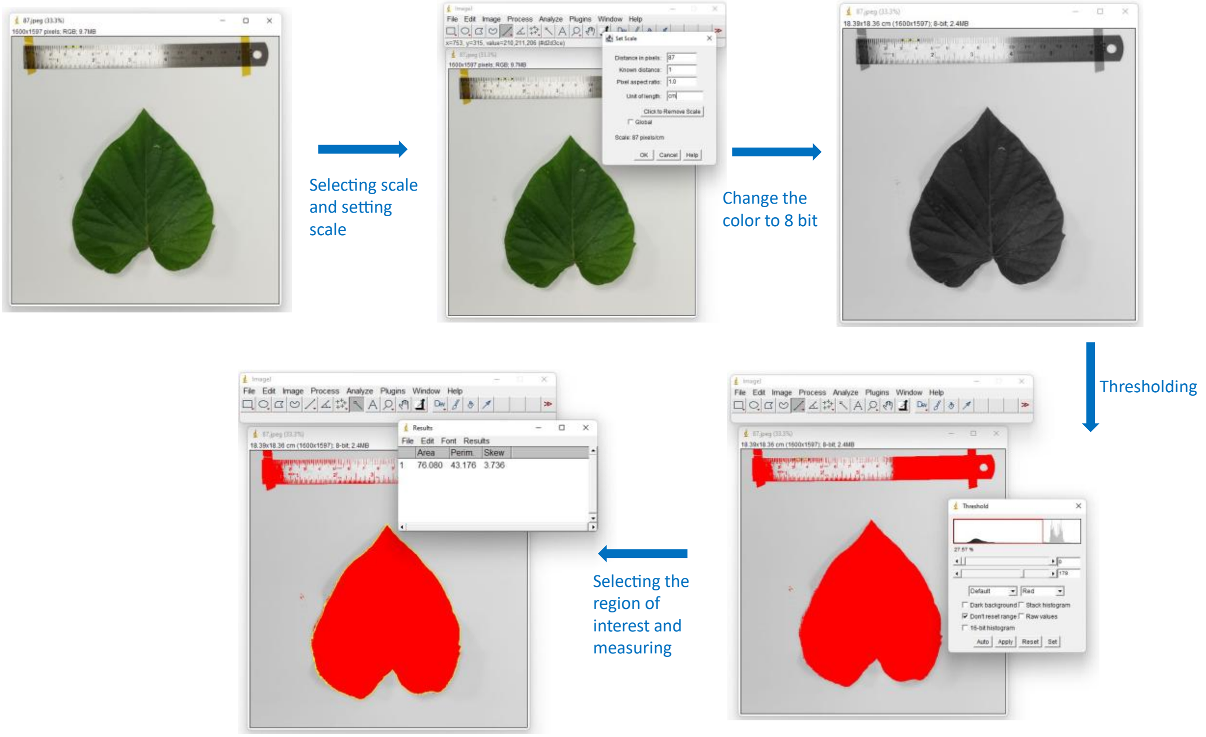

Supplementary Figure S23: Pictorial flowchart showing the steps to find the area, perimeter and skewness of a leaf using image processing software. These three features along with the other feature such as the thickness are used as the features of the machine learning algorithm.

Step 1: Selecting a white background with a scale included. It's important to select a background to which the object under consideration will have a colour contrast.

Step 2: Connecting image pixel to the scale. This is done since the final measurement of features is achieved by the software by measuring the number of pixels associated with the leaf.

Step 3: Changing the picture colour to 8 bits black and white for attaining higher contrast.

Step 4: Thresholding which is a technique of dividing an image into two or more classes of pixels which are typically called the foreground and the background is then carried out.

Step 5: Selecting the region of interest by the previously divided pixels in order to present the measured features.

D. Leaf probing zones for obtaining training data

Probing was done to obtain the (a) leaf thickness by employing vernier calliper and (b) to place the PLP on the leaves to measure the RWC through the sensor readout voltage. Leaf thickness was measured 5 different times and averaged to find out the mean thickness which was then

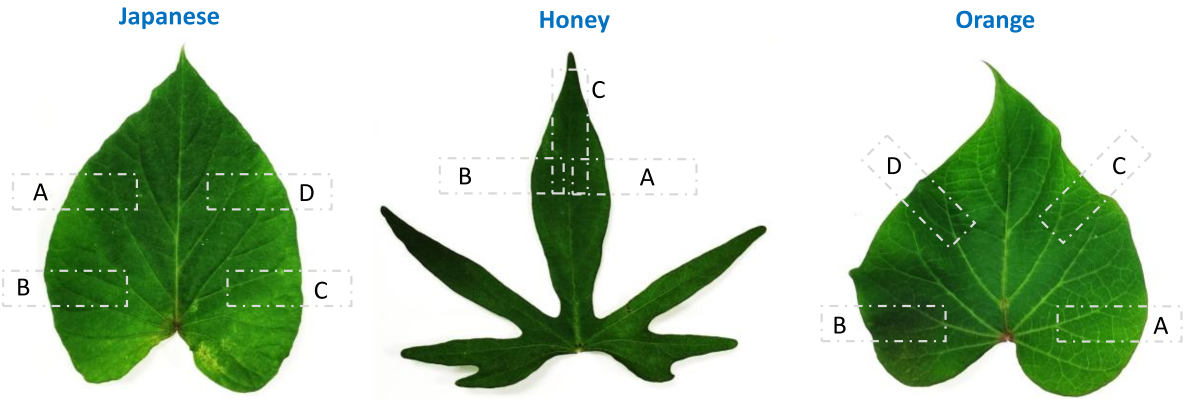

Supplementary Figure S24: Probing zones for leaves from different cultivars: Japanese, Honey, and Orange.

used as the feature input in the machine learning model. For measuring the PLP's readout voltage, the PLP was placed carefully in the designated section of the spacer, designed to contain the leaf blade. Care was taken to prevent any portion of the leaf blade from sliding into the interlock region of the spacer, since that would hinder perfect closure of the PLP and also might destroy some portion of the leaf blade. The Japanese and the Orange cultivars have 4 probing points due to its broader leaf architecture whereas the Honey cultivar has 3 probing points due to comparatively smaller leaf architecture.

E. Looks of a sample data set for training the ML model

The training data for developing the machine learning model consists of measuring sensor

1	Read Number@2.5 min	Mass (mg)	RWC %	VTRx_1 (mV)	VTRx_2 (mV)	VTRx_3 (mV)	Mean VTRx (mV)	SD VTRx (mV)	Area (cm^2)	Perimeter (cm)	A/P (cm)	Thickness Mean (um)	Thickness SD	Skewness
2														
3	1	617	100.00	136.43	126.63	140.95	134.67	5.98	37.65	76.45	0.49	86	16.2	4
4	2	595	95.63	155.4	142.84	131.28	143.17	9.85						
5	3	522	81.11	167.71	139.82	148.37	151.97	11.67						
6	4	488	74.35	193.34	161.56	172.24	175.71	13.2						
7	5	460	68.79	193.09	168.84	184.3	182.08	10.02						
8	6	433	63.42	214.82	199.37	191.33	201.84	9.75						
9	7	413	59.44	66.21	199.12	197.74	154.36	62.33						
10	8	388	54.47	229.15	214.57	197.24	213.65	13.04				Honey		
11	9	372	51.29	209.55	204.02	208.29	207.29	2.37						
12	10	345	45.92	238.94	211.06	217.34	222.45	11.94						
13														
14														
15	24 hrs	114												
16														

Label data   Feature data

1	Read Number@2.5 mir	Mass (mg)	RWC %	VTRx_1 (mV)	VTRx_2 (mV)	VTRx_3 (mV)	VTRx_4 (mV)	Mean VTRx (mV)	SD VTRx (mV)	Area (cm^2)	Perimeter (cm)	A/P (cm)	Thickness Mean (um)	Thickness SD	Skewness
2															
3	1	845	100.00	105.4	106.9	113.57	101.63	106.88	4.32	43.11	29.6	1.46	132	11.6	1.54
4	2	746	86.46	112.44	114.95	103.77	117.84	112.25	5.26						
5	3	653	73.73	121.36	127.14	124.12	131.16	125.94	3.64						
6	4	557	60.60	124.25	129.4	121.48	139.07	128.55	6.71						
7	5	483	50.48	136.81	139.45	119.1	139.45	133.7	8.5						
8	6	428	42.95	157.04	127.14	138.69	156.28	144.79	12.56						
9	7	375	35.70	158.04	166.83	144.22	151.26	155.09	8.36						
10	8	336	30.37	162.56	167.09	155.78	160.05	161.37	4.1			Japanese / Orange			
11	9	306	26.27	220.85	182.16	211.31	173.62	196.98	19.62						
12	10	283	23.12	207.29	174.37	171.61	192.21	186.37	14.44						
13															
14															
15	24 hrs	114													
16															

Supplementary Figure S25: Sample training dataset for various cultivars of sweet potato.

readout voltage from 200 different leaves: 100 leaves from 3 different plants belonging to the Honey cultivar, 50 leaves from 3 different plants from the Japanese cultivar and 50 different leaves from 3 different plants from the Orange cultivar. Fig. S24 represents the look of two such datasets randomly picked from the collection of 200 datasets corresponding to 200 different leaves. As is evident, the first column of each dataset is the read number at 2.5 minutes, and 10 of such data is collected. Now 2.5 minutes is the time for which each leaf was heated in a muffled furnace at 60°C. The uniform heating ensures uniform loss of water through the leaf. Since leaves mostly have their stomata at the bottom, each leaf was placed upside-down in the furnace for efficient loss of water. The next column is the mass of the leaf as observed after each heating iteration. The next column is the RWC which was measured the day following the day of data collection since the leaf required 24 hours of heating to be in the complete dried state. The mass corresponding to the 24 hours of heating was collected and is also reported in the dataset in the row number 15. The next three columns for Honey cultivar and the next 4 columns for the Japanese and the Orange cultivar is the VTRx values which is the sensor readout voltage obtained after each heating iteration. The next two columns represent the mean and the standard deviation as obtained from the VTRx values. The grey coloured vertical line demarcates the end of collection of the data for the label data and the beginning of feature data. The feature data consists of 5 columns such as the area, perimeter, area to perimeter ratio, thickness, and skewness. There is an extra column for the thickness mean to get a feel in the variation of observed thickness in various leaves.

F. Model training curves

From the dataset as depicted in Fig. S24, V_{TRx} is plotted with respect to the leaf RWC in order to obtain the characteristic curves for 200 different leaves: 100 from the Honey cultivar, 50

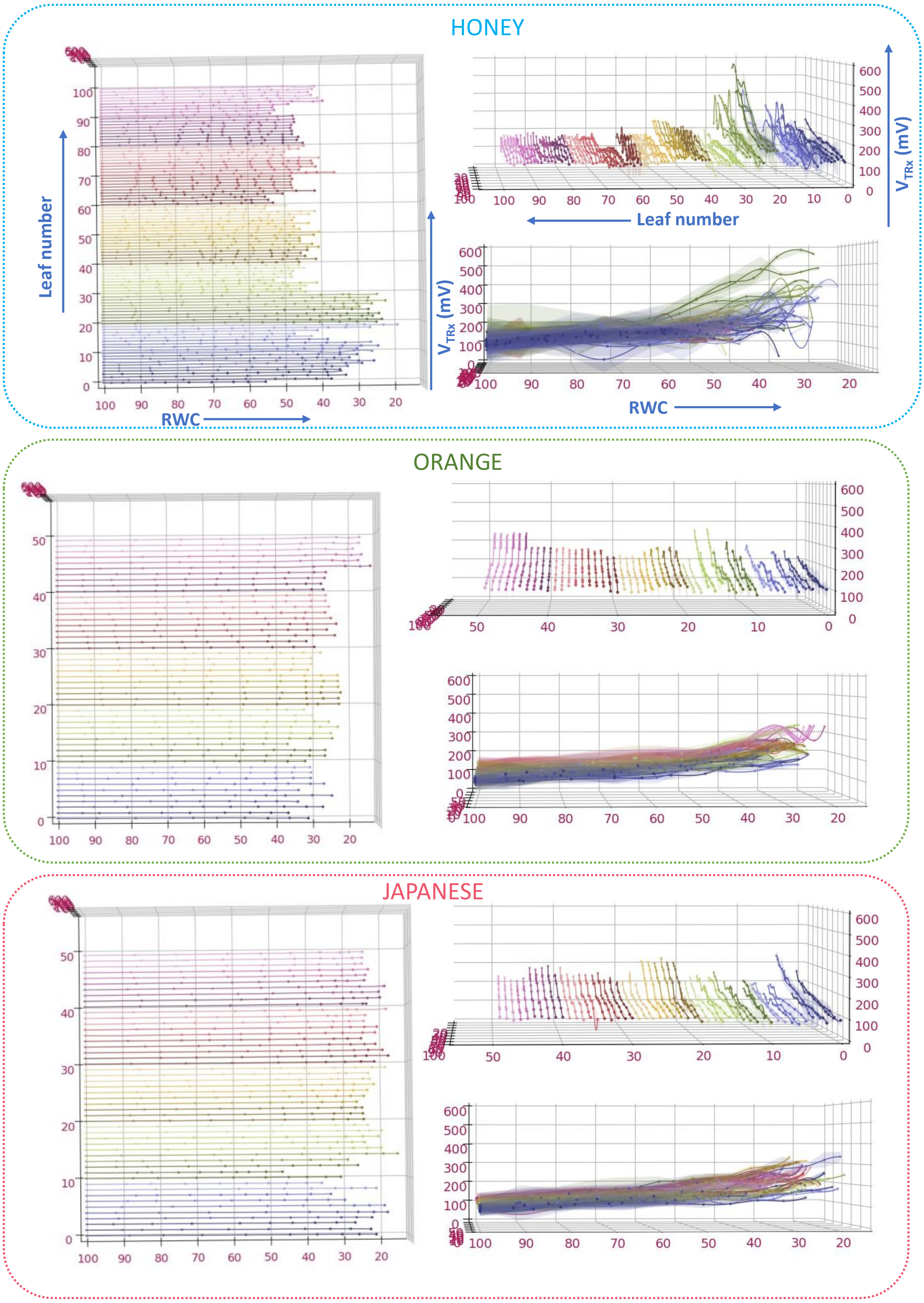

Supplementary Figure S26: Characteristic training curves from different angles for clarity.

from the Orange cultivar and another 50 from the Japanese cultivar respectively. Different perspectives of such plots are being depicted in Fig. S25. From the RWC point of view, the leaves from the Honey cultivar can be classified into two groups: Group A with RWC varying from 100% to 40% and group B with the RWC varying from 100% to 20%. Although there is not a clear explanation to the reason behind such an observation, it depicts that there might even exist genotypical variation in different plants from the same cultivar of sweet potato. Such kind of classification was not observed for Orange or Japanese cultivars. From the V_{TRx} point of view, the leaves from the Honey cultivar can be classified into two groups: Group A having the maximum range of V_{TRx} values as 300 mV, and group B having maximum range of V_{TRx} values as 600 mV respectively. These values are non-linearly related to the minimum respective values of RWC. In addition, a larger variance of the V_{TRx} values is evident from the Fig. S25. For the Orange and the Japanese cultivars, the maximum range of V_{TRx} values lie in the range of 0 – 400 mV respectively with a lesser variance as compared to the Honey cultivar.

G. Structure of the CVAE ML model

We use the conditional variation autoencoder (CVAE) [8], [68], [69] as our machine learning model. Initially the data in the form of a 2D matrix consisting of 200 row-column RWC- V_{TRx} values is divided into 200 separate rows and columns, thereby dividing the 2D matrix into 200 1D vectors having row and column values held separately. As can be seen, form Fig. S26, the

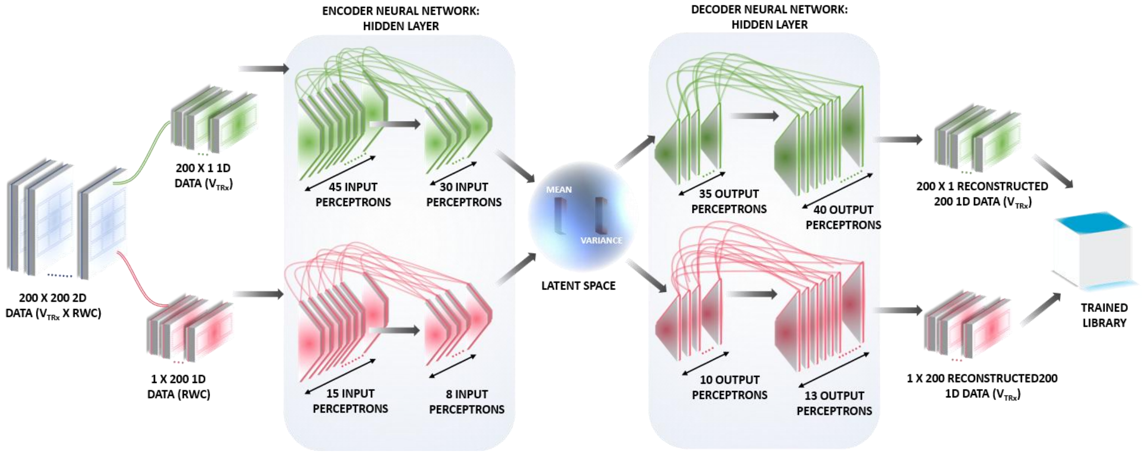

Supplementary Figure S27: Structure of the machine learning model used in the contribution.

model has been trained to generate the x-axis and the y-axis data separately by using the encoder and decoder neural networks. The separated row and column data is next fed to encoder and decoder neural networks. The encoder network used to compress data in the V_{TRx} case, consists of two hidden layers having 45 and 30 perceptrons respectively, and the decoder network that was used to decompress the data consists of two hidden layers having 35 and 40 output perceptrons respectively. For the RWC case, the encoder network used to compress data consists of two hidden having 15 and 8 perceptrons respectively, and the decoder network that used to decompress the data consists of two hidden layers having 10 and 13 perceptrons respectively. The data after encoding was compressed into the latent space in which the data was represented as a probability distribution having mean and variance as the key elements describing the distribution. The learning rate for both the V_{TRx} and RWC cases were 1e-3. A batch size of 64 was maintained. Loss function was calculated for 10000 epochs. Activation function used was sigmoid. Weight initialization was random. Optimizer used was Adam. Loss function used was reconstruction loss and Kullback-Leibler divergence.

H. Effect of increase in training data on ML prediction

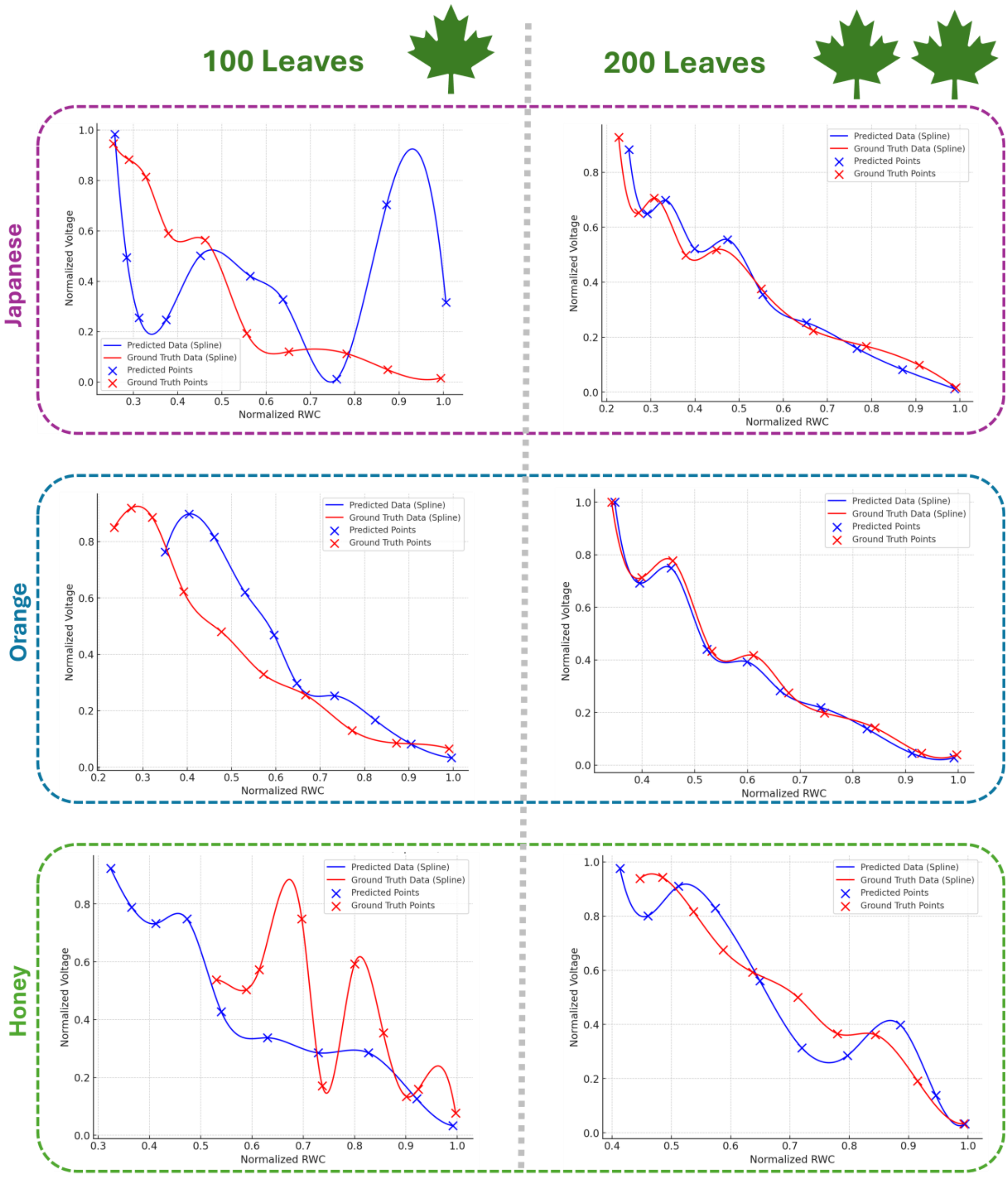

Supplementary Figure S28: Machine learning performance improvement on enhancing the number of leaves from 100 to 200.

We tested our ML platform to verify the sufficiency of the number of leaves to correctly predict the RWC. For 100 leaves (Honey: 50, Japanese: 20, Orange: 20) the predicted trend and the ground truth trend did not match, suggesting the need for collection of more data. Thus, we increased the net number of leaves to 200 (Honey: 100, Japanese: 50, Orange: 50), and observed the model to perform better. Although the model can be furthermore strengthened by

collecting more data, but such a requirement is currently out of the scope of the manuscript, since the model is believed to perform sufficiently well for the proof-of-concept demonstration.

I. Experimental setup for whole plant experiments

The growth chamber based experimental setup used to demonstrate the capability of the generative-AI assisted PLP device to accurately track the hydration status of a particular leaf

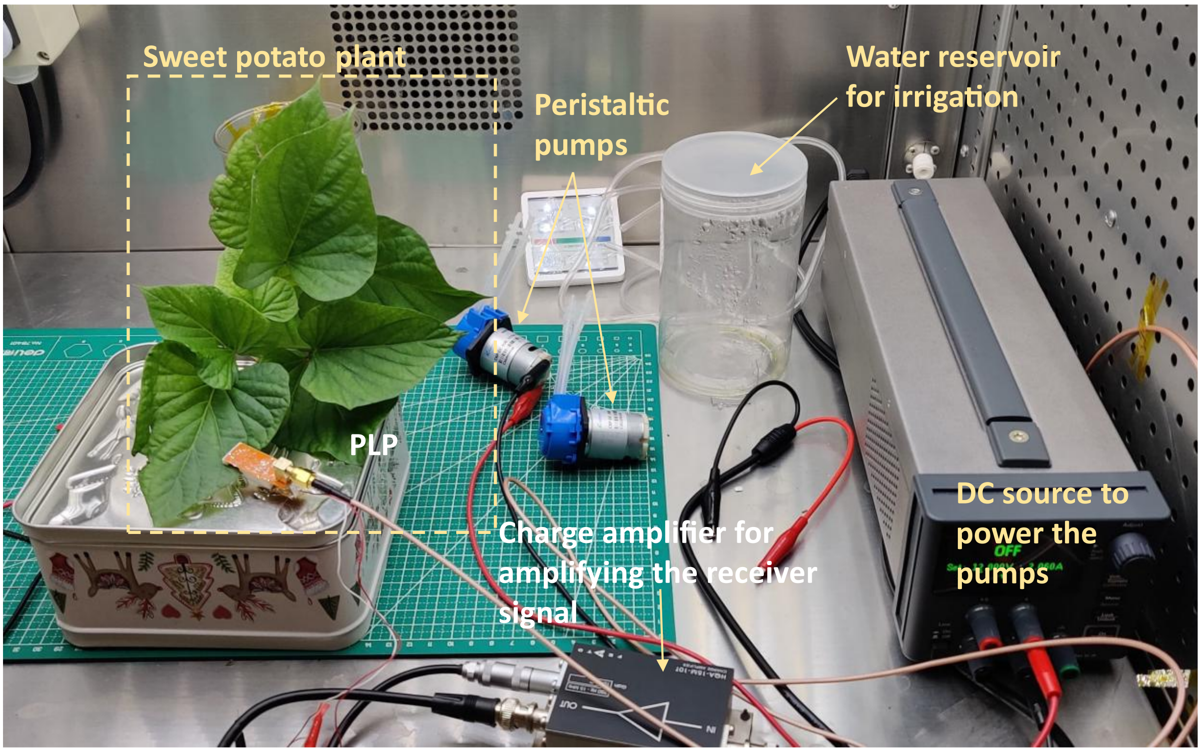

Supplementary Figure S29: Experimental setup for the whole plant experiment showing the constitutive parts.

in real-time is depicted in Fig. S29. As can be seen, the PLP device is attached to one of the Japanese sweet potato leaf which is in-turn detached from the soil, with the root system intact and placed in a glass beaker. The setup contains a water reservoir and two peristaltic pumps to mimic irrigation and circulate water from the water reservoir to the beaker and vice versa. A DC voltage source is used to power up the peristaltic pumps during their operational time. A digital environment conditions monitor (temperature and humidity) is also placed inside the chamber in order to real-time verify the temperature and humidity conditions provided as input to the growth chamber controller. The PLP is operated using an arbitrary waveform generator from Agilent Inc. and the device voltage readout (V_{TRx}) is picked up using the oscilloscope and recorded with the passage of every 2.5 minutes.

References

[1] F. J. Pierce and P. Nowak, "Aspects of precision agriculture," *Adv. Agron.*, vol. 67, pp. 1–85, 1999.

[2] U. Shafi, R. Mumtaz, J. Garc\'\ia-Nieto, S. A. Hassan, S. A. R. Zaidi, and N. Iqbal, "Precision agriculture techniques and practices: From considerations to applications," *Sensors*, vol. 19, no. 17, p. 3796, 2019.

[3] G. Lee, Q. Wei, and Y. Zhu, "Emerging wearable sensors for plant health monitoring," *Adv. Funct. Mater.*, vol. 31, no. 52, p. 2106475, 2021.

[4] C.-C. Qu, X.-Y. Sun, W.-X. Sun, L.-X. Cao, X.-Q. Wang, and Z.-Z. He, "Flexible wearables for plants," *Small*, vol. 17, no. 50, p. 2104482, 2021.

[5] J. Jarzynski, "Mechanisms of sound attenuation in materials," ACS Publications, 1990.

[6] T. E. Gomez Alvarez-Arenas, "Air-coupled ultrasonic transducers," *Ultrasound Food Process. Recent Adv.*, pp. 175–228, 2017.

[7] K. Roy, J. E.-Y. Lee, and C. Lee, "Thin-film PMUTs: a review of over 40 years of research," *Microsystems \& Nanoeng.*, vol. 9, no. 1, p. 95, 2023.

[8] L. Girin, S. Leglaive, X. Bie, J. Diard, T. Hueber, and X. Alameda-Pineda, "Dynamical variational autoencoders: A comprehensive review," *arXiv Prepr. arXiv2008.12595*, 2020.

[9] Y. Koh *et al.*, "High resolution, high frequency ultrasonic ranging in air with pMUTs," in *2021 IEEE International Ultrasonics Symposium (IUS)*, 2021, pp. 1–4.

[10] J. Liu *et al.*, "Sputtered PZT pMUT with Bias-Tunable Electromechanical Coupling Coefficient for Air-coupled Ranging Applications," in *2022 IEEE International Ultrasonics Symposium (IUS)*, 2022, pp. 1–4.

[11] T. Liu *et al.*, "Airborne Rangefinding With pMUTs Array Using Differential Structure," *IEEE Sens. J.*, 2023.

[12] R. J. Przybyla *et al.*, "In-air rangefinding with an aln piezoelectric micromachined ultrasound transducer," *IEEE Sens. J.*, vol. 11, no. 11, pp. 2690–2697, 2011.

[13] H. Gupta, B. Nayak, A. Ashok, and R. Pratap, "Data-Over-Sound With PMUTs,"

IEEE Open J. Ultrason. Ferroelectr. Freq. Control, vol. 2, pp. 152–161, 2022.

[14] M. Billen, P. Gijsenbergh, E. M. Ferrer, M. S. Pandian, X. Rottenberg, and V. Rochus, "PMUT Array Design for mid-air Haptic feedback," in *2022 23rd International Conference on Thermal, Mechanical and Multi-Physics Simulation and Experiments in Microelectronics and Microsystems (EuroSimE)*, 2022, pp. 1–6.

[15] S. Pala, Z. Shao, Y. Peng, and L. Lin, "Ultrasond-induced haptic sensations via PMUTS," in *2021 IEEE 34th International Conference on Micro Electro Mechanical Systems (MEMS)*, 2021, pp. 911–914.

[16] T. Wang, T. Kobayashi, B. Yang, H. Wang, and C. Lee, "Highly sensitive piezoelectric micromachined ultrasonic transducer (pMUT) operated in air," in *2016 IEEE 11th Annual International Conference on Nano/Micro Engineered and Molecular Systems (NEMS)*, 2016, pp. 294–299.

[17] J. Su, X. Zhang, G. Zhou, C. Xia, W. Zhou, and others, "A review: crystalline silicon membranes over sealed cavities for pressure sensors by using silicon migration technology," *J. Semicond.*, vol. 39, no. 7, p. 71005, 2018.

[18] R. Kant, *Silicon migration as a process for micro/nanofabrication*. Stanford University, 2009.

[19] D. S. W. Choong *et al.*, "Silicon-on-nothing scaln pmuts," in *2021 IEEE International Ultrasonics Symposium (IUS)*, 2021, pp. 1–4.

[20] J. Sharma *et al.*, "Piezoelectric over Silicon-on-Nothing (pSON) process," in *2021 IEEE International Ultrasonics Symposium (IUS)*, 2021, pp. 1–4.

[21] D. Sancho-Knapik *et al.*, "The application of leaf ultrasonic resonance to Vitis vinifera L. suggests the existence of a diurnal osmotic adjustment subjected to photosynthesis," *Front. Plant Sci.*, vol. 7, p. 1601, 2016.

[22] J. J. Kim, L. K. Allison, and T. L. Andrew, "Vapor-printed polymer electrodes for long-term, on-demand health monitoring," *Sci. Adv.*, vol. 5, no. 3, p. eaaw0463, 2019.

[23] M. Fawakherji, C. Potena, A. Pretto, D. D. Bloisi, and D. Nardi, "Multi-spectral image synthesis for crop/weed segmentation in precision farming," *Rob. Auton. Syst.*, vol. 146, p. 103861, 2021.

[24] M. Fawakherji, C. Potena, I. Prevedello, A. Pretto, D. D. Bloisi, and D. Nardi, "Data augmentation using GANs for crop/weed segmentation in precision farming," in *2020 IEEE Conference on Control Technology and Applications (CCTA)*, 2020, pp. 279–284.

[25] Y. Akkem, S. K. Biswas, and A. Varanasi, "A comprehensive review of synthetic data generation in smart farming by using variational autoencoder and generative adversarial network," *Eng. Appl. Artif. Intell.*, vol. 131, p. 107881, 2024.

[26] C.-B. Eom and S. Trolier-McKinstry, "Thin-film piezoelectric MEMS," *MRS Bull.*, vol. 37, no. 11, pp. 1007–1017, 2012, doi: 10.1557/mrs.2012.273.

[27] S.-G. Kim, S. Priya, and I. Kanno, "Piezoelectric MEMS for energy harvesting," *MRS Bull.*, vol. 37, no. 11, pp. 1039–1050, 2012.

[28] P. Muralt, "Recent progress in materials issues for piezoelectric MEMS," *J. Am. Ceram. Soc.*, vol. 91, no. 5, pp. 1385–1396, 2008.

[29] S. Tadigadapa and K. Mateti, "Piezoelectric MEMS sensors: state-of-the-art and perspectives," *Meas. Sci. Technol.*, vol. 20, no. 9, p. 92001, 2009.

[30] Y. Qiu *et al.*, "Piezoelectric Micromachined Ultrasound Transducer (PMUT) Arrays for Integrated Sensing, Actuation and Imaging," *Sensors*, vol. 15, no. 4, pp. 8020–8041, 2015, doi: 10.3390/s150408020.

[31] J. Jung, W. Lee, W. Kang, E. Shin, J. Ryu, and H. Choi, "Review of piezoelectric micromachined ultrasonic transducers and their applications," *J. Micromechanics Microengineering*, vol. 27, no. 11, 2017, doi: 10.1088/1361-6439/aa851b.

[32] L. González and M. González-Vilar, "Determination of relative water content," in *Handbook of plant ecophysiology techniques*, Springer, 2001, pp. 207–212.

[33] R. E. Smart and G. E. Bingham, "Rapid estimates of relative water content," *Plant Physiol.*, vol. 53, no. 2, pp. 258–260, 1974.

[34] T. Wang, R. Sawada, and C. Lee, "A piezoelectric micromachined ultrasonic transducer using piston-like membrane motion," *IEEE Electron Device Lett.*, vol. 36, no. 9, pp. 957–959, 2015.

[35] S. Akhbari, F. Sammoura, C. Yang, M. Mahmoud, N. Aqab, and L. Lin, "Bimorph

pMUT with dual electrodes," in *2015 28th IEEE International Conference on Micro Electro Mechanical Systems (MEMS)*, Jan. 2015, pp. 928–931, doi: 10.1109/MEMSYS.2015.7051112.

[36] S. Akhbari, A. Voie, Z. Li, B. Eovino, and L. Lin, "Dual-electrode bimorph pmut arrays for handheld therapeutic medical devices," in *2016 IEEE 29th International Conference on Micro Electro Mechanical Systems (MEMS)*, Jan. 2016, pp. 1102–1105, doi: 10.1109/MEMSYS.2016.7421827.

[37] M. Ji, H. Yang, Y. Zhou, X. Xiu, H. Lv, and S. Zhang, "Bimorph Dual-Electrode ScAlN PMUT with Two Terminal Connections," *Micromachines*, vol. 13, no. 12, p. 2260, 2022.

[38] K. Roy, A. Mandal, A. Ashok, H. Gupta, V. Shastri, and R. Pratap, "A Single Cell PMUT as a Bio-Fluid Density Sensor," *IEEE Int. Ultrason. Symp. IUS*, vol. 2020-Septe, no. c, pp. 11–14, 2020, doi: 10.1109/IUS46767.2020.9251809.

[39] S. J. Rothberg *et al.*, "An international review of laser Doppler vibrometry: Making light work of vibration measurement," *Opt. Lasers Eng.*, vol. 99, pp. 11–22, 2017.

[40] R. L. Goode, G. Ball, S. Nishihara, and K. Nakamura, "Laser doppler vibrometer (ldv)," *Otology \& Neurotology*, vol. 17, no. 6. LWW, pp. 813–822, 1996.

[41] C. Rembe, G. Siegmund, H. Steger, and M. Wörtge, "Measuring MEMS in motion by laser Doppler vibrometry," in *Optical Inspection of Microsystems, Second Edition*, CRC Press, 2019, pp. 297–347.

[42] A. Dangi and R. Pratap, "System level modeling and design maps of PMUTs with residual stresses," *Sensors Actuators, A Phys.*, vol. 262, pp. 18–28, 2017, doi: 10.1016/j.sna.2017.05.006.

[43] W. Wang *et al.*, "High performance AlScN thin film based surface acoustic wave devices with large electromechanical coupling coefficient," *Appl. Phys. Lett.*, vol. 105, no. 13, 2014.

[44] J. Cho, M. Anderson, R. Richards, D. Bahr, and C. Richards, "Optimization of electromechanical coupling for a thin-film PZT membrane: I. Modeling," *J. Micromechanics Microengineering*, vol. 15, no. 10, p. 1797, 2005.

[45] Q. Chen and Q.-M. Wang, “The effective electromechanical coupling coefficient of piezoelectric thin-film resonators,” *Appl. Phys. Lett.*, vol. 86, no. 2, 2005.

[46] W. Liu *et al.*, “3D FEM analysis of high-frequency AlN-based PMUT arrays on cavity SOI,” *Sensors*, vol. 19, no. 20, p. 4450, 2019.

[47] O. M. O. Abdalla, G. Massimino, A. S. Savoia, F. Quaglia, and A. Corigliano, “Efficient modeling and simulation of pmut arrays in various ambients,” *Micromachines*, vol. 13, no. 6, p. 962, 2022.

[48] M. D. Fariñas and T. E. G. Álvarez-Arenas, “Ultrasonic assessment of the elastic functional design of component tissues of Phormium tenax leaves,” *J. Mech. Behav. Biomed. Mater.*, vol. 39, pp. 304–315, 2014.

[49] L. E. Kinsler, A. R. Frey, A. B. Coppens, and J. V Sanders, *Fundamentals of acoustics*. John wiley \& sons, 2000.

[50] H. G. Choi, B. Y. Moon, and N. J. Kang, “Effects of LED light on the production of strawberry during cultivation in a plastic greenhouse and in a growth chamber,” *Sci. Hortic. (Amsterdam).*, vol. 189, pp. 22–31, 2015.

[51] J. R. De Freitas and J. J. Germida, “Growth promotion of winter wheat by fluorescent pseudomonads under growth chamber conditions,” *Soil Biol. Biochem.*, vol. 24, no. 11, pp. 1127–1135, 1992.

[52] J. G. Carbonell, R. S. Michalski, and T. M. Mitchell, “An overview of machine learning,” *Mach. Learn.*, pp. 3–23, 1983.

[53] S. B. Kotsiantis, I. Zaharakis, P. Pintelas, and others, “Supervised machine learning: A review of classification techniques,” *Emerg. Artif. Intell. Appl. Comput. Eng.*, vol. 160, no. 1, pp. 3–24, 2007.

[54] K. G. Liakos, P. Busato, D. Moshou, S. Pearson, and D. Bochtis, “Machine learning in agriculture: A review,” *Sensors*, vol. 18, no. 8, p. 2674, 2018.

[55] B. Mahesh, “Machine learning algorithms-a review,” *Int. J. Sci. Res. (IJSR).[Internet]*, vol. 9, no. 1, pp. 381–386, 2020.

[56] Y. LeCun, Y. Bengio, and G. Hinton, “Deep learning,” *Nature*, vol. 521, no. 7553, pp. 436–444, 2015.

[57] P. P. Shinde and S. Shah, “A review of machine learning and deep learning applications,” in *2018 Fourth international conference on computing communication control and automation (ICCUBEA)*, 2018, pp. 1–6.

[58] R. Vargas, A. Mosavi, and R. Ruiz, “Deep learning: a review,” 2017.

[59] A. Bandi, P. V. S. R. Adapa, and Y. E. V. P. K. Kuchi, “The power of generative ai: A review of requirements, models, input--output formats, evaluation metrics, and challenges,” *Futur. Internet*, vol. 15, no. 8, p. 260, 2023.

[60] Y. Cao *et al.*, “A comprehensive survey of ai-generated content (aigc): A history of generative ai from gan to chatgpt,” *arXiv Prepr. arXiv2303.04226*, 2023.

[61] S. Kumar, D. Musharaf, S. Musharaf, and A. K. Sagar, “A Comprehensive Review of the Latest Advancements in Large Generative AI Models,” in *International Conference on Advanced Communication and Intelligent Systems*, 2023, pp. 90–103.

[62] L. Alzubaidi *et al.*, “Review of deep learning: Concepts, CNN architectures, challenges, applications, future directions,” *J. big Data*, vol. 8, pp. 1–74, 2021.

[63] A. Sherstinsky, “Fundamentals of recurrent neural network (RNN) and long short-term memory (LSTM) network,” *Phys. D Nonlinear Phenom.*, vol. 404, p. 132306, 2020.

[64] R. Asadi and S. A. Kareem, “Review of feed forward neural network classification preprocessing techniques,” in *AIP Conference Proceedings*, 2014, vol. 1602, no. 1, pp. 567–573.

[65] A. M. Hemeida *et al.*, “Nature-inspired algorithms for feed-forward neural network classifiers: A survey of one decade of research,” *Ain Shams Eng. J.*, vol. 11, no. 3, pp. 659–675, 2020.

[66] A. Borji, “Pros and cons of gan evaluation measures,” *Comput. Vis. image Underst.*, vol. 179, pp. 41–65, 2019.

[67] G. M. Harshvardhan, M. K. Gourisaria, M. Pandey, and S. S. Rautaray, “A comprehensive survey and analysis of generative models in machine learning,” *Comput. Sci. Rev.*, vol. 38, p. 100285, 2020.

[68] M. Lopez-Martin, B. Carro, A. Sanchez-Esguevillas, and J. Lloret, “Conditional variational autoencoder for prediction and feature recovery applied to intrusion

detection in iot," *Sensors*, vol. 17, no. 9, p. 1967, 2017.

[69] A. A. Pol, V. Berger, C. Germain, G. Cerminara, and M. Pierini, "Anomaly detection with conditional variational autoencoders," in *2019 18th IEEE international conference on machine learning and applications (ICMLA)*, 2019, pp. 1651 1657.

[70] T. G. Alvarez-Arenas, "Determination of plant leaves water status using air-coupled ultrasounds," in *2009 IEEE International Ultrasonics Symposium*, 2009, DOI: 10.1109/ULTSYM.2009.5441754

[71] Jae Joon Kim et al.,"Vapor-printed polymer electrodes for long-term, on-demand health monitoring," *Sci. Adv.*5, eaaw0463(2019). DOI:10.1126/sciadv.aaw0463

[72] Zimmermann et al., "A novel, non-invasive, online-monitoring, versatile and easy plant-based probe for measuring leaf water status," *Journal of Experimental Botany*, Volume 59, Issue 11, August 2008, Pages 3157–3167, https://doi.org/10.1093/jxb/ern171

[73] Hunt et al., "Measurement of Leaf Relative Water Content by Infrared Reflectance," *Remote Sensing of Environment*, Volume 22, Pages 429–435

[74] Farinas et al., "Instantaneous and non-destructive relative water content estimation from deep learning applied to resonant ultrasonic spectra of plant leaves," *Plant methods*, Volume 15, Issue: 128, DOI: 10.1186/s13007-019-0511-z

[75] Atherton et al., "A leaf-mounted thermal sensor for the measurement of water content," *Sensors and Actuators A: Physical*, Volume 187, pp. 67–72 , DOI: 10.1016/j.sna.2012.06.021

[76] Yin et al., "Plant Tattoo Sensor Array for Leaf Relative Water Content, Surface Temperature, and Bioelectric Potential Monitoring," *Adv. Mater. Technol.* 2024, 9, 2302073

[77] Li et al. "Non-invasive measurement of leaf water content and pressure–volume curves using terahertz radiation," *Sci Rep,* 10, 21028 (2020), DOI: 10.1038/s41598-020-78154-z

[78] Zheng et al. "Real-time evaluation of corn leaf water content based on the electrical property of leaf," *Computers and Electronics in Agriculture,* pp. 112, 102–109 (2015),

DOI: 10.1016/j.compag.2014.11.007

[79] Afzal et al. "Leaf thickness to predict plant water status," *Biosystems Engineering,* 156, pp. 148–156 (2017), DOI: 10.1016/j.biosystemseng.2017.01.011
